# Automation and the changing nature of work

**DOI:** 10.1371/journal.pone.0266326

**Published:** 2022-05-05

**Authors:** Cecily Josten, Grace Lordan

**Affiliations:** Department of Psychological and Behavioural Science, The London School of Economics and Political Science, London, United Kingdom; Al Mansour University College-Baghdad-Iraq, IRAQ

## Abstract

This study identifies the job attributes, and in particular skills and abilities, which predict the likelihood a job is recently automatable drawing on the Josten and Lordan (2020) classification of automatability, EU labour force survey data and a machine learning regression approach. We find that skills and abilities which relate to non-linear abstract thinking are those that are the safest from automation. We also find that jobs that require ‘people’ engagement interacted with ‘brains’ are also less likely to be automated. The skills that are required for these jobs include soft skills. Finally, we find that jobs that require physically making objects or physicality more generally are most likely to be automated unless they involve interaction with ‘brains’ and/or ‘people’.

## 1. Introduction

Research on the automation and the future of work has brought with it a range of research contributions, which seek to determine which occupations will be lost to automation. For example, Frey and Osborne [1] estimate the susceptibility of occupations to computerization and find that 47% of US occupations are at risk of automation, and point to service jobs as being susceptible to automation. Many other contributions in the automation literature rely on defining automatable work through measures of the tasks associated with a particular occupation rather than the occupation overall to get a more nuanced understanding of the impact of automation on employment [2–4]. One of the most prominent is owed to Autor and Dorn [5] and Autor et al. [6] who define a job as automatable if it is high in routine task-intensity. Specifically, routine task-intensity is defined based on how high a job ranks on routine content, and how low it ranks on abstract and manual content. Information on the routine, abstract and manual task content of each respective occupation comes from the US *Dictionary of Occupation Titles* where incumbents are asked to grade their occupation with respect to particular attributes. A job is then defined as automatable if it is in the top third of the distribution of routine task-intensity. This measure of automatable work has followed the big movements in the occupation distribution accurately over the last decades–namely the hollowing out of the middle of the occupational distribution [7]. To this end the types of occupations available have become more polarized, with the majority of occupations falling into high and low skill categories, and mid skill jobs disappearing in numbers [8].

Further, Arntz et al. [2] estimate the risk of automation for 21 OECD countries also using a task-based approach. They argue that other studies using an occupation rather than a task-based approach such as Frey and Osborne [1] overestimate the risk of automation, partly due to the fact that cross-country variation is not taken into account other than through differences in occupation structure. They highlight that technical possibilities of automation do not equate to actual automation because that may be hindered by legal or ethical obstacles. They find that overall only 9% of jobs are automatable. Similarly, Nedelkoska and Quintini [9] also study automation of jobs in OECD countries using the Survey of Adult Skills (PIAAC). They focus on very detailed occupational categories and the tasks therein and find that 14% of jobs in OECD countries are at high risk of being automated and 32% have a probability of being automated of 50% to 70%. Gregory et al. [4] emphasize that while routine occupations have been replaced between 1999 and 2010, product demand has also increased as a result of routine-replacing technological change leading to a net employment growth. Also focusing on routine-task intensity, Lewandowski et al. [3] focus on cross-country differences in the level of routineness in occupations. They find lower levels of routineness in high-skill occupations in countries with a higher GDP per capita. They also find correlations between computer use, higher education and higher literacy skills in a given country with routine task intensity.

While much of the automation literature relies on past employment data, the rapid progress on robotics, artificial intelligence (AI) and automation technologies has also motivated predicting automation developments in the near future [10, 11]. The importance of this exercise is bellied in Lordan [12] and Lordan and Neumark [13] who suggest that new jobs are now being automated; particularly jobs traditionally at the bottom of the occupation distribution. Further, advances in AI and in particular machine learning will likely affect at least some tasks in most occupations and will hence also disrupt jobs at the top of the income distribution [14].

Concretely, Webb [8] studies the impact of automation on occupational tasks and matches information on job tasks to patents issued for robots, software and AI to identify which tasks can be automated by different technologies to derive an exposure to automation score. He uses Google patents data, the O*NET database of occupations and tasks and US census data. O*NET is a database of occupations and tasks published by the US Department of Labor that provides detailed descriptions of a large number of occupations and has been used frequently in the literature studying the impact of automation and technical innovation on employment [5, 6, 8, 15]. He first analyzes the impact of his ‘exposure to automation score on employment using historical data on robots and software patents and job descriptions and then repeats this exercise using patents on AI to predict future employment effects. AI is studied with respect to future developments as it is a relatively new phenomenon as compared to software and robotic innovations. While innovation on robots and software has mainly affected low skill and low wage occupations in the past, he finds that AI is increasingly predicted to disrupt high-skill occupations. Building on this work, Tolan et al. [16] link research intensity in AI to abilities required for specific job tasks using European survey data, O*NET data and AI benchmarking platforms. They find that jobs that were originally classified as non-automatable are increasingly affected by automation such as medical doctors. They find that abilities particularly affected by automation are abilities for idea creation while people abilities are less affected.

Lordan and Josten’s [10] study is also forward-looking and takes the occupations classified by Autor and Dorn (2013) as given while reclassifying the remaining occupations as automatable depending on the number of patents recently available for each specific occupation thereby also predicting which jobs will be automated in the near future. They try to capture the most recent wave of automation by using patent developments in artificial intelligence, robots and automation more broadly as a proxy for technology that will be on the market shortly. If for any given occupation the authors find a large number of patents and find that successful patent pilots have been covered by the media, this occupation is classified as being on track to become automatable. A full list of the occupations classified as automatable by Lordan and Josten [10] can be found in S1 Table. This study builds on and uses the classification by Lordan and Josten [10] to analyze which job attributes and requirements predict the likelihood that a job is reclassified as automatable under their new definition. It thereby speaks to the literature on the automatability of tasks, skills and abilities.

In particular we use the automatability indicator by Lordan and Josten [10] matched with employment data from the European Labor Force Survey (EU-LFS) and with data on the skills and abilities required on the job from O*NET. The EU-LFS covers employment statistics of households from EU member countries, Switzerland, Sweden and the UK quarterly. We then regress the automatability indicator on the skills and abilities respectively to analyze which skills and which abilities required in different occupations are susceptible to automation. This is then further linked to work by Lordan and Pischke [17] who capture the ‘people’, ‘brains’ and ‘brawn’ content of occupations with different risks of being automated; i.e. the extent to which an occupation involves people interaction, cognitive thinking skills or physicality respectively.

Identifying future job requirements by occupations is crucial to be able to quantify changes in demand for skills and abilities resulting from automation [18]. First, this information is relevant to policymakers and companies interested in the future of work. In particular, it informs on the skills and abilities required of workers in the near future as well as the activities they will likely be engaged in. Second, these findings further help conversations surrounding the re-organization of education and other development activities to ensure the stock and flow of the relevant skills for the 4^th^ industrial revolution. The returns to education are constantly increasing with the rise in technological progress with specific skills such as digital and non-cognitive skills becoming particularly important [19]. Third, this information also helps to gain a more nuanced understanding of the exact aspects of the occupations at risk of automation and hence addresses criticism by Arntz et al. [20], among others, who find that focusing on the automatability of occupations rather than job tasks overstates the risk of automation and omits important aspects of the automation developments.

## 2. Data

### Job level abilities and skills proxies

To capture the abilities and skills required by occupations at the three-digit occupation level we draw on O*NET version 15. O*NET is an occupational database by the US Department of Labor that narrowly defines occupations with respect to the tasks and activities and the skills and abilities required on the job. This database has been used frequently in the automation literature both in the US and the UK [2, 3, 5, 16]. The difference in task content of occupations in the US versus the UK has been shown to be small, further justifying using this resource to classify occupation attributes using UK data [3, 21].

Specifically, O*NET offers 80 distinct items in the abilities classification. The first column of Table 2 lists each of these items and column (2) provides a brief description of the item. The third column of Table 2 documents the secondary category a specific ability is in, while column (4) provides an overall category. In addition to abilities, O*NET offers 40 distinct items in the skills classification. Again the first column of Table 3 lists each of these items and column (2) provides a brief description each item. The third column documents the secondary category a specific ability is in, while column (4) provides an overall category. Given the large number of abilities (80) and skills (40) items, the secondary and overall category in columns (3) and (4) from Tables 2 and 3 will be utilized respectively for the interpretation of the analysis.

### EU LFS and Lordan and Josten

Our analysis relies on data from Lordan and Josten [10] who match data from the European Labour Force Survey (EU-LFS) between 2013–2016 to their automation classification. The EU-LFS is conducted across all Member States of the European Union, Iceland, Norway, Switzerland and the United Kingdom and consists of quarterly collected household data on employment. We include all countries that have data in the years we analyze, in addition to 3-digit occupation codes. A full list of the countries we include can be found in Table 1.

**Table 1:** Lordan and Josten (2020) shares of employment in 2013–2016 by country.

	Recently Automatable
Austria	0.516
Belgium	0.443
Croatia	0.565
Cyprus	0.512
Czech Republic	0.516
Denmark	0.370
Estonia	0.458
Finland	0.207
France	0.478
Germany	0.474
Greece	0.574
Hungary	0.544
Iceland	0.374
Ireland	0.481
Italy	0.508
Latvia	0.525
Lithuania	0.527
Luxembourg	0.442
Netherlands	0.374
Norway	0.363
Portugal	0.485
Spain	0.474
Sweden	0.378
Slovakia	0.513
UK	0.405

Lordan and Josten [10] match their definition of recently automatable jobs based on patents at the 3-digit code occupation level to the EU-LFS using crosswalks provided by Lordan and Pischke [17]. To derive the automation classification, Lordan and Josten [10] revise 216 occupations that have been classified as non-automatable by Autor and Dorn [6] and search for the occupation name together with either the term “robot”, “automation”, or “artificial intelligence” in Google patents and then also in Google News. Depending on the number of patents and/or news articles, an occupation is then classified as either fully automatable, polarized automatable (i.e. partially automatable) or non-automatable.

Table 1 shows the shares of automatable employment (i.e. polarized and fully automatable) by EU-LFS country. We first note that the Lordan and Josten [10] classifications suggest that a large share of jobs in every country is recently automatable. Specifically, Finland is the country that has the lowest share of jobs that are classified as recently automatable (approximately 21%). For the remaining Scandinavian countries (Norway, Sweden and Denmark), Table 1 suggests that about 37% of the jobs are automatable, similar to the shares for the Netherlands and Iceland. In contrast, for Croatia, Czech Republic, Italy and Latvia more than 50% of the jobs are recently automatable. Overall, Table 1 highlights that there is large variation in the share of occupations that can be automated across EU-LFS countries.

## 3. Methodology

The main analysis in this work relies on EU-LFS data. Our main analysis relies on an indicator variable that is equal to 1 if an occupation is newly automatable under the Lordan and Josten [10] classification and 0 otherwise. Jobs that are denoted as automatable by Author and Dorn [5] are excluded from the analysis given that these jobs were classified as automatable based on O*NET occupation attributes (i.e. routine, manual and abstract tasks), so they mechanically relate to the O*NET attributes. In addition, this exclusion allows us to clarify the differences in skills and abilities between non-automatable jobs and newly automatable jobs. If these jobs were included this delineation would not be possible.

We proceed by regressing the indicator variable on each set of job attributes and skills respectively as provided by O*NET. We control for differences across country with a set of country fixed effects and for differences across time with a set of year fixed effects. We have two main sets of regressions. The first set regresses the automation indicator variable on the 80 ability domains and the second regresses it on the 40 skills domains. We apply Lasso regression analysis, a shrinkage and a variable selection method for linear regression models. This approach is chosen as we wish to reduce the dimensionality of the abilities and skills variables under consideration. The goal of a Lasso regression is to obtain the subset of predictors that minimizes prediction error for a quantitative response variable. The Lasso does this by imposing a constraint on the model parameters that causes regression coefficients for some variables to shrink toward zero. Variables with a regression coefficient equal to zero after the shrinkage process are excluded from the model. That is, these variables do not explain variation in the propensity for a job to be recently automatable and will be shown with a value of zero in the results tables. The remaining variables with a positive sign are those that describe the core skills and abilities that are most likely to become redundant because of the most recent wave of automation. In contrast, the remaining variables with a negative sign describe the core skills and abilities that are most likely to become more valuable. All non-zero variables are significant at the 1% significance level.

When estimating the Lasso for the abilities attributes we include 60 abilities attributes, in addition to country and year fixed effects. Country fixed effects control for location specific factors that are time invariant. Given the small time window we do not expect that time varying factors will distort our results.

## 4. Results

### Abilities

The results from the pooled country analysis for abilities are documented in Table 2. We first note that the regression considering abilities explains 97% of the variation in the Lordan and Josten [10] automation indicator. That is, we can explain almost all of the variation in the indicator variable with these measures of ability. Second, if we take column (3) and column (4) there is no single secondary or overall ability category that persistently has negative or positive signs. Rather, within each of these categories there are abilities that are becoming less important and others that are becoming more important. For example, arm-hand steadiness is a fine manipulative psychomotor ability (e.g. the ability to keep your hand and arm steady while moving your arm) that is highly unlikely to be automated given the estimates. In contrast, finger dexterity which is also a fine manipulative psychomotor ability (i.e. the ability to make precisely coordinated movements of the fingers of one or both hands to grasp, manipulate, or assemble very small objects) is likely to be automated. Similarly, fluency of ideas is an idea generation and reasoning ability which relates to the ability to come up with a number of ideas about a topic (i.e. the number of ideas is important, not their quality, correctness, or creativity) which is highly unlikely to be automated. In contrast, problem sensitivity (i.e. recognizing that there is a problem but not solving the problem) in the same category is highly likely to be automated.

**Table 2:** Abilities estimates.

(1)	(2)	(3)	(4)	(5)
ONET Item	Description	Secondary category	Overall category	Coefficient
Arm-Hand Steadiness	The ability to keep your hand and arm steady while moving your arm or while holding your arm and hand in one position.	Fine manipulative abilities	Psychomotor abilities	-3.587
Auditory Attention	The ability to focus on a single source of sound in the presence of other distracting sounds.	Auditory and speech abilities	Sensory Abilities	0.000
Category Flexibility	The ability to generate or use different sets of rules for combining or grouping things in different ways.	Idea Generation and Reasoning Abilities	Cognitive Abilities	-2.381
Control Precision	The ability to quickly and repeatedly adjust the controls of a machine or a vehicle to exact positions.	Control Movement Abilities	Psychomotor Abilities	1.663
Deductive Reasoning	The ability to apply general rules to specific problems to produce answers that make sense.	Idea Generation and Reasoning Abilities	Cognitive Abilities	-4.904
Depth Perception	The ability to judge which of several objects is closer or farther away from you, or to judge the distance between you and an object.	Visual Abilities	Sensory Abilities	0.558
Dynamic Flexibility	The ability to quickly and repeatedly bend, stretch, twist, or reach out with your body, arms, and/or legs.	Flexibility, Balance and Coordination	Physical Abilities	2.784
Dynamic Strength	The ability to exert muscle force repeatedly or continuously over time. This involves muscular endurance and resistance to muscle fatigue.	Physical Strength	Physical Abilities	-0.256
{2,3Explosive Strength	The ability to use short bursts of muscle force to propel oneself (as in jumping or sprinting), or to throw an object.	Physical Strength	Physical Abilities	0.555
Extent Flexibility	The ability to bend, stretch, twist, or reach with your body, arms, and/or legs.	Flexibility, balance and coordination	Physical Abilities	-2.347
Far Vision	The ability to see details at a distance.	Visual Abilities	Sensory Abilities	-2.219
Finger Dexterity	The ability to make precisely coordinated movements of the fingers of one or both hands to grasp, manipulate, or assemble very small objects.	Fine manipulative abilities	Psychomotor abilities	2.169
Flexibility of Closure	The ability to identify or detect a known pattern (a figure, object, word, or sound) that is hidden in other distracting material.	Perceptual Abilities	Cognitive abilities	0.000
Fluency of ideas	The ability to come up with a number of ideas about a topic (the number of ideas is important, not their quality, correctness, or creativity).	Ideas generation and reasoning abilities	Cognitive abilities	-2.830
Glare sensitivity	The ability to see objects in the presence of glare or bright lighting.	Visual abilities	Sensory abilities	-3.635
Gross Body Co-ordination	The ability to coordinate the movement of your arms, legs, and torso together when the whole body is in motion.	Flexibility, Balance and Coordination	Physical Abilities	0.266
Gross Body Equilibrium	The ability to keep or regain your body balance or stay upright when in an unstable position.	Flexibility, Balance and Coordination	Physical Abilities	0.000
Hearing Sensitivity	The ability to detect or tell the differences between sounds that vary in pitch and loudness.	Auditory and Speech abilities	Sensory Abilities	-1.139
Inductive Reasoning	The ability to combine pieces of information to form general rules or conclusions (includes finding a relationship among seemingly unrelated events).	Idea Generation and Reasoning Abilities	Cognitive Abilities	0.000
Information Ordering	The ability to arrange things or actions in a certain order or pattern according to a specific rule or set of rules (e.g., patterns of numbers, letters, words, pictures, mathematical operations).	Idea Generation and Reasoning Abilities	Cognitive Abilities	0.000
Manual Dexterity	The ability to quickly move your hand, your hand together with your arm, or your two hands to grasp, manipulate, or assemble objects.	Fine Manipulative Abilities	Psychomotor Abilities	0.000
Math Reasoning	The ability to choose the right mathematical methods or formulas to solve a problem.	Quantitative Abilities	Cognitive Abilities	1.253
Memorization	Abilities related to the recall of available information.	Quantitative Abilities	Cognitive Abilities	0.000
Multi Limb Co-ordination	The ability to coordinate two or more limbs (for example, two arms, two legs, or one leg and one arm) while sitting, standing, or lying down. It does not involve performing the activities while the whole body is in motion.	Control movement abilities	Psychomotor Abilities	1.604
Near Vision	The ability to see details at close range (within a few feet of the observer).	Near Vision	Visual Abilities	0.000
Night Vision	The ability to see under low light conditions.	Near Vision	Visual Abilities	3.025
Number Facility	The ability to add, subtract, multiply, or divide quickly and correctly.	Quantitative Abilities	Cognitive Abilities	-1.817
Oral Comprehension	The ability to listen to and understand information and ideas presented through spoken words and sentences.	Verbal Abilities	Cognitive Abilities	0.000
Oral Expression	The ability to communicate information and ideas in speaking so others will understand.	Verbal Abilities	Cognitive Abilities	4.378
Originality	The ability to come up with unusual or clever ideas about a given topic or situation, or to develop creative ways to solve a problem.	Idea generation and Reasoning Abilities	Cognitive Abilities	0.000
Perceptual Speed	The ability to quickly and accurately compare similarities and differences among sets of letters, numbers, objects, pictures, or patterns. The things to be compared may be presented at the same time or one after the other. This ability also includes comparing a presented object with a remembered object.	Perceptual Abilities	Cognitive Abilities	-4.204
Perceptual Vision	The ability to see objects or movement of objects to one’s side when the eyes are looking ahead.	Visual Abilities	Sensory Abilities	0.713
Problem Sensitivity	The ability to tell when something is wrong or is likely to go wrong. It does not involve solving the problem, only recognizing there is a problem.	Idea generation and Reasoning Abilities	Cognitive Abilities	2.190
Rate Control	The ability to time your movements or the movement of a piece of equipment in anticipation of changes in the speed and/or direction of a moving object or scene.	Control Movement Abilities	Psychomotor Abilities	3.112
Reaction Time	The ability to quickly respond (with the hand, finger, or foot) to a signal (sound, light, picture) when it appears.	Reaction Time and Speed Abilities	Psychomotor Abilities	2.509
Response Orientation	The ability to choose quickly between two or more movements in response to two or more different signals (lights, sounds, pictures). It includes the speed with which the correct response is started with the hand, foot, or other body part.	Control Movement Abilities	Psychomotor Abilities	-4.984
Selective Attention	The ability to concentrate on a task over a period of time without being distracted.	Attentiveness	Cognitive Abilities	-0.963
Sound Localisation	The ability to tell the direction from which a sound originated.	Auditory and Speech Abilities	Sensory Abilities	-1.685
Spatial Orientation	The ability to know your location in relation to the environment or to know where other objects are in relation to you.	Spatial Abilities	Cognitive Abilities	1.051
Speech Clarity	The ability to speak clearly so others can understand you.	Auditory and Speech Abilities	Sensory Abilities	0.000
Speech Recognition	The ability to identify and understand the speech of another person.	Auditory and Speech Abilities	Sensory Abilities	-2.099
Speed of Closure	The ability to quickly make sense of, combine, and organize information into meaningful patterns.	Perceptual Abilities	Cognitive Abilities	5.164
Speed of Limb Movement	The ability to quickly move the arms and legs.	Reaction Time and Speed Abilities	Psychomotor Abilities	-1.118
Stamina	The ability to exert yourself physically over long periods of time without getting winded or out of breath.	Endurance	Physical Abilities	0.557
Static Strength	The ability to exert maximum muscle force to lift, push, pull, or carry objects.	Physical Strength Abilities	Physical Abilities	0.679
Time Sharing	The ability to shift back and forth between two or more activities or sources of information (such as speech, sounds, touch, or other sources).	Attentiveness	Cognitive Abilities	2.796
Trunk Strength	The ability to use your abdominal and lower back muscles to support part of the body repeatedly or continuously over time without ’giving out’ or fatiguing.	Physical Strength Abilities	Physical Abilities	0.000
Visual Color Discrimination	The ability to match or detect differences between colors, including shades of color and brightness.	Visual Abilities	Sensory Abilities	4.322
Visualisation	The ability to imagine how something will look after it is moved around or when its parts are moved or rearranged.	Spatial Abilities	Cognitive Abilities	-0.752
Wrist-Finger Speed	The ability to make fast, simple, repeated movements of the fingers, hands, and wrists.	Reaction Time and Speed Abilities	Psychomotor Abilities	-1.251
Written Comprehension	The ability to read and understand information and ideas presented in writing.	Verbal Abilities	Cognitive Abilities	0.000
Written Expression	The ability to communicate information and ideas in writing so others will understand.	Verbal Abilities	Cognitive Abilities	-0.295
R Squared = 0.97				2701297

Although it may seem counter-intuitive that within the same ability category there are pairs of abilities that are both automatable and non-automatable, the description of each item (see column (2) Table 2) highlights a logical theme. In general, an ability is automatable if it entails an underlying action that is repeatable or follows some logic. For example, control precision relies on adjusting controls quickly and repeatedly. The repetitive nature of this ability implies it is easily codifiable and machines have a comparative advantage in speed of execution. In contrast, speech recognition, which involves understanding spoken language, is unlikely to be automated. This finding is intuitive as it is difficult to predict what someone will say unless it entails common conversations such as those in telephone customer care. For example, customer care in banking now frequently utilize artificial intelligence to direct calls or provide bank balances but it is human operators that deal with complaints and other issues.

The overall conclusion from studying Table 2 is that jobs that are high on ‘brains’ (i.e. involve abstract thinking) are far less likely to be automated. In this case, ‘brains’ is short-hand for thinking and can involve reacting to other individuals (e.g. in caring or teaching professions), performing a service (e.g. as mechanic or fine dining waiter) or engaging in agile or creative thinking (e.g. in a leadership or knowledge worker role). Occupations that are predicted to be automated are low on ‘brains’ and high in routine contents. The abilities analysis is hence largely in line with the work by Author and Dorn [6] who predict that jobs that involve routine tasks will be automated. However, given that in this analysis we only focus on the occupations classified as non-automatable by Author and Dorn [6] it further reiterates that automation has continued to make progress automating jobs that are high in routine content.

### Skills

The results from the pooled country analysis for skills are documented in Table 3. This regression explains 84% of the variation in the Lordan and Josten [10] automation indicator. Analyzing columns (3) and (4) of Table 3, the results are consistent with the analysis of abilities in that there is again no secondary or overall skills category that has a persistent negative or positive effect on the automation indicator. In addition and also consistent with the abilities analysis, there is a pattern which suggests that ‘brains’ (i.e. thinking and reacting) is becoming more important while on the job as compared to routine work.

**Table 3:** Skills estimates.

(1)	(2)	(3)	(4)	(5)
ONET Item	Description	Secondary category	Overall category	Coefficient
Active Learning	Understanding the implications of new information for both current and future problem-solving and decision-making.	Process	Basic Skills	1.630
Active Listening	Giving full attention to what other people are saying, taking time to understand the points being made, asking questions as appropriate, and not interrupting at inappropriate times.	Content	Basic Skills	0.608
Complex Problem Solving	Identifying complex problems and reviewing related information to develop and evaluate options and implement solutions.	Complex Problem Solving	Cross Functional Skills	-0.580
Coordination	Adjusting actions in relation to others’ actions.	Social Skills	Cross Functional Skills	-6.025
Critical Thinking	Using logic and reasoning to identify the strengths and weaknesses of alternative solutions, conclusions or approaches to problems.	Process	Basic Skills	-3.233
Equipment Maintenance	Performing routine maintenance on equipment and determining when and what kind of maintenance is needed.	Technical Skills	Cross Functional Skills	-0.504
Equipment Selection	Determining the kind of tools and equipment needed to do a job.	Technical Skills	Cross Functional Skills	0.000
Installation	Installing equipment, machines, wiring, or programs to meet specifications.	Technical Skills	Cross Functional Skills	0.540
Instructing	Teaching others how to do something.	Social Skills	Cross Functional Skills	-2.752
Judgement and Decision Making	Considering the relative costs and benefits of potential actions to choose the most appropriate one.	Systems Skills	Cross Functional Skills	2.775
Learning Strategies	Selecting and using training/instructional methods and procedures appropriate for the situation when learning or teaching new things.	Systems Skills	Cross Functional Skills	0.000
Management of Financial Resources	Determining how money will be spent to get the work done, and accounting for these expenditures.	Resource Management Skills	Cross functional Skills	0.000
Management of Material Resources	Obtaining and seeing to the appropriate use of equipment, facilities, and materials needed to do certain work.	Resource Management Skills	Cross functional Skills	-1.262
Management of Personnel Resources	Motivating, developing, and directing people as they work, identifying the best people for the job.	Resource Management Skills	Cross functional Skills	2.264
Mathematics	Using mathematics to solve problems.	Content	Basic Skills	0.000
Monitoring	Monitoring/Assessing performance of yourself, other individuals, or organizations to make improvements or take corrective action.	Process	Basic Skills	-4.676
Negotiation	Bringing others together and trying to reconcile differences.	Social Skills	Cross functional Skills	1.967
Operation Monitoring	Watching gauges, dials, or other indicators to make sure a machine is working properly.	Technical Skills	Cross Functional Skills	1.831
Operation and Control	Controlling operations of equipment or systems.	Technical Skills	Cross Functional Skills	0.000
Operations Analysis	Analyzing needs and product requirements to create a design.	Technical Skills	Cross Functional Skills	-1.091
Persuasion	Persuading others to change their minds or behavior.	Social Skills	Cross functional Skills	-0.827
Programming	Writing computer programs for various purposes.	Technical Skills	Cross functional Skills	0.000
Quality Control Analysis	Conducting tests and inspections of products, services, or processes to evaluate quality or performance.	Technical Skills	Cross functional Skills	0.000
Reading Comprehension	Understanding written sentences and paragraphs in work related documents.	Content	Basic Skills	-2.474
Repairing	Repairing machines or systems using the needed tools.	Technical Skills	Cross Functional Skills	-0.935
Science	Using scientific rules and methods to solve problems.	Content	Basic Skills	1.044
Service Orientation	Actively looking for ways to help people.	Social Skills	Cross Functional Skills	-0.854
Social Perceptiveness	Being aware of others’ reactions and understanding why they react as they do.	Social Skills	Cross Functional Skills	0.000
Speaking	Talking to others to convey information effectively.	Content	Basic Skills	1.235
System Analysis	Determining how a system should work and how changes in conditions, operations, and the environment will affect outcomes.	Systems Skills	Cross Functional Skills	-2.530
System Evaluation	Identifying measures or indicators of system performance and the actions needed to improve or correct performance, relative to the goals of the system.	Systems Skills	Cross Functional Skills	4.365
Technology Design	Generating or adapting equipment and technology to serve user needs.	Technical Skills	Cross Functional Skills	-2.203
Time Management	Managing one’s own time and the time of others.	Resource Management Skills	Cross Functional Skills	4.214
Troubleshooting	Determining causes of operating errors and deciding what to do about it.	Technical Skills	Cross Functional Skills	0.000
Writing	Communicating effectively in writing as appropriate for the needs of the audience.	Content	Basic Skills	1.686
R squared = 0.84			N =	2701297

For example, within the overall category of basic skills (see column 4), the O*NET item active learning is a skill that is more likely to be automated. This is consistent with machines being able to process a large amount of information quickly. However, the O*NET items critical thinking and monitoring of performance, which essentially involve using information that is available to pass judgement and make decisions, are less likely to be automated. Within cross functional skills, skills that center around identifying potential problems, setting rules and gathering information are most likely to be automated (for example the O*NET items of time management, operation monitoring, system evaluation and management of human resources). In contrast, those skills that require manual actions (e.g. equipment maintenance), communicating information (for example instructing), leadership (e.g. persuasion) and knowledge work (e.g. systems analysis and technology design) are less likely to be automated.

A second notable pattern emerges if we reconsider Table 3. In general, the skills that require active interactions with people (i.e. implying that there is at least a two-way dialogue where an employee is reacting to the other person(s)) are not automatable. In essence, these jobs are an interaction between ‘brains’ and ‘people’ skills. In Table 3, these include coordination, instructing, monitoring and persuasion. This observation seems to suggest that jobs that involve ‘people’ interacted with ‘brains’ skills are also less likely to be automated.

### Conclusion from the skills and abilities analysis

From the estimates documented in Tables 2 and 3 we make the following three conclusions:

1. We can explain almost all of the variation in the jobs that are newly defined as automatable by Lordan and Josten [10] using the O*NET items of abilities or skills.
2. Jobs that require ‘brains’ (i.e. abstract and non-linear thinking) are far less likely to be automated as compared to jobs which require linear and codifiable thinking skills and abilities. At the top of the income distribution, jobs that require non-linear thinking may need critical thinking, decision-making and creativity. Elsewhere in the income distribution these jobs require skills that have been traditionally delivered in apprenticeships, from mechanics and carpenters to florists and hairdressers.
3. Jobs that require ‘people’ engagement interacted with ‘brains’ are also less likely to be automated. These jobs include management across all levels, coordinators of all types, teachers, carer and medical practitioners (including nursing). The skills and abilities that are required for these jobs include soft skills. The value of these skills in terms of adult outcomes has become a topic of recent writings in economics (for example Heckman and Kautz (2013) [22]; Kautz et al., (2014) [23] and Lordan and McGuire (2019) [24]) and has been recently noted specifically as skills that will be needed in the advent of the Fourth Industrial Revolution [12, 25].

### Activities

To summarize further the conclusions described in conclusions 2. and 3. from the skills and abilities analysis (i.e. that jobs which require ‘brains’ and ‘people’ interacted with ‘brains’ are those that are the least likely to be automated) we consider a third analysis on work activities and context of a person’s occupation. This third analysis is a replication of Table 3 in Lordan and Josten [10]. We later go further than Lordan and Josten [10] and present these estimates separately for each country in our dataset to allow for cross country comparisons.

To replicate their work we first follow Lordan and Pischke [17] and create three factors that represent the ‘people’ ‘brains’ and ‘brawn’ content in each three-digit code occupation. That is:

1. ‘People’ is a variable, which distils the information from many domains in activities and context that relate to having interactions with people on a day-to-day basis.
2. ‘Brains’ is a variable which distils the information from many domains in activities and context that relate to abstract thinking.
3. ‘Brawn’ is a variable which distils the information from many domains in activities and context that relate to interacting physically with objects, including making them on a daily basis.

We then regress our dummy representing whether a job is automatable on the ‘people’, ‘brains’ and ‘brawn’ variables (using ordinary least squares (OLS)). Consistent with Lordan and Josten [10]) we also interact the three variables with each other, implying we also include in our regression people*brains, people*brawn and brains*brawn. We again control for fixed country differences and yearly differences in the regression. The results from this analysis are documented in Table 4.

**Table 4:** ‘People’, ‘brains’ and ‘brawn’ estimates.

Variable	Marginal effect
People	0.009***
	(0.000)
Brains	-0.070***
	(0.000)
Brawn	0.032***
	(0.000)
People * Brains	-0.002***
	(0.000)
People* Brawn	0.003***
	(0.000)
Brains* Brawn	0.000***
	(0.000)
N	2,698,151
R squared	42%

Notes *, **, *** denotes significance at the 10%, 5% and 1% levels respectively.

Turning to Table 4, we can explain 42% of the variation in our automatable employment indicator with the three latent factors. The size and significance of the negative coefficient on the ‘brain’ factor strongly implies that jobs which require thinking are those that are safe from the most recent wave of automation. In addition, the interaction between the ‘people’ and ‘brains’ factors is negative and significant. This highlights that jobs which require thinking and social interactions are safer in the most recent wave of automation as compared to those that simply involve people interactions (as evidenced by the positive and significant coefficient on the ‘people’ factor). We note that the estimates in Table 4 imply that jobs that are high on ‘brawn’ content are those that are most likely to be automated in the most recent wave of automation. These jobs include making objects and physically lifting items. Overall, these conclusions align well with those that came from the abilities and skills analyses in Tables 2 and 3 respectively as they highlight the importance of abstract thinking and the combination of abstract thinking and people skills.

### Country-level analysis

The S1 Appendix contains the estimates from Table 2 through 4 by country including all EU-LFS countries as depicted in Table 1. The differences across these estimates is driven only by differences in the occupation distribution for each country, while the classification of whether a job is recently automatable is fixed across time and country. We note that the separate analyses of skills and abilities for each country allows us to draw similar conclusions to the pooled country estimates in Tables 2 and 3. That is, the abilities and skills that are becoming more important relate to the ability and skill to use ‘brains’ for abstract, strategic and creative thinking and the ability and skill to use ‘brains’ interacted with ‘people’.

The ‘people’, ‘brains’ and ‘brawn’ categories allow us to summarize the differences and commonalities across countries most succinctly. These are:

1. For all countries, jobs that are high on ‘brains’ are least likely to be automated in the most recent wave of automation.
2. For many countries the ‘people’ coefficient is also negative and significant, implying that jobs that are ‘people’ facing are relatively safe from automation, regardless of their interaction with ‘brains’. These countries are: Austria, Belgium, Cyprus, Czech Republic, Denmark, Germany, Estonia, Spain, France, Ireland, the Netherlands, Norway, Sweden.
3. For almost all countries there is a negative and significant relationship between the potential for automation and the ‘people’ and ‘brains’ interaction. The exceptions are Cyprus, Estonia, Ireland, Norway, Sweden and the UK. We note that for Estonia, Ireland, Norway and Sweden both the ‘people’ and ‘brains’ effects are independently negative and significant, highlighting that ‘people’ jobs in general are unlikely to disappear significantly. For Cyprus and the UK, the estimates suggest that ‘brains’ are the most important skills and abilities to develop given the current distribution of jobs.
4. In general, the brawn effect is positive and significant, implying that jobs that are high on ‘brawn’ content are at risk from automation. The exception of countries where this effect is negative are Cyprus, Czech Republic and Estonia.
5. The interaction effect between ‘people’ and ‘brawn’ is in general positive, significant but small in magnitude for just over half the countries in our study. In contrast, it is negative and significant but small in magnitude for: Finland, France, Hungary, Ireland, Iceland, Latvia, Norway, Sweden, Slovakia and the UK. The difference seems to be driven by a relatively large number of jobs in these countries in the low end of the income distribution that require physical lifting and people (for example, cleaning and caring).
6. The interaction effect between ‘brains’ and ‘brawn’ is in general negative, significant but small in magnitude. There are only three exceptions. These are: Cyprus, Czech Republic and Ireland.

## 5. Conclusion

This study identifies the job attributes which predict the likelihood that a job is recently automatable. In particular, it looks at the i) abilities and ii) skills required on the job and how they link to automatability. A third analysis (iii) considers the ‘people’, ‘brains’ and ‘brawn’ content of an occupation, i.e. the extent to which an occupation involves people interactions, abstract thinking and physicality respectively. The three analyses are also done on a country level to see how the impact of automatability on the labour market differs across countries.

Overall, we find that skills and abilities which relate to non-linear abstract thinking, which we term ‘brains’, are those that are the safest from automation. We also find that jobs that require ‘people’ engagement interacted with ‘brains’ are also less likely to be automated. The skills and abilities that are required for these jobs include soft skills. Finally, we find that jobs that require physicality (e.g. creating objects manually) are most likely to be automated unless they involve interaction with ‘brains’ and/or ‘people’.

These findings are in line with the literature on the growing importance of cognitive and social skills for the future of work. In particular, Deming [25] finds that the interaction between cognitive and social skills has seen greater wage and employment growth, which is comparable to our finding of the importance of ‘brains’ alongside ‘people’ skills and abilities. It also matches findings of studies that link skills endowments or demand for skills to labour market outcomes and find that social and cognitive skills are increasingly rewarded and that there is a complementarity across those two dimension [26, 27].

Information and knowledge on future job requirements by occupations and by country is essential when trying to predict the demand for skills and abilities and activities going forward. It is important knowledge for policymakers and companies who can adapt policies and organizational settings regarding the future of work accordingly and ensure that individuals are prepared for current developments and what is yet to come. In particular, it informs conversations surrounding the re-organization of education and other development activities to ensure that the stock and flow of skills are ready for the Fourth Industrial Revolution. The returns to education are constantly increasing with the rise in technological progress with specific skills such as digital and non-cognitive skills becoming particularly important [19]. And this information also helps to gain a more nuanced understanding of the exact aspects of the occupations at risk of automation rather than just predicting automation overall and hence extends previous work. While we summarize our findings at the ‘people’, ‘brains’ and ‘brawn’ level, we still show and have briefly discussed the results by each O*NET abilities and skills item, which is informative to the reader interested in specific aspects of occupations and their automatability.

The differences in effects found at the country level likely reflect the fact that the structure of jobs and skills within country differ, coupled with each country being on a different trajectory with respect to automation. In addition, the policies that can protect jobs from automation also differ within country. A better understanding of such within country policies, coupled with their interaction with the labour market is an area for future research.

## Supporting information

S1 Table Occupations classified by Josten and Lordan (2020) as automatable. (DOCX)

S1 Appendix Individual country analysis. (DOCX)
