# Supplementary material for: Automation and the changing nature of work
Source: PLoS One. 2022 May 5;17(5):e0266326. doi: 10.1371/journal.pone.0266326 (PMC9071144; doi:10.1371/journal.pone.0266326)
Supplement: S1 Table — (DOCX) [file pone.0266326.s001.docx]

**S1 Table: Occupations Classified by Josten and Lordan (2020) as automatable**

| **Occupation** | **Lordan and Josten** | |
| --- | --- | --- |
| Chief executives, public administrators, and legislators | Non- Automatable | |
| Financial managers | Automatable | |
| Human resources and labour relations managers | Automatable | |
| Managers and specialists in marketing, advert., PR | Automatable | |
| Managers in education and related fields | Non- Automatable | |
| Managers of medicine and health occupations | Non- Automatable | |
| Managers of properties and real estate | Non- Automatable | |
| Funeral directors | Fully Automatable | |
| Managers and administrators, n.e.c. | Non- Automatable | |
| Accountants and auditors | Fully Automatable | |
| Insurance underwriters | Fully Automatable | |
| Other financial specialists | Fully Automatable | |
| Management analysts | Non- Automatable | |
| Personnel, HR, training, and labour rel. specialists | Automatable | |
| Purchasing agents and buyers of farm products | Fully Automatable | |
| Buyers, wholesale and retail trade | Non- Automatable | |
| Purchasing managers, agents, and buyers, n.e.c. | Non- Automatable | |
| Business and promotion agents | Non- Automatable | |
| Construction inspectors | Non- Automatable | |
| Inspectors and compliance officers, outside | Non- Automatable | |
| Management support occupations | Automatable | |
| Architects | Non- Automatable | |
| Aerospace engineers | Non- Automatable | |
| Metallurgical and materials engineers | Non- Automatable | |
| Petroleum, mining, and geological engineers | Non- Automatable | |
| Chemical engineers | Non- Automatable | |
| Civil engineers | Non- Automatable | |
| Electrical engineers | Non- Automatable | |
| Industrial engineers | Non- Automatable | |
| Mechanical engineers | Non- Automatable | |
| Engineers and other professionals, n.e.c. | Non- Automatable | |
| Computer systems analysts and computer scientists | Non- Automatable | |
| Operations and systems researchers and analysts | Non- Automatable | |
| Actuaries | Fully Automatable | |
| Mathematicians and statisticians | Non- Automatable | |
| Physicists and astronomists | Non- Automatable | |
| Chemists | Non- Automatable | |
| Atmospheric and space scientists | Non- Automatable | |
| Geologists | Non- Automatable | |
| Physical scientists, n.e.c. | Fully Automatable | |
| Agricultural and food scientists | Non- Automatable | |
| Biological scientists | Non- Automatable | |
| Foresters and conservation scientists | Non- Automatable | |
| Medical scientists | Non- Automatable | |
| Physicians | Non- Automatable | |
| Dentists | Non- Automatable | |
| Veterinarians | Non- Automatable | |
| Optometrists | Automatable | |
| Podiatrists | Non- Automatable | |
| Other health and therapy occupations | Non- Automatable | |
| Registered nurses | Non- Automatable | |
| Pharmacists | Automatable | |
| Dieticians and nutritionists | Fully Automatable | |
| Respiratory therapists | Non- Automatable | |
| Occupational therapists | Non- Automatable | |
| Physical therapists | Non- Automatable | |
| Speech therapists | Automatable | |
| Therapists, n.e.c. | Non- Automatable | |
| Physicians' assistants | Automatable | |
| Subject instructors, college | Non- Automatable | |
| Kindergarten and earlier school teachers | Non- Automatable | |
| Primary school teachers | Non- Automatable | |
| Secondary school teachers | Non- Automatable | |
| Special education teachers | Non- Automatable | |
| Teachers, n.e.c. | Non- Automatable | |
| Vocational and educational counselors | Non- Automatable | |
| Librarians | Automatable | |
| Archivists and curators | Non- Automatable | |
| Economists, market and survey researchers | Non- Automatable | |
| Psychologists | Non- Automatable | |
| Social scientists and sociologists, n.e.c. | Non- Automatable | |
| Urban and regional planners | Non- Automatable | |
| Social workers | Non- Automatable | |
| Clergy and religious workers | Non- Automatable | |
| Welfare service workers | Non- Automatable | |
| Lawyers and judges | Automatable | |
| Writers and authors | Non- Automatable | |
| Technical writers | Non- Automatable | |
| Designers | Non- Automatable | |
| Musicians and composers | Non- Automatable | |
| Actors, directors, and producers | Non- Automatable | |
| Painters, sculptors, craft-artists, and print-makers | Fully Automatable | |
| Photographers | Fully Automatable | |
| Dancers | Non- Automatable | |
| Art/entertainment performers and related occs | Non- Automatable | |
| Editors and reporters | Non- Automatable | |
| Announcers | Non- Automatable | |
| Athletes, sports instructors, and officials | Automatable | |
| Clinical laboratory technologies and technicians | Fully Automatable | |
| Dental hygienists | Fully Automatable | |
| Health record technologists and technicians | Fully Automatable | |
| Radiologic technologists and technicians | Fully Automatable | |
| Licensed practical nurses | Non- Automatable | |
| Health technologists and technicians, n.e.c. | Automatable | |
| Engineering technicians | Non- Automatable | |
| Drafters | Fully Automatable | |
| Surveryors, cartographers, mapping scientists/techs | Automatable | |
| Biological technicians | Automatable | |
| Chemical technicians | Automatable | |
| Other science technicians | Automatable | |
| Airplane pilots and navigators | Automatable | |
| Air traffic controllers | Fully Automatable | |
| Broadcast equipment operators | Automatable | |
| Computer software developers | Non- Automatable | |
| Programmers of numerically controlled machine tools | Non- Automatable | |
| Legal assistants and paralegals | Fully Automatable | |
| Technicians, n.e.c. | Automatable | |
| Sales supervisors and proprietors | Non- Automatable | |
| Insurance sales occupations | Fully Automatable | |
| Real estate sales occupations | Fully Automatable | |
| Financial service sales occupations | Fully Automatable | |
| Advertising and related sales jobs | Fully Automatable | |
| Sales engineers | Non- Automatable | |
| Salespersons, n.e.c. | Non- Automatable | |
| Retail salespersons and sales clerks | Non- Automatable | |
| Cashiers | Fully Automatable | |
| Door-to-door sales, street sales, and news vendors | Non- Automatable | |
| Sales demonstrators, promoters, and models | Automatable | |
| Office supervisors | Fully Automatable | |
| Computer and peripheral equipment operators | Non- Automatable | |
| Secretaries and stenographers | Fully Automatable | |
| Typists | Fully Automatable | |
| Interviewers, enumerators, and surveyors | Non- Automatable | |
| Hotel clerks | Fully Automatable | |
| Transportation ticket and reservation agents | Fully Automatable | |
| Receptionists and other information clerks | Fully Automatable | |
| Correspondence and order clerks | Fully Automatable | |
| Human resources clerks, excl payroll and timekeeping | Fully Automatable | |
| Library assistants | Automatable | |
| File clerks | Fully Automatable | |
| Records clerks | Fully Automatable | |
| Bookkeepers and accounting and auditing clerks | Fully Automatable | |
| Payroll and timekeeping clerks | Fully Automatable | |
| Billing clerks and related financial records processing | Fully Automatable | |
| Mail and paper handlers | Fully Automatable | |
| Office machine operators, n.e.c. | Fully Automatable | |
| Telephone operators | Fully Automatable | |
| Other telecom operators | Fully Automatable | |
| Postal clerks, exluding mail carriers | Fully Automatable | |
| Mail carriers for postal service | Fully Automatable | |
| Mail clerks, outside of post office | Fully Automatable | |
| Messengers | Fully Automatable | |
| Dispatchers | Fully Automatable | |
| Shipping and receiving clerks | Fully Automatable | |
| Stock and inventory clerks | Fully Automatable | |
| Meter readers | Fully Automatable | |
| Weighers, measurers, and checkers | Fully Automatable | |
| Material recording, sched., prod., plan., expediting cl. | Fully Automatable | |
| Insurance adjusters, examiners, and investigators | Fully Automatable | |
| Customer service reps, invest., adjusters, excl. insur. | Fully Automatable | |
| Eligibility clerks for government prog., social welfare | Fully Automatable | |
| Bill and account collectors | Fully Automatable | |
| General office clerks | Fully Automatable | |
| Bank tellers | Fully Automatable | |
| Proofreaders | Fully Automatable | |
| Data entry keyers | Fully Automatable | |
| Statistical clerks | Fully Automatable | |
| Teacher's aides | Automatable | |
| Administrative support jobs, n.e.c. | Fully Automatable | |
| Housekeepers, maids, butlers, and cleaners | Automatable | |
| Laundry and dry cleaning workers | Fully Automatable | |
| Supervisors of guards | Non- Automatable | |
| Fire fighting, fire prevention, and fire inspection occs | Automatable | |
| Police and detectives, public service | Non- Automatable | |
| Sheriffs, bailiffs, correctional institution officers | Non- Automatable | |
| Crossing guards | Non- Automatable | |
| Guards and police, except public service | Fully Automatable | |
| Protective service, n.e.c. | Automatable | |
| Supervisors of food preparation and service | Non- Automatable | |
| Bartenders | Automatable | |
| Waiters and waitresses | Automatable | |
| Cooks | Fully Automatable | |
| Food preparation workers | Automatable | |
| Miscellanious food preparation and service workers | Automatable | |
| Dental Assistants | Fully Automatable | |
| Health and nursing aides | Non- Automatable | |
| Supervisors of cleaning and building service | Non- Automatable | |
| Superv. of landscaping, lawn service, groundskeeping | Automatable | |
| Gardeners and groundskeepers | Automatable | |
| Janitors | Automatable | |
| Pest control occupations | Fully Automatable | |
| Barbers | Non- Automatable | |
| Hairdressers and cosmetologists | Non- Automatable | |
| Recreation facility attendants | Automatable | |
| Guides | Automatable | |
| Ushers | Automatable | |
| Baggage porters, bellhops and concierges | Automatable | |
| Recreation and fitness workers | Automatable | |
| Motion picture projectionists | Fully Automatable | |
| Child care workers | Non- Automatable | |
| Personal service occupations, n.e.c | Automatable | |
| Supervisors of personal service jobs, n.e.c | Automatable | |
| Public transportation attendants and inspectors | Automatable | |
| Animal caretakers, except farm | Non- Automatable | |
| Farmers (owners and tenants) | Fully Automatable | |
| Farm managers | Fully Automatable | |
| Farm workers, incl. nursery farming | Fully Automatable | |
| Graders and sorters of agricultural products | Fully Automatable | |
| Inspectors of agricultural products | Fully Automatable | |
| Timber, logging, and forestry workers | Fully Automatable | |
| Fishers, marine life cultivators, hunters, and kindred | Fully Automatable | |
| Supervisors of mechanics and repairers | Non- Automatable | |
| Automobile mechanics and repairers | Non- Automatable | |
| Bus, truck, and stationary engine mechanics | Non- Automatable | |
| Aircraft mechanics | Non- Automatable | |
| Small engine repairers | Non- Automatable | |
| Auto body repairers | Fully Automatable | |
| Heavy equipement and farm equipment mechanics | Non- Automatable | |
| Industrial machinery repairers | Non- Automatable | |
| Machinery maintenance occupations | Fully Automatable | |
| Repairers of industrial electrical equipment | Fully Automatable | |
| Repairers of data processing equipment | Fully Automatable | |
| Repairers of household appliances and power tools | Non- Automatable | |
| Telecom and line installers and repairers | Non- Automatable | |
| Repairers of electrical equipment, n.e.c. | Non- Automatable | |
| Heating, air conditioning, and refrigeration mechanics | Non- Automatable | |
| Precision makers, repairers, and smiths | Fully Automatable | |
| Locksmiths and safe repairers | Automatable | |
| Repairers of mechanical controls and valves | Non- Automatable | |
| Elevator installers and repairers | Non- Automatable | |
| Millwrights | Non- Automatable | |
| Mechanics and repairers, n.e.c. | Non- Automatable | |
| Supervisors of construction work | Non- Automatable | |
| Masons, tilers, and carpet installers | Automatable | |
| Carpenters | Non- Automatable | |
| Drywall installers | Non- Automatable | |
| Electricians | Non- Automatable | |
| Electric power installers and repairers | Non- Automatable | |
| Painters, construction and maintenance | Automatable | |
| Paperhangers | Automatable | |
| Plasterers | Automatable | |
| Plumbers, pipe fitters, and steamfitters | Non- Automatable | |
| Concrete and cement workers | Automatable | |
| Glaziers | Non- Automatable | |
| Insulation workers | Non- Automatable | |
| Paving, surfacing, and tamping equipment operators | Non- Automatable | |
| Roofers and slaters | Non- Automatable | |
| Structural metal workers | Non- Automatable | |
| Drillers of earth | Fully Automatable | |
| Misc. construction and related occupations | Automatable | |
| Drillers of oil wells | Fully Automatable | |
| Explosives workers | Automatable | |
| Miners | Automatable | |
| Other mining occupations | Automatable | |
| Production supervisors or foremen | Automatable | |
| Tool and die makers and die setters | Fully Automatable | |
| Machinists | Fully Automatable | |
| Boilermakers | Fully Automatable | |
| Precision grinders and fitters | Fully Automatable | |
| Patternmakers and model makers | Fully Automatable | |
| Engravers | Fully Automatable | |
| Other metal and plastic workers | Automatable | |
| Cabinetmakers and bench carpeters | Non- Automatable | |
| Furniture/wood finishers, other prec. wood workers | Fully Automatable | |
| Dressmakers, seamstresses, and tailors | Automatable | |
| Upholsterers | Fully Automatable | |
| Shoemakers, other prec. apparel and fabric workers | Fully Automatable | |
| Hand molders and shapers, except jewelers | Fully Automatable | |
| Optical goods workers | Fully Automatable | |
| Dental laboratory and medical applicance technicians | Automatable | |
| Bookbinders | Fully Automatable | |
| Other precision and craft workers | Fully Automatable | |
| Butchers and meat cutters | Fully Automatable | |
| Bakers | Fully Automatable | |
| Batch food makers | Fully Automatable | |
| Water and sewage treatment plant operators | Automatable | |
| Power plant operators | Fully Automatable | |
| Plant and system operators, stationary engineers | Automatable | |
| Other plant and system operators | Automatable | |
| Lathe, milling, and turning machine operatives | Fully Automatable | |
| Punching and stamping press operatives | Fully Automatable | |
| Rollers, roll hands, and finishers of metal | Non- Automatable | |
| Drilling and boring machine operators | Fully Automatable | |
| Grinding, abrading, buffing, and polishing workers | Fully Automatable | |
| Forge and hammer operators | Automatable | |
| Molders and casting machine operators | Fully Automatable | |
| Metal platers | Fully Automatable | |
| Heat treating equipment operators | Automatable | |
| Sawing machine operators and sawyers | Fully Automatable | |
| Nail, tacking, shaping and joining mach ops (wood) | Fully Automatable | |
| Other woodworking machine operators | Fully Automatable | |
| Printing machine operators, n.e.c. | Fully Automatable | |
| Typesetters and compositors | Fully Automatable | |
| Winding and twisting textile and apparel operatives | Fully Automatable | |
| Knitters, loopers, and toppers textile operatives | Fully Automatable | |
| Textile cutting and dyeing machine operators | Fully Automatable | |
| Textile sewing machine operators | Fully Automatable | |
| Shoemaking machine operators | Fully Automatable | |
| Clothing pressing machine operators | Automatable | |
| Miscellanious textile machine operators | Fully Automatable | |
| Cementing and gluing machine operators | Fully Automatable | |
| Packers, fillers, and wrappers | Fully Automatable | |
| Extruding and forming machine operators | Fully Automatable | |
| Mixing and blending machine operators | Fully Automatable | |
| Separating, filtering, and clarifying machine operators | Non- Automatable | |
| Food roasting and baking machine operators | Fully Automatable | |
| Washing, cleaning, and pickling machine operators | Fully Automatable | |
| Paper folding machine operators | Fully Automatable | |
| Furnance, kiln, and oven operators, apart from food | Automatable | |
| Slicing, cutting, crushing and grinding machine | Fully Automatable | |
| Photographic process workers | Automatable |  |
| Machine operators, n.e.c. | Automatable | |
| Welders, solderers, and metal cutters | Automatable | |
| Assemblers of electrical equipment | Fully Automatable | |
| Painting and decoration occupations | Automatable | |
| Production checkers, graders, and sorters in manufacturing | Fully Automatable | |
| Supervisors of motor vehicle transportation | Non- Automatable | |
| Truck, delivery, and tractor drivers | Fully Automatable | |
| Bus drivers | Fully Automatable | |
| Taxi cab drivers and chauffeurs | Fully Automatable | |
| Parking lot attendants | Automatable | |
| Railroad conductors and yardmasters | Non- Automatable | |
| Locomotive operators: engineers and firemen | Automatable | |
| Railroad brake, coupler, and switch operators | Automatable | |
| Ship crews and marine engineers | Non- Automatable | |
| Miscellanious transportation occupations | Automatable | |
| Operating engineers of construction equipment | Non- Automatable | |
| Crane, derrick, winch, hoist, longshore operators | Non- Automatable | |
| Excavating and loading machine operators | Automatable | |
| Stevedores and misc. material moving occupations | Automatable | |
| Helpers, constructions | Automatable | |
| Helpers, surveyors | Automatable | |
| Construction laborers | Fully Automatable | |
| Production helpers | Fully Automatable | |
| Garbage and recyclable material collectors | Fully Automatable | |
| Machine feeders and offbearers | Automatable | |
| Garage and service station related occupations | Fully Automatable | |
| Vehicle washers and equipment cleaners | Fully Automatable | |
| Packers and packagers by hand | Fully Automatable | |
| Laborers, freight, stock, and material handlers, n.e.c. | Automatable | |
