# Supplementary material for: Automation and the changing nature of work
Source: PLoS One. 2022 May 5;17(5):e0266326. doi: 10.1371/journal.pone.0266326 (PMC9071144; doi:10.1371/journal.pone.0266326)
Supplement: S1 Appendix — (DOCX) [file pone.0266326.s002.docx]

S2 Appendix. Individual Country Analysis.

S3 Appendix. Individual country analysis Austria

Table A1 Ability Lasso Analysis

| ONET Item | Description |  |  | Coefficient |
| --- | --- | --- | --- | --- |
| Arm-Hand Steadiness | The ability to keep your hand and arm steady while moving your arm or while holding your arm and hand in one position. | Fine manipulative abilities | Psychomotor abilities | -4.05 |
| Auditory Attention | The ability to focus on a single source of sound in the presence of other distracting sounds. | Auditory and speech abilities | Sensory Abilities | 0 |
| Category Flexibility | The ability to generate or use different sets of rules for combining or grouping things in different ways. | Idea Generation and Reasoning Abilities | Cognitive Abilities | -1.687 |
| Control Precision | The ability to quickly and repeatedly adjust the controls of a machine or a vehicle to exact positions. | Control Movement Abilities | Psychomotor Abilities | 1.577 |
| Deductive Reasoning | The ability to apply general rules to specific problems to produce answers that make sense. | Idea Generation and Reasoning Abilities | Cognitive Abilities | -6.859 |
| Depth Perception | The ability to judge which of several objects is closer or farther away from you, or to judge the distance between you and an object. | Visual Abilities | Sensory Abilities | 0 |
| Dynamic Flexibility | The ability to quickly and repeatedly bend, stretch, twist, or reach out with your body, arms, and/or legs. | Flexibility, Balance and Coordination | Physical Abilities | 3.084 |
| Dynamic Strength | The ability to exert muscle force repeatedly or continuously over time. This involves muscular endurance and resistance to muscle fatigue. | Physical Strength | Physical Abilities | -2.639 |
| Explosive Strength | The ability to use short bursts of muscle force to propel oneself (as in jumping or sprinting), or to throw an object. | Physical Strength | Physical Abilities | 1.665 |
| Extent Flexibility | The ability to bend, stretch, twist, or reach with your body, arms, and/or legs. | Flexibility, balance and coordination | Physical Abilities | -2.926 |
| Far Vision | The ability to see details at a distance. | Visual Abilities | Sensory Abilities | -3.724 |
| Finger Dexterity | The ability to make precisely coordinated movements of the fingers of one or both hands to grasp, manipulate, or assemble very small objects. | Fine manipulative abilities | Psychomotor abilities | 3.201 |
| Flexibility of Closure | The ability to identify or detect a known pattern (a figure, object, word, or sound) that is hidden in other distracting material. | Perecptual Abilities | Cognitive abilities | 0 |
| Fluency of ideas | The ability to come up with a number of ideas about a topic (the number of ideas is important, not their quality, correctness, or creativity). | Ideas generation and reasoning abilities | Cognitive abilities | -4.157 |
| Glare sensitivity | The ability to see objects in the presence of glare or bright lighting. | Visual abilities | Sensory abilities | -2.581 |
| Gross Body Co-ordination | The ability to coordinate the movement of your arms, legs, and torso together when the whole body is in motion. | Flexibility, Balance and Coordination | Physical Abilities | 0 |
| Gross Body Equilibrium | The ability to keep or regain your body balance or stay upright when in an unstable position. | Flexibility, Balance and Coordination | Physical Abilities | 0 |
| Hearing Sensitivity | The ability to detect or tell the differences between sounds that vary in pitch and loudness. | Auditory and Speech abilities | Sensory Abilities | -1.663 |
| Inductive Reasoning | The ability to combine pieces of information to form general rules or conclusions (includes finding a relationship among seemingly unrelated events). | Idea Generation and Reasoning Abilities | Cognitive Abilities | 0 |
| Information Ordering | The ability to arrange things or actions in a certain order or pattern according to a specific rule or set of rules (e.g., patterns of numbers, letters, words, pictures, mathematical operations). | Idea Generation and Reasoning Abilities | Cognitive Abilities | 0 |
| Manual Dexterity | The ability to quickly move your hand, your hand together with your arm, or your two hands to grasp, manipulate, or assemble objects. | Fine Manipulative Abilities | Psychomotor Abilities | 0 |
| Math Reasoning | The ability to choose the right mathematical methods or formulas to solve a problem. | Quantitative Abilities | Cognitive Abilities | 1.948 |
| Memorization | Abilities related to the recall of available information | Quantitative Abilities | Cognitive Abilities | 0 |
| Multi Limb Co-ordination | The ability to coordinate two or more limbs (for example, two arms, two legs, or one leg and one arm) while sitting, standing, or lying down. It does not involve performing the activities while the whole body is in motion. | Control movement abilities | Psychomotor Abilities | 2.046 |
| Near Vision | The ability to see details at close range (within a few feet of the observer) | Near Vision | Visual Abilities | -1.889 |
| Night Vision | The ability to see under low light conditions | Near Vision | Visual Abilities | 0 |
| Number Facility | The ability to add, subtract, multiply, or divide quickly and correctly. | Quantitative Abilities | Cognitive Abilities | -2.215 |
| Oral Comprehension | The ability to listen to and understand information and ideas presented through spoken words and sentences. | Verbal Abilities | Cognitive Abilities | 0 |
| Oral Expression | The ability to communicate information and ideas in speaking so others will understand. | Verbal Abilities | Cognitive Abilities | 5.3 |
| Originality |  |  |  | 0.489 |
| Perceptual Speed | The ability to quickly and accurately compare similarities and differences among sets of letters, numbers, objects, pictures, or patterns. The things to be compared may be presented at the same time or one after the other. This ability also includes comparing a presented object with a remembered object. | Perceptual Abilities | Cognitive Abilities | -4.268 |
| Perceptual Vision | The ability to see objects or movement of objects to one's side when the eyes are looking ahead. | Visual Abilities | Sensory Abilities | 1.425 |
| Problem Sensitivity | The ability to tell when something is wrong or is likely to go wrong. It does not involve solving the problem, only recognizing there is a problem. | Idea generation and Reasoning Abilities | Cognitive Abilities | 2.676 |
| Rate Control | The ability to time your movements or the movement of a piece of equipment in anticipation of changes in the speed and/or direction of a moving object or scene. | Control Movement Abilities | Psychomotor Abilities | 3.512 |
| Reaction Time | The ability to quickly respond (with the hand, finger, or foot) to a signal (sound, light, picture) when it appears. | Reaction Time and Speed Abilities | Psychomotor Abilities | 0 |
| Response Orientation | The ability to choose quickly between two or more movements in response to two or more different signals (lights, sounds, pictures). It includes the speed with which the correct response is started with the hand, foot, or other body part. | Control Movement Abilities | Psychomotor Abilities | -3.375 |
| Selective Attention | The ability to concentrate on a task over a period of time without being distracted. | Attentiveness | Cognitive Abilities | 0.837 |
| Sound Localisation | The ability to tell the direction from which a sound originated. | Auditory and Speech Abilities | Sensory Abilities | -0.809 |
| Spatial Orientation | The ability to know your location in relation to the environment or to know where other objects are in relation to you. | Spatial Abilities | Cognitive Abilities | 1.711 |
| Speech Clarity | The ability to speak clearly so others can understand you. | Auditory and Speech Abilities | Sensory Abilities | 0 |
| Speech Recognition | The ability to identify and understand the speech of another person. | Auditory and Speech Abilities | Sensory Abilities | -1.291 |
| Speed of Closure | The ability to quickly make sense of, combine, and organize information into meaningful patterns. | Perceptual Abilities | Cognitive Abilities | 5.619 |
| Speed of Limb Movement | The ability to quickly move the arms and legs. | Reaction Time and Speed Abilities | Psychomotor Abilities | 0 |
| Stamina | The ability to exert yourself physically over long periods of time without getting winded or out of breath. | Endurance | Physical Abilities | 1.518 |
| Static Strength | The ability to exert maximum muscle force to lift, push, pull, or carry objects. | Physical Strength Abilities | Physical Abilities | 0.952 |
| Time Sharing | The ability to shift back and forth between two or more activities or sources of information (such as speech, sounds, touch, or other sources). | Attentiveness | Cognitive Abilities | 1.185 |
| Trunk Strength | The ability to use your abdominal and lower back muscles to support part of the body repeatedly or continuously over time without 'giving out' or fatiguing. | Physical Strength Abilities | Physical Abilities | 0 |
| Visual Color Discrimination | The ability to match or detect differences between colors, including shades of color and brightness. | Visual Abilities | Sensory Abilities | 4.615 |
| Visualisation | The ability to imagine how something will look after it is moved around or when its parts are moved or rearranged. | Spatial Abilities | Cognitive Abilities | -0.213 |
| Wrist-Finger Speed | The ability to make fast, simple, repeated movements of the fingers, hands, and wrists. | Reaction Time and Speed Abilities | Psychomotor Abilities | 0 |
| Written Comprehension | The ability to read and understand information and ideas presented in writing. | Verbal Abilities | Cognitive Abilities | 0 |
| Written Expression | The ability to communicate information and ideas in writing so others will understand. | Verbal Abilities | Cognitive Abilities | 0 |
| R Squared = 0.98 | |  |  | 154692 |

Table A2 Austria Skills Lasso Analysis

| ONET Item | Description |  |  | Coefficient |
| --- | --- | --- | --- | --- |
| Active Learning | Understanding the implications of new information for both current and future problem-solving and decision-making. | Process | Basic Skills | 0.144 |
| Active Listening | Giving full attention to what other people are saying, taking time to understand the points being made, asking questions as appropriate, and not interrupting at inappropriate times. | Content | Basic Skills | -0.805 |
| Complex Problem Solving | Identifying complex problems and reviewing related information to develop and evaluate options and implement solutions. | Complex Problem Solving | Cross Functional Skills | -3.301 |
| Coordination | Adjusting actions in relation to others' actions. | Social Skills | Cross Functional Skills | -6.276 |
| Critical Thinking | Using logic and reasoning to identify the strengths and weaknesses of alternative solutions, conclusions or approaches to problems. | Process | Basic Skills | -3.403 |
| Equipment Maintenance | Performing routine maintenance on equipment and determining when and what kind of maintenance is needed. | Technical Skills | Cross Functional Skills | 0 |
| Equipment Selection | Determining the kind of tools and equipment needed to do a job. | Technical Skills | Cross Functional Skills | 2.149 |
| Installation | Installing equipment, machines, wiring, or programs to meet specifications. | Technical Skills | Cross Functional Skills | 0.923 |
| Instructing | Teaching others how to do something. | Social Skills | Cross Functional Skills | -2.239 |
| Judgement and Decision Making | Considering the relative costs and benefits of potential actions to choose the most appropriate one. | Systems Skills | Cross Functional Skills | 6.807 |
| Learning Strategies | Selecting and using training/instructional methods and procedures appropriate for the situation when learning or teaching new things. | Systems Skills | Cross Functional Skills | 0.184 |
| Management of Financial Resources | Determining how money will be spent to get the work done, and accounting for these expenditures. | Resource Management Skills | Cross functional Skills | 0.235 |
| Management of Material Resources | Obtaining and seeing to the appropriate use of equipment, facilities, and materials needed to do certain work. | Resource Management Skills | Cross functional Skills | -1.102 |
| Management of Personell Resources | Motivating, developing, and directing people as they work, identifying the best people for the job. | Resource Management Skills | Cross functional Skills | 1.371 |
| Mathematics | Using mathematics to solve problems. | Content | Basic Skills | 0 |
| Monitoring | Monitoring/Assessing performance of yourself, other individuals, or organizations to make improvements or take corrective action. | Process | Basic Skills | -6.658 |
| Negotiation | Bringing others together and trying to reconcile differences. | Social Skills | Cross functional Skills | 1.736 |
| Operation Monitoring | Watching gauges, dials, or other indicators to make sure a machine is working properly. | Technical Skills | Cross Functional Skills | 3.709 |
| Operation and Control | Controlling operations of equipment or systems. | Technical Skills | Cross Functional Skills | -0.903 |
| Operations Analysis | Analyzing needs and product requirements to create a design. | Technical Skills | Cross Functional Skills | -1.407 |
| Persuasion | Persuading others to change their minds or behavior. | Social Skills | Cross functional Skills | 0 |
| Programming | Writing computer programs for various purposes. | Technical Skills | Cross functional Skills | 0 |
| Quality Control Analysis | Conducting tests and inspections of products, services, or processes to evaluate quality or performance. | Technical Skills | Cross functional Skills | -0.436 |
| Reading Comprehension | Understanding written sentences and paragraphs in work related documents. | Content | Basic Skills | -2.055 |
| Repairing | Repairing machines or systems using the needed tools. | Technical Skills | Cross Functional Skills | -2.826 |
| Science | Using scientific rules and methods to solve problems. | Content | Basic Skills | 0.755 |
| Service Orientation | Actively looking for ways to help people. | Social Skills | Cross Functional Skills | -0.93 |
| Social Perceptiveness | Being aware of others' reactions and understanding why they react as they do. | Social Skills | Cross Functional Skills | 0 |
| Speaking | Talking to others to convey information effectively. | Content | Basic Skills | 3.468 |
| System Analysis | Determining how a system should work and how changes in conditions, operations, and the environment will affect outcomes. | Systems Skills | Cross Functional Skills | 0 |
| System Evaluation | Identifying measures or indicators of system performance and the actions needed to improve or correct performance, relative to the goals of the system. | Systems Skills | Cross Functional Skills | 3.193 |
| Technology Design | Generating or adapting equipment and technology to serve user needs. | Technical Skills | Cross Functional Skills | -3.514 |
| Time Management | Managing one's own time and the time of others. | Resource Management Skills | Cross Functional Skills | 3.156 |
| Troubleshooting | Determining causes of operating errors and deciding what to do about it. | Technical Skills | Cross Functional Skills | 0 |
| Writing | Communicating effectively in writing as appropriate for the needs of the audience. | Content | Basic Skills | 1.854 |
| R squared = 0.82 |  |  | N= | 154692 |

Table A3 Austria People, Brains, Brawn Analysis

|  |  |
| --- | --- |
|  | Austria – EU LFS |
| People | -0.016*** |
|  | (0.001) |
| Brains | -0.073*** |
|  | (0.001) |
| Brawn | 0.038*** |
|  | (0.001) |
| People * Brains | -0.006*** |
|  | (0.000) |
| People* Brawn | 0.025*** |
|  | (0.000) |
| Brains* Brawn | -0.030*** |
|  | (0.000) |
| N | 154,547 |
| R Squared | 13% |

***, **, * denotes significance at the 1%, 5% and 10% levels respectively

S4 Appendix. Individual country analysis Belgium

Table B1 Belgium Ability Lasso Analysis

| ONET Item | Description |  |  | Coefficient |
| --- | --- | --- | --- | --- |
| Arm-Hand Steadiness | The ability to keep your hand and arm steady while moving your arm or while holding your arm and hand in one position. | Fine manipulative abilities | Psychomotor abilities | -3.704 |
| Auditory Attention | The ability to focus on a single source of sound in the presence of other distracting sounds. | Auditory and speech abilities | Sensory Abilities | 0 |
| Category Flexibility | The ability to generate or use different sets of rules for combining or grouping things in different ways. | Idea Generation and Reasoning Abilities | Cognitive Abilities | -1.494 |
| Control Precision | The ability to quickly and repeatedly adjust the controls of a machine or a vehicle to exact positions. | Control Movement Abilities | Psychomotor Abilities | 1.515 |
| Deductive Reasoning | The ability to apply general rules to specific problems to produce answers that make sense. | Idea Generation and Reasoning Abilities | Cognitive Abilities | -7.242 |
| Depth Perception | The ability to judge which of several objects is closer or farther away from you, or to judge the distance between you and an object. | Visual Abilities | Sensory Abilities | 0 |
| Dynamic Flexibility | The ability to quickly and repeatedly bend, stretch, twist, or reach out with your body, arms, and/or legs. | Flexibility, Balance and Coordination | Physical Abilities | 2.57 |
| Dynamic Strength | The ability to exert muscle force repeatedly or continuously over time. This involves muscular endurance and resistance to muscle fatigue. | Physical Strength | Physical Abilities | -2.458 |
| Explosive Strength | The ability to use short bursts of muscle force to propel oneself (as in jumping or sprinting), or to throw an object. | Physical Strength | Physical Abilities | 1.567 |
| Extent Flexibility | The ability to bend, stretch, twist, or reach with your body, arms, and/or legs. | Flexibility, balance and coordination | Physical Abilities | -2.691 |
| Far Vision | The ability to see details at a distance. | Visual Abilities | Sensory Abilities | -3.246 |
| Finger Dexterity | The ability to make precisely coordinated movements of the fingers of one or both hands to grasp, manipulate, or assemble very small objects. | Fine manipulative abilities | Psychomotor abilities | 2.822 |
| Flexibility of Closure | The ability to identify or detect a known pattern (a figure, object, word, or sound) that is hidden in other distracting material. | Perecptual Abilities | Cognitive abilities | 0 |
| Fluency of ideas | The ability to come up with a number of ideas about a topic (the number of ideas is important, not their quality, correctness, or creativity). | Ideas generation and reasoning abilities | Cognitive abilities | -3.955 |
| Glare sensitivity | The ability to see objects in the presence of glare or bright lighting. | Visual abilities | Sensory abilities | -2.16 |
| Gross Body Co-ordination | The ability to coordinate the movement of your arms, legs, and torso together when the whole body is in motion. | Flexibility, Balance and Coordination | Physical Abilities | 0 |
| Gross Body Equilibrium | The ability to keep or regain your body balance or stay upright when in an unstable position. | Flexibility, Balance and Coordination | Physical Abilities | -0.145 |
| Hearing Sensitivity | The ability to detect or tell the differences between sounds that vary in pitch and loudness. | Auditory and Speech abilities | Sensory Abilities | -1.93 |
| Inductive Reasoning | The ability to combine pieces of information to form general rules or conclusions (includes finding a relationship among seemingly unrelated events). | Idea Generation and Reasoning Abilities | Cognitive Abilities | 0 |
| Information Ordering | The ability to arrange things or actions in a certain order or pattern according to a specific rule or set of rules (e.g., patterns of numbers, letters, words, pictures, mathematical operations). | Idea Generation and Reasoning Abilities | Cognitive Abilities | 0 |
| Manual Dexterity | The ability to quickly move your hand, your hand together with your arm, or your two hands to grasp, manipulate, or assemble objects. | Fine Manipulative Abilities | Psychomotor Abilities | 0 |
| Math Reasoning | The ability to choose the right mathematical methods or formulas to solve a problem. | Quantitative Abilities | Cognitive Abilities | 2.544 |
| Memorization | Abilities related to the recall of available information | Quantitative Abilities | Cognitive Abilities | 0 |
| Multi Limb Co-ordination | The ability to coordinate two or more limbs (for example, two arms, two legs, or one leg and one arm) while sitting, standing, or lying down. It does not involve performing the activities while the whole body is in motion. | Control movement abilities | Psychomotor Abilities | 1.836 |
| Near Vision | The ability to see details at close range (within a few feet of the observer) | Near Vision | Visual Abilities | -1.694 |
| Night Vision | The ability to see under low light conditions | Near Vision | Visual Abilities | 0 |
| Number Facility | The ability to add, subtract, multiply, or divide quickly and correctly. | Quantitative Abilities | Cognitive Abilities | -2.601 |
| Oral Comprehension | The ability to listen to and understand information and ideas presented through spoken words and sentences. | Verbal Abilities | Cognitive Abilities | -1.365 |
| Oral Expression | The ability to communicate information and ideas in speaking so others will understand. | Verbal Abilities | Cognitive Abilities | 6.102 |
| Originality |  |  |  | 0.378 |
| Perceptual Speed | The ability to quickly and accurately compare similarities and differences among sets of letters, numbers, objects, pictures, or patterns. The things to be compared may be presented at the same time or one after the other. This ability also includes comparing a presented object with a remembered object. | Perceptual Abilities | Cognitive Abilities | -4.276 |
| Perceptual Vision | The ability to see objects or movement of objects to one's side when the eyes are looking ahead. | Visual Abilities | Sensory Abilities | 1.66 |
| Problem Sensitivity | The ability to tell when something is wrong or is likely to go wrong. It does not involve solving the problem, only recognizing there is a problem. | Idea generation and Reasoning Abilities | Cognitive Abilities | 3.406 |
| Rate Control | The ability to time your movements or the movement of a piece of equipment in anticipation of changes in the speed and/or direction of a moving object or scene. | Control Movement Abilities | Psychomotor Abilities | 3.284 |
| Reaction Time | The ability to quickly respond (with the hand, finger, or foot) to a signal (sound, light, picture) when it appears. | Reaction Time and Speed Abilities | Psychomotor Abilities | 0 |
| Response Orientation | The ability to choose quickly between two or more movements in response to two or more different signals (lights, sounds, pictures). It includes the speed with which the correct response is started with the hand, foot, or other body part. | Control Movement Abilities | Psychomotor Abilities | -3.23 |
| Selective Attention | The ability to concentrate on a task over a period of time without being distracted. | Attentiveness | Cognitive Abilities | 0 |
| Sound Localisation | The ability to tell the direction from which a sound originated. | Auditory and Speech Abilities | Sensory Abilities | -0.801 |
| Spatial Orientation | The ability to know your location in relation to the environment or to know where other objects are in relation to you. | Spatial Abilities | Cognitive Abilities | 1.179 |
| Speech Clarity | The ability to speak clearly so others can understand you. | Auditory and Speech Abilities | Sensory Abilities | 0 |
| Speech Recognition | The ability to identify and understand the speech of another person. | Auditory and Speech Abilities | Sensory Abilities | -1.451 |
| Speed of Closure | The ability to quickly make sense of, combine, and organize information into meaningful patterns. | Perceptual Abilities | Cognitive Abilities | 5.39 |
| Speed of Limb Movement | The ability to quickly move the arms and legs. | Reaction Time and Speed Abilities | Psychomotor Abilities | 0 |
| Stamina | The ability to exert yourself physically over long periods of time without getting winded or out of breath. | Endurance | Physical Abilities | 1.245 |
| Static Strength | The ability to exert maximum muscle force to lift, push, pull, or carry objects. | Physical Strength Abilities | Physical Abilities | 1.293 |
| Time Sharing | The ability to shift back and forth between two or more activities or sources of information (such as speech, sounds, touch, or other sources). | Attentiveness | Cognitive Abilities | 1.282 |
| Trunk Strength | The ability to use your abdominal and lower back muscles to support part of the body repeatedly or continuously over time without 'giving out' or fatiguing. | Physical Strength Abilities | Physical Abilities | 0 |
| Visual Color Discrimination | The ability to match or detect differences between colors, including shades of color and brightness. | Visual Abilities | Sensory Abilities | 4.083 |
| Visualisation | The ability to imagine how something will look after it is moved around or when its parts are moved or rearranged. | Spatial Abilities | Cognitive Abilities | 0.317 |
| Wrist-Finger Speed | The ability to make fast, simple, repeated movements of the fingers, hands, and wrists. | Reaction Time and Speed Abilities | Psychomotor Abilities | 0 |
| Written Comprehension | The ability to read and understand information and ideas presented in writing. | Verbal Abilities | Cognitive Abilities | 0 |
| Written Expression | The ability to communicate information and ideas in writing so others will understand. | Verbal Abilities | Cognitive Abilities | 0 |
| R Squared = 0.98 | |  | N= 72492 | |

Table B2 Belgian Skills Lasso Analysis

| ONET Item | Description |  |  | Coefficient |
| --- | --- | --- | --- | --- |
| Active Learning | Understanding the implications of new information for both current and future problem-solving and decision-making. | Process | Basic Skills | 0.488 |
| Active Listening | Giving full attention to what other people are saying, taking time to understand the points being made, asking questions as appropriate, and not interrupting at inappropriate times. | Content | Basic Skills | -1.757 |
| Complex Problem Solving | Identifying complex problems and reviewing related information to develop and evaluate options and implement solutions. | Complex Problem Solving | Cross Functional Skills | -2.427 |
| Coordination | Adjusting actions in relation to others' actions. | Social Skills | Cross Functional Skills | -4.999 |
| Critical Thinking | Using logic and reasoning to identify the strengths and weaknesses of alternative solutions, conclusions or approaches to problems. | Process | Basic Skills | 0 |
| Equipment Maintenance | Performing routine maintenance on equipment and determining when and what kind of maintenance is needed. | Technical Skills | Cross Functional Skills | -1.299 |
| Equipment Selection | Determining the kind of tools and equipment needed to do a job. | Technical Skills | Cross Functional Skills | 1.507 |
| Installation | Installing equipment, machines, wiring, or programs to meet specifications. | Technical Skills | Cross Functional Skills | 0.04 |
| Instructing | Teaching others how to do something. | Social Skills | Cross Functional Skills | -1.824 |
| Judgement and Decision Making | Considering the relative costs and benefits of potential actions to choose the most appropriate one. | Systems Skills | Cross Functional Skills | 4.357 |
| Learning Strategies | Selecting and using training/instructional methods and procedures appropriate for the situation when learning or teaching new things. | Systems Skills | Cross Functional Skills | 0.877 |
| Management of Financial Resources | Determining how money will be spent to get the work done, and accounting for these expenditures. | Resource Management Skills | Cross functional Skills | 0 |
| Management of Material Resources | Obtaining and seeing to the appropriate use of equipment, facilities, and materials needed to do certain work. | Resource Management Skills | Cross functional Skills | 0 |
| Management of Personell Resources | Motivating, developing, and directing people as they work, identifying the best people for the job. | Resource Management Skills | Cross functional Skills | 0 |
| Mathematics | Using mathematics to solve problems. | Content | Basic Skills | 0 |
| Monitoring | Monitoring/Assessing performance of yourself, other individuals, or organizations to make improvements or take corrective action. | Process | Basic Skills | -6.741 |
| Negotiation | Bringing others together and trying to reconcile differences. | Social Skills | Cross functional Skills | 2.591 |
| Operation Monitoring | Watching gauges, dials, or other indicators to make sure a machine is working properly. | Technical Skills | Cross Functional Skills | 2.707 |
| Operation and Control | Controlling operations of equipment or systems. | Technical Skills | Cross Functional Skills | 0 |
| Operations Analysis | Analyzing needs and product requirements to create a design. | Technical Skills | Cross Functional Skills | -0.836 |
| Persuasion | Persuading others to change their minds or behavior. | Social Skills | Cross functional Skills | -1.59 |
| Programming | Writing computer programs for various purposes. | Technical Skills | Cross functional Skills | 0 |
| Quality Control Analysis | Conducting tests and inspections of products, services, or processes to evaluate quality or performance. | Technical Skills | Cross functional Skills | -1.535 |
| Reading Comprehension | Understanding written sentences and paragraphs in work related documents. | Content | Basic Skills | 0 |
| Repairing | Repairing machines or systems using the needed tools. | Technical Skills | Cross Functional Skills | -0.8 |
| Science | Using scientific rules and methods to solve problems. | Content | Basic Skills | 0.342 |
| Service Orientation | Actively looking for ways to help people. | Social Skills | Cross Functional Skills | 0 |
| Social Perceptiveness | Being aware of others' reactions and understanding why they react as they do. | Social Skills | Cross Functional Skills | -0.631 |
| Speaking | Talking to others to convey information effectively. | Content | Basic Skills | 2.659 |
| System Analysis | Determining how a system should work and how changes in conditions, operations, and the environment will affect outcomes. | Systems Skills | Cross Functional Skills | 0.297 |
| System Evaluation | Identifying measures or indicators of system performance and the actions needed to improve or correct performance, relative to the goals of the system. | Systems Skills | Cross Functional Skills | 2.154 |
| Technology Design | Generating or adapting equipment and technology to serve user needs. | Technical Skills | Cross Functional Skills | -2.974 |
| Time Management | Managing one's own time and the time of others. | Resource Management Skills | Cross Functional Skills | 3.972 |
| Troubleshooting | Determining causes of operating errors and deciding what to do about it. | Technical Skills | Cross Functional Skills | 0.519 |
| Writing | Communicating effectively in writing as appropriate for the needs of the audience. | Content | Basic Skills | 0 |
| R squared = 0.76 |  |  | N= | 72, 492 |

Table B3 Belgium People, Brains, Brawn Analysis

|  |  |
| --- | --- |
|  | Belgium – EU LFS |
| People | -0.046*** |
|  | (0.001) |
| Brains | -0.089*** |
|  | (0.001) |
| Brawn | 0.016*** |
|  | (0.001) |
| People * Brains | -0.005*** |
|  | (0.000) |
| People* Brawn | 0.000* |
|  | (0.000) |
| Brains* Brawn | -0.003*** |
|  | (0.000) |
| N | 72,405 |
| R Squared | 13% |

***, **, * denotes significance at the 1%, 5% and 10% levels respectively

S5 Appendix. Individual country analysis Cyprus

Table C1 Cyprus Ability Lasso Analysis

| ONET Item | Description |  |  | Coefficient |
| --- | --- | --- | --- | --- |
| Arm-Hand Steadiness | The ability to keep your hand and arm steady while moving your arm or while holding your arm and hand in one position. | Fine manipulative abilities | Psychomotor abilities | -0.779 |
| Auditory Attention | The ability to focus on a single source of sound in the presence of other distracting sounds. | Auditory and speech abilities | Sensory Abilities | -0.998 |
| Category Flexibility | The ability to generate or use different sets of rules for combining or grouping things in different ways. | Idea Generation and Reasoning Abilities | Cognitive Abilities | 0 |
| Control Precision | The ability to quickly and repeatedly adjust the controls of a machine or a vehicle to exact positions. | Control Movement Abilities | Psychomotor Abilities | -0.619 |
| Deductive Reasoning | The ability to apply general rules to specific problems to produce answers that make sense. | Idea Generation and Reasoning Abilities | Cognitive Abilities | 0 |
| Depth Perception | The ability to judge which of several objects is closer or farther away from you, or to judge the distance between you and an object. | Visual Abilities | Sensory Abilities | 2.941 |
| Dynamic Flexibility | The ability to quickly and repeatedly bend, stretch, twist, or reach out with your body, arms, and/or legs. | Flexibility, Balance and Coordination | Physical Abilities | 2.821 |
| Dynamic Strength | The ability to exert muscle force repeatedly or continuously over time. This involves muscular endurance and resistance to muscle fatigue. | Physical Strength | Physical Abilities | 0 |
| Explosive Strength | The ability to use short bursts of muscle force to propel oneself (as in jumping or sprinting), or to throw an object. | Physical Strength | Physical Abilities | 0.317 |
| Extent Flexibility | The ability to bend, stretch, twist, or reach with your body, arms, and/or legs. | Flexibility, balance and coordination | Physical Abilities | 0 |
| Far Vision | The ability to see details at a distance. | Visual Abilities | Sensory Abilities | 0 |
| Finger Dexterity | The ability to make precisely coordinated movements of the fingers of one or both hands to grasp, manipulate, or assemble very small objects. | Fine manipulative abilities | Psychomotor abilities | 0.403 |
| Flexibility of Closure | The ability to identify or detect a known pattern (a figure, object, word, or sound) that is hidden in other distracting material. | Perecptual Abilities | Cognitive abilities | 0 |
| Fluency of ideas | The ability to come up with a number of ideas about a topic (the number of ideas is important, not their quality, correctness, or creativity). | Ideas generation and reasoning abilities | Cognitive abilities | 0 |
| Glare sensitivity | The ability to see objects in the presence of glare or bright lighting. | Visual abilities | Sensory abilities | -2.514 |
| Gross Body Co-ordination | The ability to coordinate the movement of your arms, legs, and torso together when the whole body is in motion. | Flexibility, Balance and Coordination | Physical Abilities | -1.158 |
| Gross Body Equilibrium | The ability to keep or regain your body balance or stay upright when in an unstable position. | Flexibility, Balance and Coordination | Physical Abilities | -1.65 |
| Hearing Sensitivity | The ability to detect or tell the differences between sounds that vary in pitch and loudness. | Auditory and Speech abilities | Sensory Abilities | 0 |
| Inductive Reasoning | The ability to combine pieces of information to form general rules or conclusions (includes finding a relationship among seemingly unrelated events). | Idea Generation and Reasoning Abilities | Cognitive Abilities | -2.137 |
| Information Ordering | The ability to arrange things or actions in a certain order or pattern according to a specific rule or set of rules (e.g., patterns of numbers, letters, words, pictures, mathematical operations). | Idea Generation and Reasoning Abilities | Cognitive Abilities | 0 |
| Manual Dexterity | The ability to quickly move your hand, your hand together with your arm, or your two hands to grasp, manipulate, or assemble objects. | Fine Manipulative Abilities | Psychomotor Abilities | 0 |
| Math Reasoning | The ability to choose the right mathematical methods or formulas to solve a problem. | Quantitative Abilities | Cognitive Abilities | 0 |
| Memorization | Abilities related to the recall of available information | Quantitative Abilities | Cognitive Abilities | 3.282 |
| Multi Limb Co-ordination | The ability to coordinate two or more limbs (for example, two arms, two legs, or one leg and one arm) while sitting, standing, or lying down. It does not involve performing the activities while the whole body is in motion. | Control movement abilities | Psychomotor Abilities | 0 |
| Near Vision | The ability to see details at close range (within a few feet of the observer) | Near Vision | Visual Abilities | 2.659 |
| Night Vision | The ability to see under low light conditions | Near Vision | Visual Abilities | 2.05 |
| Number Facility | The ability to add, subtract, multiply, or divide quickly and correctly. | Quantitative Abilities | Cognitive Abilities | -1.513 |
| Oral Comprehension | The ability to listen to and understand information and ideas presented through spoken words and sentences. | Verbal Abilities | Cognitive Abilities | -4.977 |
| Oral Expression | The ability to communicate information and ideas in speaking so others will understand. | Verbal Abilities | Cognitive Abilities | 4.577 |
| Originality |  |  |  | 0 |
| Perceptual Speed | The ability to quickly and accurately compare similarities and differences among sets of letters, numbers, objects, pictures, or patterns. The things to be compared may be presented at the same time or one after the other. This ability also includes comparing a presented object with a remembered object. | Perceptual Abilities | Cognitive Abilities | -2.952 |
| Perceptual Vision | The ability to see objects or movement of objects to one's side when the eyes are looking ahead. | Visual Abilities | Sensory Abilities | 0 |
| Problem Sensitivity | The ability to tell when something is wrong or is likely to go wrong. It does not involve solving the problem, only recognizing there is a problem. | Idea generation and Reasoning Abilities | Cognitive Abilities | 2.6 |
| Rate Control | The ability to time your movements or the movement of a piece of equipment in anticipation of changes in the speed and/or direction of a moving object or scene. | Control Movement Abilities | Psychomotor Abilities | 4.01 |
| Reaction Time | The ability to quickly respond (with the hand, finger, or foot) to a signal (sound, light, picture) when it appears. | Reaction Time and Speed Abilities | Psychomotor Abilities | 0 |
| Response Orientation | The ability to choose quickly between two or more movements in response to two or more different signals (lights, sounds, pictures). It includes the speed with which the correct response is started with the hand, foot, or other body part. | Control Movement Abilities | Psychomotor Abilities | -1.86 |
| Selective Attention | The ability to concentrate on a task over a period of time without being distracted. | Attentiveness | Cognitive Abilities | 0 |
| Sound Localisation | The ability to tell the direction from which a sound originated. | Auditory and Speech Abilities | Sensory Abilities | -0.335 |
| Spatial Orientation | The ability to know your location in relation to the environment or to know where other objects are in relation to you. | Spatial Abilities | Cognitive Abilities | 1.208 |
| Speech Clarity | The ability to speak clearly so others can understand you. | Auditory and Speech Abilities | Sensory Abilities | 0 |
| Speech Recognition | The ability to identify and understand the speech of another person. | Auditory and Speech Abilities | Sensory Abilities | -2.376 |
| Speed of Closure | The ability to quickly make sense of, combine, and organize information into meaningful patterns. | Perceptual Abilities | Cognitive Abilities | 2.328 |
| Speed of Limb Movement | The ability to quickly move the arms and legs. | Reaction Time and Speed Abilities | Psychomotor Abilities | -3.248 |
| Stamina | The ability to exert yourself physically over long periods of time without getting winded or out of breath. | Endurance | Physical Abilities | 3.274 |
| Static Strength | The ability to exert maximum muscle force to lift, push, pull, or carry objects. | Physical Strength Abilities | Physical Abilities | 1.074 |
| Time Sharing | The ability to shift back and forth between two or more activities or sources of information (such as speech, sounds, touch, or other sources). | Attentiveness | Cognitive Abilities | 0 |
| Trunk Strength | The ability to use your abdominal and lower back muscles to support part of the body repeatedly or continuously over time without 'giving out' or fatiguing. | Physical Strength Abilities | Physical Abilities | -0.819 |
| Visual Color Discrimination | The ability to match or detect differences between colors, including shades of color and brightness. | Visual Abilities | Sensory Abilities | 0 |
| Visualisation | The ability to imagine how something will look after it is moved around or when its parts are moved or rearranged. | Spatial Abilities | Cognitive Abilities | -0.93 |
| Wrist-Finger Speed | The ability to make fast, simple, repeated movements of the fingers, hands, and wrists. | Reaction Time and Speed Abilities | Psychomotor Abilities | 0 |
| Written Comprehension | The ability to read and understand information and ideas presented in writing. | Verbal Abilities | Cognitive Abilities | 2.319 |
| Written Expression | The ability to communicate information and ideas in writing so others will understand. | Verbal Abilities | Cognitive Abilities | -2.877 |
| R Squared = 0.95 | |  | N= 30660 | |

Table C2 Cyprus Skills Lasso Analysis

| ONET Item | Description |  |  | Coefficient |
| --- | --- | --- | --- | --- |
| Active Learning | Understanding the implications of new information for both current and future problem-solving and decision-making. | Process | Basic Skills | 2.486 |
| Active Listening | Giving full attention to what other people are saying, taking time to understand the points being made, asking questions as appropriate, and not interrupting at inappropriate times. | Content | Basic Skills | -0.454 |
| Complex Problem Solving | Identifying complex problems and reviewing related information to develop and evaluate options and implement solutions. | Complex Problem Solving | Cross Functional Skills | -1.345 |
| Coordination | Adjusting actions in relation to others' actions. | Social Skills | Cross Functional Skills | -4.872 |
| Critical Thinking | Using logic and reasoning to identify the strengths and weaknesses of alternative solutions, conclusions or approaches to problems. | Process | Basic Skills | 0 |
| Equipment Maintenance | Performing routine maintenance on equipment and determining when and what kind of maintenance is needed. | Technical Skills | Cross Functional Skills | -0.34 |
| Equipment Selection | Determining the kind of tools and equipment needed to do a job. | Technical Skills | Cross Functional Skills | 0.662 |
| Installation | Installing equipment, machines, wiring, or programs to meet specifications. | Technical Skills | Cross Functional Skills | 0 |
| Instructing | Teaching others how to do something. | Social Skills | Cross Functional Skills | -2.963 |
| Judgement and Decision Making | Considering the relative costs and benefits of potential actions to choose the most appropriate one. | Systems Skills | Cross Functional Skills | 0 |
| Learning Strategies | Selecting and using training/instructional methods and procedures appropriate for the situation when learning or teaching new things. | Systems Skills | Cross Functional Skills | 0 |
| Management of Financial Resources | Determining how money will be spent to get the work done, and accounting for these expenditures. | Resource Management Skills | Cross functional Skills | 0 |
| Management of Material Resources | Obtaining and seeing to the appropriate use of equipment, facilities, and materials needed to do certain work. | Resource Management Skills | Cross functional Skills | 0 |
| Management of Personell Resources | Motivating, developing, and directing people as they work, identifying the best people for the job. | Resource Management Skills | Cross functional Skills | 1.858 |
| Mathematics | Using mathematics to solve problems. | Content | Basic Skills | -0.594 |
| Monitoring | Monitoring/Assessing performance of yourself, other individuals, or organizations to make improvements or take corrective action. | Process | Basic Skills | -4.729 |
| Negotiation | Bringing others together and trying to reconcile differences. | Social Skills | Cross functional Skills | 2.41 |
| Operation Monitoring | Watching gauges, dials, or other indicators to make sure a machine is working properly. | Technical Skills | Cross Functional Skills | 1.644 |
| Operation and Control | Controlling operations of equipment or systems. | Technical Skills | Cross Functional Skills | 0 |
| Operations Analysis | Analyzing needs and product requirements to create a design. | Technical Skills | Cross Functional Skills | -0.817 |
| Persuasion | Persuading others to change their minds or behavior. | Social Skills | Cross functional Skills | -2.585 |
| Programming | Writing computer programs for various purposes. | Technical Skills | Cross functional Skills | 0 |
| Quality Control Analysis | Conducting tests and inspections of products, services, or processes to evaluate quality or performance. | Technical Skills | Cross functional Skills | -1.343 |
| Reading Comprehension | Understanding written sentences and paragraphs in work related documents. | Content | Basic Skills | 0 |
| Repairing | Repairing machines or systems using the needed tools. | Technical Skills | Cross Functional Skills | -0.799 |
| Science | Using scientific rules and methods to solve problems. | Content | Basic Skills | 0.874 |
| Service Orientation | Actively looking for ways to help people. | Social Skills | Cross Functional Skills | 0 |
| Social Perceptiveness | Being aware of others' reactions and understanding why they react as they do. | Social Skills | Cross Functional Skills | 0 |
| Speaking | Talking to others to convey information effectively. | Content | Basic Skills | 2.109 |
| System Analysis | Determining how a system should work and how changes in conditions, operations, and the environment will affect outcomes. | Systems Skills | Cross Functional Skills | -3.062 |
| System Evaluation | Identifying measures or indicators of system performance and the actions needed to improve or correct performance, relative to the goals of the system. | Systems Skills | Cross Functional Skills | 5.083 |
| Technology Design | Generating or adapting equipment and technology to serve user needs. | Technical Skills | Cross Functional Skills | -1.835 |
| Time Management | Managing one's own time and the time of others. | Resource Management Skills | Cross Functional Skills | 2.513 |
| Troubleshooting | Determining causes of operating errors and deciding what to do about it. | Technical Skills | Cross Functional Skills | 0.591 |
| Writing | Communicating effectively in writing as appropriate for the needs of the audience. | Content | Basic Skills | 0 |
| R squared = 0.87 |  |  | N= | 30660 |

Table C3 Cyprus People, Brains, Brawn Analysis

|  |  |
| --- | --- |
|  | Cyprus – EU LFS |
| People | -0.026*** |
|  | (0.001) |
| Brains | -0.091*** |
|  | (0.002) |
| Brawn | -0.003*** |
|  | (0.001) |
| People * Brains | 0.004*** |
|  | (0.000) |
| People*Brawn | 0.000*** |
|  | (0.000) |
| Brains*Brawn | 0.002*** |
|  | (0.000) |
| N | 30639 |
| R Squared | 14% |

***, **, * denotes significance at the 1%, 5% and 10% levels respectively

S6 Appendix. Individual country analysis Czech Republic

Table D1 Czech Republic Ability Lasso Analysis

| ONET Item | Description |  |  | Coefficient |
| --- | --- | --- | --- | --- |
| Arm-Hand Steadiness | The ability to keep your hand and arm steady while moving your arm or while holding your arm and hand in one position. | Fine manipulative abilities | Psychomotor abilities | 0 |
| Auditory Attention | The ability to focus on a single source of sound in the presence of other distracting sounds. | Auditory and speech abilities | Sensory Abilities | -0.152 |
| Category Flexibility | The ability to generate or use different sets of rules for combining or grouping things in different ways. | Idea Generation and Reasoning Abilities | Cognitive Abilities | -0.692 |
| Control Precision | The ability to quickly and repeatedly adjust the controls of a machine or a vehicle to exact positions. | Control Movement Abilities | Psychomotor Abilities | 0 |
| Deductive Reasoning | The ability to apply general rules to specific problems to produce answers that make sense. | Idea Generation and Reasoning Abilities | Cognitive Abilities | -5.674 |
| Depth Perception | The ability to judge which of several objects is closer or farther away from you, or to judge the distance between you and an object. | Visual Abilities | Sensory Abilities | 0 |
| Dynamic Flexibility | The ability to quickly and repeatedly bend, stretch, twist, or reach out with your body, arms, and/or legs. | Flexibility, Balance and Coordination | Physical Abilities | 4.142 |
| Dynamic Strength | The ability to exert muscle force repeatedly or continuously over time. This involves muscular endurance and resistance to muscle fatigue. | Physical Strength | Physical Abilities | -2.402 |
| Explosive Strength | The ability to use short bursts of muscle force to propel oneself (as in jumping or sprinting), or to throw an object. | Physical Strength | Physical Abilities | 1.705 |
| Extent Flexibility | The ability to bend, stretch, twist, or reach with your body, arms, and/or legs. | Flexibility, balance and coordination | Physical Abilities | -1.758 |
| Far Vision | The ability to see details at a distance. | Visual Abilities | Sensory Abilities | -2.727 |
| Finger Dexterity | The ability to make precisely coordinated movements of the fingers of one or both hands to grasp, manipulate, or assemble very small objects. | Fine manipulative abilities | Psychomotor abilities | 2.451 |
| Flexibility of Closure | The ability to identify or detect a known pattern (a figure, object, word, or sound) that is hidden in other distracting material. | Perecptual Abilities | Cognitive abilities | 0 |
| Fluency of ideas | The ability to come up with a number of ideas about a topic (the number of ideas is important, not their quality, correctness, or creativity). | Ideas generation and reasoning abilities | Cognitive abilities | -2.925 |
| Glare sensitivity | The ability to see objects in the presence of glare or bright lighting. | Visual abilities | Sensory abilities | -3.347 |
| Gross Body Co-ordination | The ability to coordinate the movement of your arms, legs, and torso together when the whole body is in motion. | Flexibility, Balance and Coordination | Physical Abilities | -0.079 |
| Gross Body Equilibrium | The ability to keep or regain your body balance or stay upright when in an unstable position. | Flexibility, Balance and Coordination | Physical Abilities | 0 |
| Hearing Sensitivity | The ability to detect or tell the differences between sounds that vary in pitch and loudness. | Auditory and Speech abilities | Sensory Abilities | -1.92 |
| Inductive Reasoning | The ability to combine pieces of information to form general rules or conclusions (includes finding a relationship among seemingly unrelated events). | Idea Generation and Reasoning Abilities | Cognitive Abilities | 0 |
| Information Ordering | The ability to arrange things or actions in a certain order or pattern according to a specific rule or set of rules (e.g., patterns of numbers, letters, words, pictures, mathematical operations). | Idea Generation and Reasoning Abilities | Cognitive Abilities | -2.695 |
| Manual Dexterity | The ability to quickly move your hand, your hand together with your arm, or your two hands to grasp, manipulate, or assemble objects. | Fine Manipulative Abilities | Psychomotor Abilities | -1.786 |
| Math Reasoning | The ability to choose the right mathematical methods or formulas to solve a problem. | Quantitative Abilities | Cognitive Abilities | 2.496 |
| Memorization | Abilities related to the recall of available information | Quantitative Abilities | Cognitive Abilities | 0 |
| Multi Limb Co-ordination | The ability to coordinate two or more limbs (for example, two arms, two legs, or one leg and one arm) while sitting, standing, or lying down. It does not involve performing the activities while the whole body is in motion. | Control movement abilities | Psychomotor Abilities | 2.123 |
| Near Vision | The ability to see details at close range (within a few feet of the observer) | Near Vision | Visual Abilities | 0 |
| Night Vision | The ability to see under low light conditions | Near Vision | Visual Abilities | 2.058 |
| Number Facility | The ability to add, subtract, multiply, or divide quickly and correctly. | Quantitative Abilities | Cognitive Abilities | -2.991 |
| Oral Comprehension | The ability to listen to and understand information and ideas presented through spoken words and sentences. | Verbal Abilities | Cognitive Abilities | 0 |
| Oral Expression | The ability to communicate information and ideas in speaking so others will understand. | Verbal Abilities | Cognitive Abilities | 4.978 |
| Originality |  |  |  | 0.76 |
| Perceptual Speed | The ability to quickly and accurately compare similarities and differences among sets of letters, numbers, objects, pictures, or patterns. The things to be compared may be presented at the same time or one after the other. This ability also includes comparing a presented object with a remembered object. | Perceptual Abilities | Cognitive Abilities | -4.021 |
| Perceptual Vision | The ability to see objects or movement of objects to one's side when the eyes are looking ahead. | Visual Abilities | Sensory Abilities | 0 |
| Problem Sensitivity | The ability to tell when something is wrong or is likely to go wrong. It does not involve solving the problem, only recognizing there is a problem. | Idea generation and Reasoning Abilities | Cognitive Abilities | 2.764 |
| Rate Control | The ability to time your movements or the movement of a piece of equipment in anticipation of changes in the speed and/or direction of a moving object or scene. | Control Movement Abilities | Psychomotor Abilities | 5.455 |
| Reaction Time | The ability to quickly respond (with the hand, finger, or foot) to a signal (sound, light, picture) when it appears. | Reaction Time and Speed Abilities | Psychomotor Abilities | 0 |
| Response Orientation | The ability to choose quickly between two or more movements in response to two or more different signals (lights, sounds, pictures). It includes the speed with which the correct response is started with the hand, foot, or other body part. | Control Movement Abilities | Psychomotor Abilities | -3.471 |
| Selective Attention | The ability to concentrate on a task over a period of time without being distracted. | Attentiveness | Cognitive Abilities | 0 |
| Sound Localisation | The ability to tell the direction from which a sound originated. | Auditory and Speech Abilities | Sensory Abilities | 0.339 |
| Spatial Orientation | The ability to know your location in relation to the environment or to know where other objects are in relation to you. | Spatial Abilities | Cognitive Abilities | 1.328 |
| Speech Clarity | The ability to speak clearly so others can understand you. | Auditory and Speech Abilities | Sensory Abilities | 0 |
| Speech Recognition | The ability to identify and understand the speech of another person. | Auditory and Speech Abilities | Sensory Abilities | 0 |
| Speed of Closure | The ability to quickly make sense of, combine, and organize information into meaningful patterns. | Perceptual Abilities | Cognitive Abilities | 5.627 |
| Speed of Limb Movement | The ability to quickly move the arms and legs. | Reaction Time and Speed Abilities | Psychomotor Abilities | 0.991 |
| Stamina | The ability to exert yourself physically over long periods of time without getting winded or out of breath. | Endurance | Physical Abilities | 0.522 |
| Static Strength | The ability to exert maximum muscle force to lift, push, pull, or carry objects. | Physical Strength Abilities | Physical Abilities | 0 |
| Time Sharing | The ability to shift back and forth between two or more activities or sources of information (such as speech, sounds, touch, or other sources). | Attentiveness | Cognitive Abilities | 0 |
| Trunk Strength | The ability to use your abdominal and lower back muscles to support part of the body repeatedly or continuously over time without 'giving out' or fatiguing. | Physical Strength Abilities | Physical Abilities | -0.699 |
| Visual Color Discrimination | The ability to match or detect differences between colors, including shades of color and brightness. | Visual Abilities | Sensory Abilities | 3.271 |
| Visualisation | The ability to imagine how something will look after it is moved around or when its parts are moved or rearranged. | Spatial Abilities | Cognitive Abilities | -0.465 |
| Wrist-Finger Speed | The ability to make fast, simple, repeated movements of the fingers, hands, and wrists. | Reaction Time and Speed Abilities | Psychomotor Abilities | 0 |
| Written Comprehension | The ability to read and understand information and ideas presented in writing. | Verbal Abilities | Cognitive Abilities | 0.621 |
| Written Expression | The ability to communicate information and ideas in writing so others will understand. | Verbal Abilities | Cognitive Abilities | -1.184 |
| R Squared = 0.96 | |  | N= 30684 | |

Table D2 Czech Republic Skills Lasso Analysis

| ONET Item | Description |  |  | Coefficient |
| --- | --- | --- | --- | --- |
| Active Learning | Understanding the implications of new information for both current and future problem-solving and decision-making. | Process | Basic Skills | 0 |
| Active Listening | Giving full attention to what other people are saying, taking time to understand the points being made, asking questions as appropriate, and not interrupting at inappropriate times. | Content | Basic Skills | -2.209 |
| Complex Problem Solving | Identifying complex problems and reviewing related information to develop and evaluate options and implement solutions. | Complex Problem Solving | Cross Functional Skills | -4.862 |
| Coordination | Adjusting actions in relation to others' actions. | Social Skills | Cross Functional Skills | -5.513 |
| Critical Thinking | Using logic and reasoning to identify the strengths and weaknesses of alternative solutions, conclusions or approaches to problems. | Process | Basic Skills | -1.515 |
| Equipment Maintenance | Performing routine maintenance on equipment and determining when and what kind of maintenance is needed. | Technical Skills | Cross Functional Skills | -2.926 |
| Equipment Selection | Determining the kind of tools and equipment needed to do a job. | Technical Skills | Cross Functional Skills | 2.91 |
| Installation | Installing equipment, machines, wiring, or programs to meet specifications. | Technical Skills | Cross Functional Skills | 0 |
| Instructing | Teaching others how to do something. | Social Skills | Cross Functional Skills | -4.382 |
| Judgement and Decision Making | Considering the relative costs and benefits of potential actions to choose the most appropriate one. | Systems Skills | Cross Functional Skills | 4.755 |
| Learning Strategies | Selecting and using training/instructional methods and procedures appropriate for the situation when learning or teaching new things. | Systems Skills | Cross Functional Skills | 3.037 |
| Management of Financial Resources | Determining how money will be spent to get the work done, and accounting for these expenditures. | Resource Management Skills | Cross functional Skills | 0 |
| Management of Material Resources | Obtaining and seeing to the appropriate use of equipment, facilities, and materials needed to do certain work. | Resource Management Skills | Cross functional Skills | 0 |
| Management of Personell Resources | Motivating, developing, and directing people as they work, identifying the best people for the job. | Resource Management Skills | Cross functional Skills | 0.588 |
| Mathematics | Using mathematics to solve problems. | Content | Basic Skills | 0 |
| Monitoring | Monitoring/Assessing performance of yourself, other individuals, or organizations to make improvements or take corrective action. | Process | Basic Skills | -9.976 |
| Negotiation | Bringing others together and trying to reconcile differences. | Social Skills | Cross functional Skills | 0.531 |
| Operation Monitoring | Watching gauges, dials, or other indicators to make sure a machine is working properly. | Technical Skills | Cross Functional Skills | 6.219 |
| Operation and Control | Controlling operations of equipment or systems. | Technical Skills | Cross Functional Skills | -1.811 |
| Operations Analysis | Analyzing needs and product requirements to create a design. | Technical Skills | Cross Functional Skills | 0 |
| Persuasion | Persuading others to change their minds or behavior. | Social Skills | Cross functional Skills | 0 |
| Programming | Writing computer programs for various purposes. | Technical Skills | Cross functional Skills | 0 |
| Quality Control Analysis | Conducting tests and inspections of products, services, or processes to evaluate quality or performance. | Technical Skills | Cross functional Skills | -1.5 |
| Reading Comprehension | Understanding written sentences and paragraphs in work related documents. | Content | Basic Skills | 1.326 |
| Repairing | Repairing machines or systems using the needed tools. | Technical Skills | Cross Functional Skills | 0 |
| Science | Using scientific rules and methods to solve problems. | Content | Basic Skills | 0 |
| Service Orientation | Actively looking for ways to help people. | Social Skills | Cross Functional Skills | 2.727 |
| Social Perceptiveness | Being aware of others' reactions and understanding why they react as they do. | Social Skills | Cross Functional Skills | -2.443 |
| Speaking | Talking to others to convey information effectively. | Content | Basic Skills | 5.244 |
| System Analysis | Determining how a system should work and how changes in conditions, operations, and the environment will affect outcomes. | Systems Skills | Cross Functional Skills | 0.513 |
| System Evaluation | Identifying measures or indicators of system performance and the actions needed to improve or correct performance, relative to the goals of the system. | Systems Skills | Cross Functional Skills | 4.024 |
| Technology Design | Generating or adapting equipment and technology to serve user needs. | Technical Skills | Cross Functional Skills | -5.521 |
| Time Management | Managing one's own time and the time of others. | Resource Management Skills | Cross Functional Skills | 3.495 |
| Troubleshooting | Determining causes of operating errors and deciding what to do about it. | Technical Skills | Cross Functional Skills | 0 |
| Writing | Communicating effectively in writing as appropriate for the needs of the audience. | Content | Basic Skills | 0 |
| R squared = 0.85 |  |  | N= | 30,683 |

Table D3 Czech Republic People, Brains, Brawn Analysis

|  |  |
| --- | --- |
|  | Czech Republic – EU LFS |
| People | -0.023*** |
|  | (0.001) |
| Brains | -0.106*** |
|  | (0.002) |
| Brawn | -0.036*** |
|  | (0.002) |
| People * Brains | 0.000** |
|  | (0.000) |
| People*Brawn | 0.002*** |
|  | (0.000) |
| Brains*Brawn | 0.007*** |
|  | (0.001) |
| N | 30666 |
| R Squared | 11% |

***, **, * denotes significance at the 1%, 5% and 10% levels respectively

S7 Appendix. Individual country Germany

Table E1 Germany Ability Lasso Analysis

| ONET Item | Description |  |  | Coefficient |
| --- | --- | --- | --- | --- |
| Arm-Hand Steadiness | The ability to keep your hand and arm steady while moving your arm or while holding your arm and hand in one position. | Fine manipulative abilities | Psychomotor abilities | -4.602 |
| Auditory Attention | The ability to focus on a single source of sound in the presence of other distracting sounds. | Auditory and speech abilities | Sensory Abilities | 0 |
| Category Flexibility | The ability to generate or use different sets of rules for combining or grouping things in different ways. | Idea Generation and Reasoning Abilities | Cognitive Abilities | -2.962 |
| Control Precision | The ability to quickly and repeatedly adjust the controls of a machine or a vehicle to exact positions. | Control Movement Abilities | Psychomotor Abilities | 2.456 |
| Deductive Reasoning | The ability to apply general rules to specific problems to produce answers that make sense. | Idea Generation and Reasoning Abilities | Cognitive Abilities | -6.096 |
| Depth Perception | The ability to judge which of several objects is closer or farther away from you, or to judge the distance between you and an object. | Visual Abilities | Sensory Abilities | 0.106 |
| Dynamic Flexibility | The ability to quickly and repeatedly bend, stretch, twist, or reach out with your body, arms, and/or legs. | Flexibility, Balance and Coordination | Physical Abilities | 2.566 |
| Dynamic Strength | The ability to exert muscle force repeatedly or continuously over time. This involves muscular endurance and resistance to muscle fatigue. | Physical Strength | Physical Abilities | -1.233 |
| Explosive Strength | The ability to use short bursts of muscle force to propel oneself (as in jumping or sprinting), or to throw an object. | Physical Strength | Physical Abilities | 1.086 |
| Extent Flexibility | The ability to bend, stretch, twist, or reach with your body, arms, and/or legs. | Flexibility, balance and coordination | Physical Abilities | -2.77 |
| Far Vision | The ability to see details at a distance. | Visual Abilities | Sensory Abilities | -3.366 |
| Finger Dexterity | The ability to make precisely coordinated movements of the fingers of one or both hands to grasp, manipulate, or assemble very small objects. | Fine manipulative abilities | Psychomotor abilities | 3.151 |
| Flexibility of Closure | The ability to identify or detect a known pattern (a figure, object, word, or sound) that is hidden in other distracting material. | Perecptual Abilities | Cognitive abilities | 0 |
| Fluency of ideas | The ability to come up with a number of ideas about a topic (the number of ideas is important, not their quality, correctness, or creativity). | Ideas generation and reasoning abilities | Cognitive abilities | -3.635 |
| Glare sensitivity | The ability to see objects in the presence of glare or bright lighting. | Visual abilities | Sensory abilities | -2.578 |
| Gross Body Co-ordination | The ability to coordinate the movement of your arms, legs, and torso together when the whole body is in motion. | Flexibility, Balance and Coordination | Physical Abilities | 0 |
| Gross Body Equilibrium | The ability to keep or regain your body balance or stay upright when in an unstable position. | Flexibility, Balance and Coordination | Physical Abilities | 0 |
| Hearing Sensitivity | The ability to detect or tell the differences between sounds that vary in pitch and loudness. | Auditory and Speech abilities | Sensory Abilities | -1.756 |
| Inductive Reasoning | The ability to combine pieces of information to form general rules or conclusions (includes finding a relationship among seemingly unrelated events). | Idea Generation and Reasoning Abilities | Cognitive Abilities | 0.888 |
| Information Ordering | The ability to arrange things or actions in a certain order or pattern according to a specific rule or set of rules (e.g., patterns of numbers, letters, words, pictures, mathematical operations). | Idea Generation and Reasoning Abilities | Cognitive Abilities | 0 |
| Manual Dexterity | The ability to quickly move your hand, your hand together with your arm, or your two hands to grasp, manipulate, or assemble objects. | Fine Manipulative Abilities | Psychomotor Abilities | 0 |
| Math Reasoning | The ability to choose the right mathematical methods or formulas to solve a problem. | Quantitative Abilities | Cognitive Abilities | 0 |
| Memorization | Abilities related to the recall of available information | Quantitative Abilities | Cognitive Abilities | 0 |
| Multi Limb Co-ordination | The ability to coordinate two or more limbs (for example, two arms, two legs, or one leg and one arm) while sitting, standing, or lying down. It does not involve performing the activities while the whole body is in motion. | Control movement abilities | Psychomotor Abilities | 2.158 |
| Near Vision | The ability to see details at close range (within a few feet of the observer) | Near Vision | Visual Abilities | -2.102 |
| Night Vision | The ability to see under low light conditions | Near Vision | Visual Abilities | 0 |
| Number Facility | The ability to add, subtract, multiply, or divide quickly and correctly. | Quantitative Abilities | Cognitive Abilities | 0 |
| Oral Comprehension | The ability to listen to and understand information and ideas presented through spoken words and sentences. | Verbal Abilities | Cognitive Abilities | 1.498 |
| Oral Expression | The ability to communicate information and ideas in speaking so others will understand. | Verbal Abilities | Cognitive Abilities | 4.621 |
| Originality |  |  |  | 0 |
| Perceptual Speed | The ability to quickly and accurately compare similarities and differences among sets of letters, numbers, objects, pictures, or patterns. The things to be compared may be presented at the same time or one after the other. This ability also includes comparing a presented object with a remembered object. | Perceptual Abilities | Cognitive Abilities | -4.519 |
| Perceptual Vision | The ability to see objects or movement of objects to one's side when the eyes are looking ahead. | Visual Abilities | Sensory Abilities | 1.408 |
| Problem Sensitivity | The ability to tell when something is wrong or is likely to go wrong. It does not involve solving the problem, only recognizing there is a problem. | Idea generation and Reasoning Abilities | Cognitive Abilities | 1.37 |
| Rate Control | The ability to time your movements or the movement of a piece of equipment in anticipation of changes in the speed and/or direction of a moving object or scene. | Control Movement Abilities | Psychomotor Abilities | 2.629 |
| Reaction Time | The ability to quickly respond (with the hand, finger, or foot) to a signal (sound, light, picture) when it appears. | Reaction Time and Speed Abilities | Psychomotor Abilities | 2.536 |
| Response Orientation | The ability to choose quickly between two or more movements in response to two or more different signals (lights, sounds, pictures). It includes the speed with which the correct response is started with the hand, foot, or other body part. | Control Movement Abilities | Psychomotor Abilities | -4.706 |
| Selective Attention | The ability to concentrate on a task over a period of time without being distracted. | Attentiveness | Cognitive Abilities | 0 |
| Sound Localisation | The ability to tell the direction from which a sound originated. | Auditory and Speech Abilities | Sensory Abilities | -1.063 |
| Spatial Orientation | The ability to know your location in relation to the environment or to know where other objects are in relation to you. | Spatial Abilities | Cognitive Abilities | 0.717 |
| Speech Clarity | The ability to speak clearly so others can understand you. | Auditory and Speech Abilities | Sensory Abilities | 0.298 |
| Speech Recognition | The ability to identify and understand the speech of another person. | Auditory and Speech Abilities | Sensory Abilities | -1.706 |
| Speed of Closure | The ability to quickly make sense of, combine, and organize information into meaningful patterns. | Perceptual Abilities | Cognitive Abilities | 5.966 |
| Speed of Limb Movement | The ability to quickly move the arms and legs. | Reaction Time and Speed Abilities | Psychomotor Abilities | 0 |
| Stamina | The ability to exert yourself physically over long periods of time without getting winded or out of breath. | Endurance | Physical Abilities | 0 |
| Static Strength | The ability to exert maximum muscle force to lift, push, pull, or carry objects. | Physical Strength Abilities | Physical Abilities | 0.946 |
| Time Sharing | The ability to shift back and forth between two or more activities or sources of information (such as speech, sounds, touch, or other sources). | Attentiveness | Cognitive Abilities | 2.687 |
| Trunk Strength | The ability to use your abdominal and lower back muscles to support part of the body repeatedly or continuously over time without 'giving out' or fatiguing. | Physical Strength Abilities | Physical Abilities | 0.469 |
| Visual Color Discrimination | The ability to match or detect differences between colors, including shades of color and brightness. | Visual Abilities | Sensory Abilities | 5.545 |
| Visualisation | The ability to imagine how something will look after it is moved around or when its parts are moved or rearranged. | Spatial Abilities | Cognitive Abilities | -0.564 |
| Wrist-Finger Speed | The ability to make fast, simple, repeated movements of the fingers, hands, and wrists. | Reaction Time and Speed Abilities | Psychomotor Abilities | -1.85 |
| Written Comprehension | The ability to read and understand information and ideas presented in writing. | Verbal Abilities | Cognitive Abilities | 0 |
| Written Expression | The ability to communicate information and ideas in writing so others will understand. | Verbal Abilities | Cognitive Abilities | -0.556 |
| R Squared = 0.99 | |  | N= 367032 | |

Table E2 Germany Skills Lasso Analysis

| ONET Item | Description |  |  | Coefficient |
| --- | --- | --- | --- | --- |
| Active Learning | Understanding the implications of new information for both current and future problem-solving and decision-making. | Process | Basic Skills | 0 |
| Active Listening | Giving full attention to what other people are saying, taking time to understand the points being made, asking questions as appropriate, and not interrupting at inappropriate times. | Content | Basic Skills | -1.944 |
| Complex Problem Solving | Identifying complex problems and reviewing related information to develop and evaluate options and implement solutions. | Complex Problem Solving | Cross Functional Skills | -2.644 |
| Coordination | Adjusting actions in relation to others' actions. | Social Skills | Cross Functional Skills | -5.55 |
| Critical Thinking | Using logic and reasoning to identify the strengths and weaknesses of alternative solutions, conclusions or approaches to problems. | Process | Basic Skills | 0 |
| Equipment Maintenance | Performing routine maintenance on equipment and determining when and what kind of maintenance is needed. | Technical Skills | Cross Functional Skills | -0.421 |
| Equipment Selection | Determining the kind of tools and equipment needed to do a job. | Technical Skills | Cross Functional Skills | 2.101 |
| Installation | Installing equipment, machines, wiring, or programs to meet specifications. | Technical Skills | Cross Functional Skills | 0.196 |
| Instructing | Teaching others how to do something. | Social Skills | Cross Functional Skills | -0.497 |
| Judgement and Decision Making | Considering the relative costs and benefits of potential actions to choose the most appropriate one. | Systems Skills | Cross Functional Skills | 5.145 |
| Learning Strategies | Selecting and using training/instructional methods and procedures appropriate for the situation when learning or teaching new things. | Systems Skills | Cross Functional Skills | 0 |
| Management of Financial Resources | Determining how money will be spent to get the work done, and accounting for these expenditures. | Resource Management Skills | Cross functional Skills | 0.628 |
| Management of Material Resources | Obtaining and seeing to the appropriate use of equipment, facilities, and materials needed to do certain work. | Resource Management Skills | Cross functional Skills | -0.614 |
| Management of Personell Resources | Motivating, developing, and directing people as they work, identifying the best people for the job. | Resource Management Skills | Cross functional Skills | 0 |
| Mathematics | Using mathematics to solve problems. | Content | Basic Skills | 0 |
| Monitoring | Monitoring/Assessing performance of yourself, other individuals, or organizations to make improvements or take corrective action. | Process | Basic Skills | -7.947 |
| Negotiation | Bringing others together and trying to reconcile differences. | Social Skills | Cross functional Skills | 2.073 |
| Operation Monitoring | Watching gauges, dials, or other indicators to make sure a machine is working properly. | Technical Skills | Cross Functional Skills | 2.201 |
| Operation and Control | Controlling operations of equipment or systems. | Technical Skills | Cross Functional Skills | 0 |
| Operations Analysis | Analyzing needs and product requirements to create a design. | Technical Skills | Cross Functional Skills | -0.975 |
| Persuasion | Persuading others to change their minds or behavior. | Social Skills | Cross functional Skills | -1.115 |
| Programming | Writing computer programs for various purposes. | Technical Skills | Cross functional Skills | -1.286 |
| Quality Control Analysis | Conducting tests and inspections of products, services, or processes to evaluate quality or performance. | Technical Skills | Cross functional Skills | -0.226 |
| Reading Comprehension | Understanding written sentences and paragraphs in work related documents. | Content | Basic Skills | -2.418 |
| Repairing | Repairing machines or systems using the needed tools. | Technical Skills | Cross Functional Skills | -2.501 |
| Science | Using scientific rules and methods to solve problems. | Content | Basic Skills | 0.529 |
| Service Orientation | Actively looking for ways to help people. | Social Skills | Cross Functional Skills | -0.085 |
| Social Perceptiveness | Being aware of others' reactions and understanding why they react as they do. | Social Skills | Cross Functional Skills | 0 |
| Speaking | Talking to others to convey information effectively. | Content | Basic Skills | 1.879 |
| System Analysis | Determining how a system should work and how changes in conditions, operations, and the environment will affect outcomes. | Systems Skills | Cross Functional Skills | -0.879 |
| System Evaluation | Identifying measures or indicators of system performance and the actions needed to improve or correct performance, relative to the goals of the system. | Systems Skills | Cross Functional Skills | 3.191 |
| Technology Design | Generating or adapting equipment and technology to serve user needs. | Technical Skills | Cross Functional Skills | -2.316 |
| Time Management | Managing one's own time and the time of others. | Resource Management Skills | Cross Functional Skills | 3.221 |
| Troubleshooting | Determining causes of operating errors and deciding what to do about it. | Technical Skills | Cross Functional Skills | 0.789 |
| Writing | Communicating effectively in writing as appropriate for the needs of the audience. | Content | Basic Skills | 4.141 |
| R squared = 0.78 |  |  | N= | 367032 |

Table E3 Germany People, Brains, Brawn Analysis

|  |  |
| --- | --- |
|  | Germany – EU LFS |
| People | -0.008*** |
|  | (0.000) |
| Brains | -0.043*** |
|  | (0.000) |
| Brawn | 0.032*** |
|  | (0.000) |
| People * Brains | -0.006*** |
|  | (0.000) |
| People*Brawn | 0.009*** |
|  | (0.000) |
| Brains*Brawn | -0.000*** |
|  | (0.000) |
| N | 366327 |
| R Squared | 6% |

***, **, * denotes significance at the 1%, 5% and 10% levels respectively

S8 Appendix. Individual country Denmark

Table F1 Denmark Ability Lasso Analysis

| ONET Item | Description |  |  | Coefficient |
| --- | --- | --- | --- | --- |
| Arm-Hand Steadiness | The ability to keep your hand and arm steady while moving your arm or while holding your arm and hand in one position. | Fine manipulative abilities | Psychomotor abilities | -4.013 |
| Auditory Attention | The ability to focus on a single source of sound in the presence of other distracting sounds. | Auditory and speech abilities | Sensory Abilities | 0 |
| Category Flexibility | The ability to generate or use different sets of rules for combining or grouping things in different ways. | Idea Generation and Reasoning Abilities | Cognitive Abilities | -2.185 |
| Control Precision | The ability to quickly and repeatedly adjust the controls of a machine or a vehicle to exact positions. | Control Movement Abilities | Psychomotor Abilities | 1.516 |
| Deductive Reasoning | The ability to apply general rules to specific problems to produce answers that make sense. | Idea Generation and Reasoning Abilities | Cognitive Abilities | -6.421 |
| Depth Perception | The ability to judge which of several objects is closer or farther away from you, or to judge the distance between you and an object. | Visual Abilities | Sensory Abilities | 0.077 |
| Dynamic Flexibility | The ability to quickly and repeatedly bend, stretch, twist, or reach out with your body, arms, and/or legs. | Flexibility, Balance and Coordination | Physical Abilities | 2.487 |
| Dynamic Strength | The ability to exert muscle force repeatedly or continuously over time. This involves muscular endurance and resistance to muscle fatigue. | Physical Strength | Physical Abilities | -3.007 |
| Explosive Strength | The ability to use short bursts of muscle force to propel oneself (as in jumping or sprinting), or to throw an object. | Physical Strength | Physical Abilities | 1.574 |
| Extent Flexibility | The ability to bend, stretch, twist, or reach with your body, arms, and/or legs. | Flexibility, balance and coordination | Physical Abilities | -3.465 |
| Far Vision | The ability to see details at a distance. | Visual Abilities | Sensory Abilities | -4.575 |
| Finger Dexterity | The ability to make precisely coordinated movements of the fingers of one or both hands to grasp, manipulate, or assemble very small objects. | Fine manipulative abilities | Psychomotor abilities | 3.933 |
| Flexibility of Closure | The ability to identify or detect a known pattern (a figure, object, word, or sound) that is hidden in other distracting material. | Perecptual Abilities | Cognitive abilities | -0.436 |
| Fluency of ideas | The ability to come up with a number of ideas about a topic (the number of ideas is important, not their quality, correctness, or creativity). | Ideas generation and reasoning abilities | Cognitive abilities | -5.021 |
| Glare sensitivity | The ability to see objects in the presence of glare or bright lighting. | Visual abilities | Sensory abilities | -2.303 |
| Gross Body Co-ordination | The ability to coordinate the movement of your arms, legs, and torso together when the whole body is in motion. | Flexibility, Balance and Coordination | Physical Abilities | 1.129 |
| Gross Body Equilibrium | The ability to keep or regain your body balance or stay upright when in an unstable position. | Flexibility, Balance and Coordination | Physical Abilities | 0 |
| Hearing Sensitivity | The ability to detect or tell the differences between sounds that vary in pitch and loudness. | Auditory and Speech abilities | Sensory Abilities | -1.832 |
| Inductive Reasoning | The ability to combine pieces of information to form general rules or conclusions (includes finding a relationship among seemingly unrelated events). | Idea Generation and Reasoning Abilities | Cognitive Abilities | 1.618 |
| Information Ordering | The ability to arrange things or actions in a certain order or pattern according to a specific rule or set of rules (e.g., patterns of numbers, letters, words, pictures, mathematical operations). | Idea Generation and Reasoning Abilities | Cognitive Abilities | 0 |
| Manual Dexterity | The ability to quickly move your hand, your hand together with your arm, or your two hands to grasp, manipulate, or assemble objects. | Fine Manipulative Abilities | Psychomotor Abilities | -0.968 |
| Math Reasoning | The ability to choose the right mathematical methods or formulas to solve a problem. | Quantitative Abilities | Cognitive Abilities | 0 |
| Memorization | Abilities related to the recall of available information | Quantitative Abilities | Cognitive Abilities | 0 |
| Multi Limb Co-ordination | The ability to coordinate two or more limbs (for example, two arms, two legs, or one leg and one arm) while sitting, standing, or lying down. It does not involve performing the activities while the whole body is in motion. | Control movement abilities | Psychomotor Abilities | 3.026 |
| Near Vision | The ability to see details at close range (within a few feet of the observer) | Near Vision | Visual Abilities | 0 |
| Night Vision | The ability to see under low light conditions | Near Vision | Visual Abilities | -1.095 |
| Number Facility | The ability to add, subtract, multiply, or divide quickly and correctly. | Quantitative Abilities | Cognitive Abilities | 0 |
| Oral Comprehension | The ability to listen to and understand information and ideas presented through spoken words and sentences. | Verbal Abilities | Cognitive Abilities | 0 |
| Oral Expression | The ability to communicate information and ideas in speaking so others will understand. | Verbal Abilities | Cognitive Abilities | 4.234 |
| Originality |  |  |  | 0.713 |
| Perceptual Speed | The ability to quickly and accurately compare similarities and differences among sets of letters, numbers, objects, pictures, or patterns. The things to be compared may be presented at the same time or one after the other. This ability also includes comparing a presented object with a remembered object. | Perceptual Abilities | Cognitive Abilities | -3.909 |
| Perceptual Vision | The ability to see objects or movement of objects to one's side when the eyes are looking ahead. | Visual Abilities | Sensory Abilities | 1.925 |
| Problem Sensitivity | The ability to tell when something is wrong or is likely to go wrong. It does not involve solving the problem, only recognizing there is a problem. | Idea generation and Reasoning Abilities | Cognitive Abilities | 2.932 |
| Rate Control | The ability to time your movements or the movement of a piece of equipment in anticipation of changes in the speed and/or direction of a moving object or scene. | Control Movement Abilities | Psychomotor Abilities | 4.161 |
| Reaction Time | The ability to quickly respond (with the hand, finger, or foot) to a signal (sound, light, picture) when it appears. | Reaction Time and Speed Abilities | Psychomotor Abilities | 0 |
| Response Orientation | The ability to choose quickly between two or more movements in response to two or more different signals (lights, sounds, pictures). It includes the speed with which the correct response is started with the hand, foot, or other body part. | Control Movement Abilities | Psychomotor Abilities | -4.639 |
| Selective Attention | The ability to concentrate on a task over a period of time without being distracted. | Attentiveness | Cognitive Abilities | 0 |
| Sound Localisation | The ability to tell the direction from which a sound originated. | Auditory and Speech Abilities | Sensory Abilities | 0 |
| Spatial Orientation | The ability to know your location in relation to the environment or to know where other objects are in relation to you. | Spatial Abilities | Cognitive Abilities | 1.981 |
| Speech Clarity | The ability to speak clearly so others can understand you. | Auditory and Speech Abilities | Sensory Abilities | 1.652 |
| Speech Recognition | The ability to identify and understand the speech of another person. | Auditory and Speech Abilities | Sensory Abilities | -1.95 |
| Speed of Closure | The ability to quickly make sense of, combine, and organize information into meaningful patterns. | Perceptual Abilities | Cognitive Abilities | 6.196 |
| Speed of Limb Movement | The ability to quickly move the arms and legs. | Reaction Time and Speed Abilities | Psychomotor Abilities | 0.944 |
| Stamina | The ability to exert yourself physically over long periods of time without getting winded or out of breath. | Endurance | Physical Abilities | 0.326 |
| Static Strength | The ability to exert maximum muscle force to lift, push, pull, or carry objects. | Physical Strength Abilities | Physical Abilities | 0.915 |
| Time Sharing | The ability to shift back and forth between two or more activities or sources of information (such as speech, sounds, touch, or other sources). | Attentiveness | Cognitive Abilities | 0 |
| Trunk Strength | The ability to use your abdominal and lower back muscles to support part of the body repeatedly or continuously over time without 'giving out' or fatiguing. | Physical Strength Abilities | Physical Abilities | 0.747 |
| Visual Color Discrimination | The ability to match or detect differences between colors, including shades of color and brightness. | Visual Abilities | Sensory Abilities | 5.791 |
| Visualisation | The ability to imagine how something will look after it is moved around or when its parts are moved or rearranged. | Spatial Abilities | Cognitive Abilities | -0.98 |
| Wrist-Finger Speed | The ability to make fast, simple, repeated movements of the fingers, hands, and wrists. | Reaction Time and Speed Abilities | Psychomotor Abilities | -1.325 |
| Written Comprehension | The ability to read and understand information and ideas presented in writing. | Verbal Abilities | Cognitive Abilities | 0 |
| Written Expression | The ability to communicate information and ideas in writing so others will understand. | Verbal Abilities | Cognitive Abilities | -1.281 |
| R Squared = 0.97 | |  | N= 122324 | |

Table F2 Denmark Skills Lasso Analysis

| ONET Item | Description |  |  | Coefficient |
| --- | --- | --- | --- | --- |
| Active Learning | Understanding the implications of new information for both current and future problem-solving and decision-making. | Process | Basic Skills | 0.244 |
| Active Listening | Giving full attention to what other people are saying, taking time to understand the points being made, asking questions as appropriate, and not interrupting at inappropriate times. | Content | Basic Skills | -0.785 |
| Complex Problem Solving | Identifying complex problems and reviewing related information to develop and evaluate options and implement solutions. | Complex Problem Solving | Cross Functional Skills | -4.186 |
| Coordination | Adjusting actions in relation to others' actions. | Social Skills | Cross Functional Skills | -4.075 |
| Critical Thinking | Using logic and reasoning to identify the strengths and weaknesses of alternative solutions, conclusions or approaches to problems. | Process | Basic Skills | 0 |
| Equipment Maintenance | Performing routine maintenance on equipment and determining when and what kind of maintenance is needed. | Technical Skills | Cross Functional Skills | 0 |
| Equipment Selection | Determining the kind of tools and equipment needed to do a job. | Technical Skills | Cross Functional Skills | 0.422 |
| Installation | Installing equipment, machines, wiring, or programs to meet specifications. | Technical Skills | Cross Functional Skills | 0 |
| Instructing | Teaching others how to do something. | Social Skills | Cross Functional Skills | -2.398 |
| Judgement and Decision Making | Considering the relative costs and benefits of potential actions to choose the most appropriate one. | Systems Skills | Cross Functional Skills | 4.495 |
| Learning Strategies | Selecting and using training/instructional methods and procedures appropriate for the situation when learning or teaching new things. | Systems Skills | Cross Functional Skills | 0.608 |
| Management of Financial Resources | Determining how money will be spent to get the work done, and accounting for these expenditures. | Resource Management Skills | Cross functional Skills | 0.333 |
| Management of Material Resources | Obtaining and seeing to the appropriate use of equipment, facilities, and materials needed to do certain work. | Resource Management Skills | Cross functional Skills | 0 |
| Management of Personell Resources | Motivating, developing, and directing people as they work, identifying the best people for the job. | Resource Management Skills | Cross functional Skills | 1.581 |
| Mathematics | Using mathematics to solve problems. | Content | Basic Skills | 0 |
| Monitoring | Monitoring/Assessing performance of yourself, other individuals, or organizations to make improvements or take corrective action. | Process | Basic Skills | -6.518 |
| Negotiation | Bringing others together and trying to reconcile differences. | Social Skills | Cross functional Skills | 0.971 |
| Operation Monitoring | Watching gauges, dials, or other indicators to make sure a machine is working properly. | Technical Skills | Cross Functional Skills | 2.406 |
| Operation and Control | Controlling operations of equipment or systems. | Technical Skills | Cross Functional Skills | -0.731 |
| Operations Analysis | Analyzing needs and product requirements to create a design. | Technical Skills | Cross Functional Skills | -0.913 |
| Persuasion | Persuading others to change their minds or behavior. | Social Skills | Cross functional Skills | -1.933 |
| Programming | Writing computer programs for various purposes. | Technical Skills | Cross functional Skills | -0.781 |
| Quality Control Analysis | Conducting tests and inspections of products, services, or processes to evaluate quality or performance. | Technical Skills | Cross functional Skills | -0.843 |
| Reading Comprehension | Understanding written sentences and paragraphs in work related documents. | Content | Basic Skills | 0 |
| Repairing | Repairing machines or systems using the needed tools. | Technical Skills | Cross Functional Skills | -1.338 |
| Science | Using scientific rules and methods to solve problems. | Content | Basic Skills | 0.659 |
| Service Orientation | Actively looking for ways to help people. | Social Skills | Cross Functional Skills | -0.389 |
| Social Perceptiveness | Being aware of others' reactions and understanding why they react as they do. | Social Skills | Cross Functional Skills | 0 |
| Speaking | Talking to others to convey information effectively. | Content | Basic Skills | 2.193 |
| System Analysis | Determining how a system should work and how changes in conditions, operations, and the environment will affect outcomes. | Systems Skills | Cross Functional Skills | -1.55 |
| System Evaluation | Identifying measures or indicators of system performance and the actions needed to improve or correct performance, relative to the goals of the system. | Systems Skills | Cross Functional Skills | 3.743 |
| Technology Design | Generating or adapting equipment and technology to serve user needs. | Technical Skills | Cross Functional Skills | -1.921 |
| Time Management | Managing one's own time and the time of others. | Resource Management Skills | Cross Functional Skills | 2.396 |
| Troubleshooting | Determining causes of operating errors and deciding what to do about it. | Technical Skills | Cross Functional Skills | 0.832 |
| Writing | Communicating effectively in writing as appropriate for the needs of the audience. | Content | Basic Skills | 1.263 |
| R squared = 0.73 |  |  | N= | 122324 |

Table F Denmark People, Brains, Brawn Analysis

|  |  |
| --- | --- |
|  | Denmark – EU LFS |
| People | -0.007*** |
|  | (0.000) |
| Brains | -0.046*** |
|  | (0.001) |
| Brawn | 0.014*** |
|  | (0.001) |
| People * Brains | -0.001*** |
|  | (0.000) |
| People*Brawn | 0.001*** |
|  | (0.000) |
| Brains*Brawn | 0.000*** |
|  | (0.000) |
| N | 122137 |
| R Squared | 7% |

***, **, * denotes significance at the 1%, 5% and 10% levels respectively

S9 Appendix. Individual country Estonia

Table G1 Estonia Ability Lasso Analysis

| ONET Item | Description |  |  | Coefficient |
| --- | --- | --- | --- | --- |
| Arm-Hand Steadiness | The ability to keep your hand and arm steady while moving your arm or while holding your arm and hand in one position. | Fine manipulative abilities | Psychomotor abilities | -3.599 |
| Auditory Attention | The ability to focus on a single source of sound in the presence of other distracting sounds. | Auditory and speech abilities | Sensory Abilities | 0.794 |
| Category Flexibility | The ability to generate or use different sets of rules for combining or grouping things in different ways. | Idea Generation and Reasoning Abilities | Cognitive Abilities | 0 |
| Control Precision | The ability to quickly and repeatedly adjust the controls of a machine or a vehicle to exact positions. | Control Movement Abilities | Psychomotor Abilities | 2.644 |
| Deductive Reasoning | The ability to apply general rules to specific problems to produce answers that make sense. | Idea Generation and Reasoning Abilities | Cognitive Abilities | -8.479 |
| Depth Perception | The ability to judge which of several objects is closer or farther away from you, or to judge the distance between you and an object. | Visual Abilities | Sensory Abilities | -1.647 |
| Dynamic Flexibility | The ability to quickly and repeatedly bend, stretch, twist, or reach out with your body, arms, and/or legs. | Flexibility, Balance and Coordination | Physical Abilities | 6.82 |
| Dynamic Strength | The ability to exert muscle force repeatedly or continuously over time. This involves muscular endurance and resistance to muscle fatigue. | Physical Strength | Physical Abilities | -1.164 |
| Explosive Strength | The ability to use short bursts of muscle force to propel oneself (as in jumping or sprinting), or to throw an object. | Physical Strength | Physical Abilities | 0.543 |
| Extent Flexibility | The ability to bend, stretch, twist, or reach with your body, arms, and/or legs. | Flexibility, balance and coordination | Physical Abilities | -2.285 |
| Far Vision | The ability to see details at a distance. | Visual Abilities | Sensory Abilities | -3.274 |
| Finger Dexterity | The ability to make precisely coordinated movements of the fingers of one or both hands to grasp, manipulate, or assemble very small objects. | Fine manipulative abilities | Psychomotor abilities | 0.988 |
| Flexibility of Closure | The ability to identify or detect a known pattern (a figure, object, word, or sound) that is hidden in other distracting material. | Perecptual Abilities | Cognitive abilities | 0.526 |
| Fluency of ideas | The ability to come up with a number of ideas about a topic (the number of ideas is important, not their quality, correctness, or creativity). | Ideas generation and reasoning abilities | Cognitive abilities | -5.548 |
| Glare sensitivity | The ability to see objects in the presence of glare or bright lighting. | Visual abilities | Sensory abilities | -1.489 |
| Gross Body Co-ordination | The ability to coordinate the movement of your arms, legs, and torso together when the whole body is in motion. | Flexibility, Balance and Coordination | Physical Abilities | 1.37 |
| Gross Body Equilibrium | The ability to keep or regain your body balance or stay upright when in an unstable position. | Flexibility, Balance and Coordination | Physical Abilities | 0 |
| Hearing Sensitivity | The ability to detect or tell the differences between sounds that vary in pitch and loudness. | Auditory and Speech abilities | Sensory Abilities | 0 |
| Inductive Reasoning | The ability to combine pieces of information to form general rules or conclusions (includes finding a relationship among seemingly unrelated events). | Idea Generation and Reasoning Abilities | Cognitive Abilities | 0 |
| Information Ordering | The ability to arrange things or actions in a certain order or pattern according to a specific rule or set of rules (e.g., patterns of numbers, letters, words, pictures, mathematical operations). | Idea Generation and Reasoning Abilities | Cognitive Abilities | -2.993 |
| Manual Dexterity | The ability to quickly move your hand, your hand together with your arm, or your two hands to grasp, manipulate, or assemble objects. | Fine Manipulative Abilities | Psychomotor Abilities | 0 |
| Math Reasoning | The ability to choose the right mathematical methods or formulas to solve a problem. | Quantitative Abilities | Cognitive Abilities | 1.922 |
| Memorization | Abilities related to the recall of available information | Quantitative Abilities | Cognitive Abilities | 0.016 |
| Multi Limb Co-ordination | The ability to coordinate two or more limbs (for example, two arms, two legs, or one leg and one arm) while sitting, standing, or lying down. It does not involve performing the activities while the whole body is in motion. | Control movement abilities | Psychomotor Abilities | 0 |
| Near Vision | The ability to see details at close range (within a few feet of the observer) | Near Vision | Visual Abilities | 0 |
| Night Vision | The ability to see under low light conditions | Near Vision | Visual Abilities | -0.298 |
| Number Facility | The ability to add, subtract, multiply, or divide quickly and correctly. | Quantitative Abilities | Cognitive Abilities | -0.371 |
| Oral Comprehension | The ability to listen to and understand information and ideas presented through spoken words and sentences. | Verbal Abilities | Cognitive Abilities | 1.838 |
| Oral Expression | The ability to communicate information and ideas in speaking so others will understand. | Verbal Abilities | Cognitive Abilities | 2.239 |
| Originality |  |  |  | 1.124 |
| Perceptual Speed | The ability to quickly and accurately compare similarities and differences among sets of letters, numbers, objects, pictures, or patterns. The things to be compared may be presented at the same time or one after the other. This ability also includes comparing a presented object with a remembered object. | Perceptual Abilities | Cognitive Abilities | -6.342 |
| Perceptual Vision | The ability to see objects or movement of objects to one's side when the eyes are looking ahead. | Visual Abilities | Sensory Abilities | 0.947 |
| Problem Sensitivity | The ability to tell when something is wrong or is likely to go wrong. It does not involve solving the problem, only recognizing there is a problem. | Idea generation and Reasoning Abilities | Cognitive Abilities | 3.415 |
| Rate Control | The ability to time your movements or the movement of a piece of equipment in anticipation of changes in the speed and/or direction of a moving object or scene. | Control Movement Abilities | Psychomotor Abilities | 2.162 |
| Reaction Time | The ability to quickly respond (with the hand, finger, or foot) to a signal (sound, light, picture) when it appears. | Reaction Time and Speed Abilities | Psychomotor Abilities | 0 |
| Response Orientation | The ability to choose quickly between two or more movements in response to two or more different signals (lights, sounds, pictures). It includes the speed with which the correct response is started with the hand, foot, or other body part. | Control Movement Abilities | Psychomotor Abilities | 0 |
| Selective Attention | The ability to concentrate on a task over a period of time without being distracted. | Attentiveness | Cognitive Abilities | 0 |
| Sound Localisation | The ability to tell the direction from which a sound originated. | Auditory and Speech Abilities | Sensory Abilities | -1.219 |
| Spatial Orientation | The ability to know your location in relation to the environment or to know where other objects are in relation to you. | Spatial Abilities | Cognitive Abilities | 0.892 |
| Speech Clarity | The ability to speak clearly so others can understand you. | Auditory and Speech Abilities | Sensory Abilities | 0.761 |
| Speech Recognition | The ability to identify and understand the speech of another person. | Auditory and Speech Abilities | Sensory Abilities | 0 |
| Speed of Closure | The ability to quickly make sense of, combine, and organize information into meaningful patterns. | Perceptual Abilities | Cognitive Abilities | 5.895 |
| Speed of Limb Movement | The ability to quickly move the arms and legs. | Reaction Time and Speed Abilities | Psychomotor Abilities | 0 |
| Stamina | The ability to exert yourself physically over long periods of time without getting winded or out of breath. | Endurance | Physical Abilities | 0 |
| Static Strength | The ability to exert maximum muscle force to lift, push, pull, or carry objects. | Physical Strength Abilities | Physical Abilities | 0 |
| Time Sharing | The ability to shift back and forth between two or more activities or sources of information (such as speech, sounds, touch, or other sources). | Attentiveness | Cognitive Abilities | 1.877 |
| Trunk Strength | The ability to use your abdominal and lower back muscles to support part of the body repeatedly or continuously over time without 'giving out' or fatiguing. | Physical Strength Abilities | Physical Abilities | -0.773 |
| Visual Color Discrimination | The ability to match or detect differences between colors, including shades of color and brightness. | Visual Abilities | Sensory Abilities | 4.178 |
| Visualisation | The ability to imagine how something will look after it is moved around or when its parts are moved or rearranged. | Spatial Abilities | Cognitive Abilities | 0.405 |
| Wrist-Finger Speed | The ability to make fast, simple, repeated movements of the fingers, hands, and wrists. | Reaction Time and Speed Abilities | Psychomotor Abilities | 0 |
| Written Comprehension | The ability to read and understand information and ideas presented in writing. | Verbal Abilities | Cognitive Abilities | 0 |
| Written Expression | The ability to communicate information and ideas in writing so others will understand. | Verbal Abilities | Cognitive Abilities | 0 |
| R Squared = 0.97 | |  | N= 21645 | |

Table G2 Estonia Skills Lasso Analysis

| ONET Item | Description |  |  | Coefficient |
| --- | --- | --- | --- | --- |
| Active Learning | Understanding the implications of new information for both current and future problem-solving and decision-making. | Process | Basic Skills | 0 |
| Active Listening | Giving full attention to what other people are saying, taking time to understand the points being made, asking questions as appropriate, and not interrupting at inappropriate times. | Content | Basic Skills | -0.771 |
| Complex Problem Solving | Identifying complex problems and reviewing related information to develop and evaluate options and implement solutions. | Complex Problem Solving | Cross Functional Skills | 0 |
| Coordination | Adjusting actions in relation to others' actions. | Social Skills | Cross Functional Skills | -4.317 |
| Critical Thinking | Using logic and reasoning to identify the strengths and weaknesses of alternative solutions, conclusions or approaches to problems. | Process | Basic Skills | -2.277 |
| Equipment Maintenance | Performing routine maintenance on equipment and determining when and what kind of maintenance is needed. | Technical Skills | Cross Functional Skills | -0.188 |
| Equipment Selection | Determining the kind of tools and equipment needed to do a job. | Technical Skills | Cross Functional Skills | 1.296 |
| Installation | Installing equipment, machines, wiring, or programs to meet specifications. | Technical Skills | Cross Functional Skills | 0.214 |
| Instructing | Teaching others how to do something. | Social Skills | Cross Functional Skills | -3.306 |
| Judgement and Decision Making | Considering the relative costs and benefits of potential actions to choose the most appropriate one. | Systems Skills | Cross Functional Skills | 1.497 |
| Learning Strategies | Selecting and using training/instructional methods and procedures appropriate for the situation when learning or teaching new things. | Systems Skills | Cross Functional Skills | 1.179 |
| Management of Financial Resources | Determining how money will be spent to get the work done, and accounting for these expenditures. | Resource Management Skills | Cross functional Skills | 0 |
| Management of Material Resources | Obtaining and seeing to the appropriate use of equipment, facilities, and materials needed to do certain work. | Resource Management Skills | Cross functional Skills | -0.505 |
| Management of Personell Resources | Motivating, developing, and directing people as they work, identifying the best people for the job. | Resource Management Skills | Cross functional Skills | 0 |
| Mathematics | Using mathematics to solve problems. | Content | Basic Skills | 0 |
| Monitoring | Monitoring/Assessing performance of yourself, other individuals, or organizations to make improvements or take corrective action. | Process | Basic Skills | -5.397 |
| Negotiation | Bringing others together and trying to reconcile differences. | Social Skills | Cross functional Skills | 1.887 |
| Operation Monitoring | Watching gauges, dials, or other indicators to make sure a machine is working properly. | Technical Skills | Cross Functional Skills | 2.895 |
| Operation and Control | Controlling operations of equipment or systems. | Technical Skills | Cross Functional Skills | -0.305 |
| Operations Analysis | Analyzing needs and product requirements to create a design. | Technical Skills | Cross Functional Skills | -1.081 |
| Persuasion | Persuading others to change their minds or behavior. | Social Skills | Cross functional Skills | 0 |
| Programming | Writing computer programs for various purposes. | Technical Skills | Cross functional Skills | 0 |
| Quality Control Analysis | Conducting tests and inspections of products, services, or processes to evaluate quality or performance. | Technical Skills | Cross functional Skills | -0.953 |
| Reading Comprehension | Understanding written sentences and paragraphs in work related documents. | Content | Basic Skills | 0 |
| Repairing | Repairing machines or systems using the needed tools. | Technical Skills | Cross Functional Skills | -1.489 |
| Science | Using scientific rules and methods to solve problems. | Content | Basic Skills | 0.506 |
| Service Orientation | Actively looking for ways to help people. | Social Skills | Cross Functional Skills | 0 |
| Social Perceptiveness | Being aware of others' reactions and understanding why they react as they do. | Social Skills | Cross Functional Skills | 0 |
| Speaking | Talking to others to convey information effectively. | Content | Basic Skills | 0 |
| System Analysis | Determining how a system should work and how changes in conditions, operations, and the environment will affect outcomes. | Systems Skills | Cross Functional Skills | 0 |
| System Evaluation | Identifying measures or indicators of system performance and the actions needed to improve or correct performance, relative to the goals of the system. | Systems Skills | Cross Functional Skills | 3.661 |
| Technology Design | Generating or adapting equipment and technology to serve user needs. | Technical Skills | Cross Functional Skills | -3.409 |
| Time Management | Managing one's own time and the time of others. | Resource Management Skills | Cross Functional Skills | 3.43 |
| Troubleshooting | Determining causes of operating errors and deciding what to do about it. | Technical Skills | Cross Functional Skills | 0 |
| Writing | Communicating effectively in writing as appropriate for the needs of the audience. | Content | Basic Skills | 2.243 |
| R squared = 0.85 |  |  | N= | 21645 |

Table G3 Estonia People, Brains, Brawn Analysis

|  |  |
| --- | --- |
|  | Estonia – EU LFS |
| People | -0.019*** |
|  | (0.001) |
| Brains | -0.175*** |
|  | (0.002) |
| Brawn | -0.009*** |
|  | (0.002) |
| People * Brains | 0.018*** |
|  | (0.001) |
| People*Brawn | 0.002*** |
|  | (0.000) |
| Brains*Brawn | -0.030*** |
|  | (0.001) |
| N | 21615 |
| R Squared | 22% |

***, **, * denotes significance at the 1%, 5% and 10% levels respectively

S10 Appendix. Individual country Spain

Table H1 Spain Ability Lasso Analysis

| ONET Item | Description |  |  | Coefficient |
| --- | --- | --- | --- | --- |
| Arm-Hand Steadiness | The ability to keep your hand and arm steady while moving your arm or while holding your arm and hand in one position. | Fine manipulative abilities | Psychomotor abilities | -4.435 |
| Auditory Attention | The ability to focus on a single source of sound in the presence of other distracting sounds. | Auditory and speech abilities | Sensory Abilities | 0 |
| Category Flexibility | The ability to generate or use different sets of rules for combining or grouping things in different ways. | Idea Generation and Reasoning Abilities | Cognitive Abilities | -2.173 |
| Control Precision | The ability to quickly and repeatedly adjust the controls of a machine or a vehicle to exact positions. | Control Movement Abilities | Psychomotor Abilities | 2.781 |
| Deductive Reasoning | The ability to apply general rules to specific problems to produce answers that make sense. | Idea Generation and Reasoning Abilities | Cognitive Abilities | -5.576 |
| Depth Perception | The ability to judge which of several objects is closer or farther away from you, or to judge the distance between you and an object. | Visual Abilities | Sensory Abilities | 0.728 |
| Dynamic Flexibility | The ability to quickly and repeatedly bend, stretch, twist, or reach out with your body, arms, and/or legs. | Flexibility, Balance and Coordination | Physical Abilities | 2.732 |
| Dynamic Strength | The ability to exert muscle force repeatedly or continuously over time. This involves muscular endurance and resistance to muscle fatigue. | Physical Strength | Physical Abilities | -0.619 |
| Explosive Strength | The ability to use short bursts of muscle force to propel oneself (as in jumping or sprinting), or to throw an object. | Physical Strength | Physical Abilities | 0.267 |
| Extent Flexibility | The ability to bend, stretch, twist, or reach with your body, arms, and/or legs. | Flexibility, balance and coordination | Physical Abilities | -1.675 |
| Far Vision | The ability to see details at a distance. | Visual Abilities | Sensory Abilities | -2.498 |
| Finger Dexterity | The ability to make precisely coordinated movements of the fingers of one or both hands to grasp, manipulate, or assemble very small objects. | Fine manipulative abilities | Psychomotor abilities | 2.178 |
| Flexibility of Closure | The ability to identify or detect a known pattern (a figure, object, word, or sound) that is hidden in other distracting material. | Perecptual Abilities | Cognitive abilities | 0 |
| Fluency of ideas | The ability to come up with a number of ideas about a topic (the number of ideas is important, not their quality, correctness, or creativity). | Ideas generation and reasoning abilities | Cognitive abilities | -3.008 |
| Glare sensitivity | The ability to see objects in the presence of glare or bright lighting. | Visual abilities | Sensory abilities | -2.323 |
| Gross Body Co-ordination | The ability to coordinate the movement of your arms, legs, and torso together when the whole body is in motion. | Flexibility, Balance and Coordination | Physical Abilities | 0 |
| Gross Body Equilibrium | The ability to keep or regain your body balance or stay upright when in an unstable position. | Flexibility, Balance and Coordination | Physical Abilities | 0 |
| Hearing Sensitivity | The ability to detect or tell the differences between sounds that vary in pitch and loudness. | Auditory and Speech abilities | Sensory Abilities | -0.514 |
| Inductive Reasoning | The ability to combine pieces of information to form general rules or conclusions (includes finding a relationship among seemingly unrelated events). | Idea Generation and Reasoning Abilities | Cognitive Abilities | 0 |
| Information Ordering | The ability to arrange things or actions in a certain order or pattern according to a specific rule or set of rules (e.g., patterns of numbers, letters, words, pictures, mathematical operations). | Idea Generation and Reasoning Abilities | Cognitive Abilities | 0 |
| Manual Dexterity | The ability to quickly move your hand, your hand together with your arm, or your two hands to grasp, manipulate, or assemble objects. | Fine Manipulative Abilities | Psychomotor Abilities | 0 |
| Math Reasoning | The ability to choose the right mathematical methods or formulas to solve a problem. | Quantitative Abilities | Cognitive Abilities | 0 |
| Memorization | Abilities related to the recall of available information | Quantitative Abilities | Cognitive Abilities | 0 |
| Multi Limb Co-ordination | The ability to coordinate two or more limbs (for example, two arms, two legs, or one leg and one arm) while sitting, standing, or lying down. It does not involve performing the activities while the whole body is in motion. | Control movement abilities | Psychomotor Abilities | 1.02 |
| Near Vision | The ability to see details at close range (within a few feet of the observer) | Near Vision | Visual Abilities | 0 |
| Night Vision | The ability to see under low light conditions | Near Vision | Visual Abilities | 0 |
| Number Facility | The ability to add, subtract, multiply, or divide quickly and correctly. | Quantitative Abilities | Cognitive Abilities | 0 |
| Oral Comprehension | The ability to listen to and understand information and ideas presented through spoken words and sentences. | Verbal Abilities | Cognitive Abilities | 0 |
| Oral Expression | The ability to communicate information and ideas in speaking so others will understand. | Verbal Abilities | Cognitive Abilities | 3.015 |
| Originality |  |  |  | 0 |
| Perceptual Speed | The ability to quickly and accurately compare similarities and differences among sets of letters, numbers, objects, pictures, or patterns. The things to be compared may be presented at the same time or one after the other. This ability also includes comparing a presented object with a remembered object. | Perceptual Abilities | Cognitive Abilities | -5.982 |
| Perceptual Vision | The ability to see objects or movement of objects to one's side when the eyes are looking ahead. | Visual Abilities | Sensory Abilities | 3.045 |
| Problem Sensitivity | The ability to tell when something is wrong or is likely to go wrong. It does not involve solving the problem, only recognizing there is a problem. | Idea generation and Reasoning Abilities | Cognitive Abilities | 2.671 |
| Rate Control | The ability to time your movements or the movement of a piece of equipment in anticipation of changes in the speed and/or direction of a moving object or scene. | Control Movement Abilities | Psychomotor Abilities | 1.954 |
| Reaction Time | The ability to quickly respond (with the hand, finger, or foot) to a signal (sound, light, picture) when it appears. | Reaction Time and Speed Abilities | Psychomotor Abilities | 3.098 |
| Response Orientation | The ability to choose quickly between two or more movements in response to two or more different signals (lights, sounds, pictures). It includes the speed with which the correct response is started with the hand, foot, or other body part. | Control Movement Abilities | Psychomotor Abilities | -4.497 |
| Selective Attention | The ability to concentrate on a task over a period of time without being distracted. | Attentiveness | Cognitive Abilities | 0 |
| Sound Localisation | The ability to tell the direction from which a sound originated. | Auditory and Speech Abilities | Sensory Abilities | -2.565 |
| Spatial Orientation | The ability to know your location in relation to the environment or to know where other objects are in relation to you. | Spatial Abilities | Cognitive Abilities | 0.275 |
| Speech Clarity | The ability to speak clearly so others can understand you. | Auditory and Speech Abilities | Sensory Abilities | 1.529 |
| Speech Recognition | The ability to identify and understand the speech of another person. | Auditory and Speech Abilities | Sensory Abilities | -3.306 |
| Speed of Closure | The ability to quickly make sense of, combine, and organize information into meaningful patterns. | Perceptual Abilities | Cognitive Abilities | 5.823 |
| Speed of Limb Movement | The ability to quickly move the arms and legs. | Reaction Time and Speed Abilities | Psychomotor Abilities | -1.424 |
| Stamina | The ability to exert yourself physically over long periods of time without getting winded or out of breath. | Endurance | Physical Abilities | 0.878 |
| Static Strength | The ability to exert maximum muscle force to lift, push, pull, or carry objects. | Physical Strength Abilities | Physical Abilities | 0.145 |
| Time Sharing | The ability to shift back and forth between two or more activities or sources of information (such as speech, sounds, touch, or other sources). | Attentiveness | Cognitive Abilities | 3.203 |
| Trunk Strength | The ability to use your abdominal and lower back muscles to support part of the body repeatedly or continuously over time without 'giving out' or fatiguing. | Physical Strength Abilities | Physical Abilities | 0.036 |
| Visual Color Discrimination | The ability to match or detect differences between colors, including shades of color and brightness. | Visual Abilities | Sensory Abilities | 5.341 |
| Visualisation | The ability to imagine how something will look after it is moved around or when its parts are moved or rearranged. | Spatial Abilities | Cognitive Abilities | -1.33 |
| Wrist-Finger Speed | The ability to make fast, simple, repeated movements of the fingers, hands, and wrists. | Reaction Time and Speed Abilities | Psychomotor Abilities | -1.56 |
| Written Comprehension | The ability to read and understand information and ideas presented in writing. | Verbal Abilities | Cognitive Abilities | 0 |
| Written Expression | The ability to communicate information and ideas in writing so others will understand. | Verbal Abilities | Cognitive Abilities | -0.446 |
| R Squared = 0.99 | |  | N= 75418 | |

Table H2 Spain Skills Lasso Analysis

| ONET Item | Description |  |  | Coefficient |
| --- | --- | --- | --- | --- |
| Active Learning | Understanding the implications of new information for both current and future problem-solving and decision-making. | Process | Basic Skills | 0 |
| Active Listening | Giving full attention to what other people are saying, taking time to understand the points being made, asking questions as appropriate, and not interrupting at inappropriate times. | Content | Basic Skills | -1.125 |
| Complex Problem Solving | Identifying complex problems and reviewing related information to develop and evaluate options and implement solutions. | Complex Problem Solving | Cross Functional Skills | -3.327 |
| Coordination | Adjusting actions in relation to others' actions. | Social Skills | Cross Functional Skills | -6.541 |
| Critical Thinking | Using logic and reasoning to identify the strengths and weaknesses of alternative solutions, conclusions or approaches to problems. | Process | Basic Skills | -2.52 |
| Equipment Maintenance | Performing routine maintenance on equipment and determining when and what kind of maintenance is needed. | Technical Skills | Cross Functional Skills | -1.578 |
| Equipment Selection | Determining the kind of tools and equipment needed to do a job. | Technical Skills | Cross Functional Skills | 3.176 |
| Installation | Installing equipment, machines, wiring, or programs to meet specifications. | Technical Skills | Cross Functional Skills | 0.905 |
| Instructing | Teaching others how to do something. | Social Skills | Cross Functional Skills | -4.126 |
| Judgement and Decision Making | Considering the relative costs and benefits of potential actions to choose the most appropriate one. | Systems Skills | Cross Functional Skills | 4.165 |
| Learning Strategies | Selecting and using training/instructional methods and procedures appropriate for the situation when learning or teaching new things. | Systems Skills | Cross Functional Skills | 1.779 |
| Management of Financial Resources | Determining how money will be spent to get the work done, and accounting for these expenditures. | Resource Management Skills | Cross functional Skills | 0 |
| Management of Material Resources | Obtaining and seeing to the appropriate use of equipment, facilities, and materials needed to do certain work. | Resource Management Skills | Cross functional Skills | -0.655 |
| Management of Personell Resources | Motivating, developing, and directing people as they work, identifying the best people for the job. | Resource Management Skills | Cross functional Skills | 1.042 |
| Mathematics | Using mathematics to solve problems. | Content | Basic Skills | 0 |
| Monitoring | Monitoring/Assessing performance of yourself, other individuals, or organizations to make improvements or take corrective action. | Process | Basic Skills | -8.229 |
| Negotiation | Bringing others together and trying to reconcile differences. | Social Skills | Cross functional Skills | 1.016 |
| Operation Monitoring | Watching gauges, dials, or other indicators to make sure a machine is working properly. | Technical Skills | Cross Functional Skills | 4.51 |
| Operation and Control | Controlling operations of equipment or systems. | Technical Skills | Cross Functional Skills | -1.505 |
| Operations Analysis | Analyzing needs and product requirements to create a design. | Technical Skills | Cross Functional Skills | -0.747 |
| Persuasion | Persuading others to change their minds or behavior. | Social Skills | Cross functional Skills | 0 |
| Programming | Writing computer programs for various purposes. | Technical Skills | Cross functional Skills | 0 |
| Quality Control Analysis | Conducting tests and inspections of products, services, or processes to evaluate quality or performance. | Technical Skills | Cross functional Skills | -0.749 |
| Reading Comprehension | Understanding written sentences and paragraphs in work related documents. | Content | Basic Skills | 0 |
| Repairing | Repairing machines or systems using the needed tools. | Technical Skills | Cross Functional Skills | -2.459 |
| Science | Using scientific rules and methods to solve problems. | Content | Basic Skills | 0.567 |
| Service Orientation | Actively looking for ways to help people. | Social Skills | Cross Functional Skills | -0.651 |
| Social Perceptiveness | Being aware of others' reactions and understanding why they react as they do. | Social Skills | Cross Functional Skills | 0 |
| Speaking | Talking to others to convey information effectively. | Content | Basic Skills | 5.976 |
| System Analysis | Determining how a system should work and how changes in conditions, operations, and the environment will affect outcomes. | Systems Skills | Cross Functional Skills | -1.404 |
| System Evaluation | Identifying measures or indicators of system performance and the actions needed to improve or correct performance, relative to the goals of the system. | Systems Skills | Cross Functional Skills | 5.355 |
| Technology Design | Generating or adapting equipment and technology to serve user needs. | Technical Skills | Cross Functional Skills | -5.659 |
| Time Management | Managing one's own time and the time of others. | Resource Management Skills | Cross Functional Skills | 3.92 |
| Troubleshooting | Determining causes of operating errors and deciding what to do about it. | Technical Skills | Cross Functional Skills | 1.146 |
| Writing | Communicating effectively in writing as appropriate for the needs of the audience. | Content | Basic Skills | 0 |
| R squared = 0.87 |  |  | N= | 75418 |

H3 Spain People, Brains, Brawn Analysis

|  | Spain – EU LFS |
| --- | --- |
| People | -0.034*** |
|  | (0.001) |
| Brains | -0.118*** |
|  | (0.001) |
| Brawn | 0.050*** |
|  | (0.001) |
| People * Brains | -0.003*** |
|  | (0.001) |
| People*Brawn | 0.037*** |
|  | (0.001) |
| Brains*Brawn | -0.039*** |
|  | (0.001) |
| N | 75403 |
| R Squared | 22% |

S11 Appendix. Individual country Finland

Table I1 Finland Ability Lasso Analysis

| ONET Item | Description |  |  | Coefficient |
| --- | --- | --- | --- | --- |
| Arm-Hand Steadiness | The ability to keep your hand and arm steady while moving your arm or while holding your arm and hand in one position. | Fine manipulative abilities | -3.927 | -3.927 |
| Auditory Attention | The ability to focus on a single source of sound in the presence of other distracting sounds. | Auditory and speech abilities | 0 | 0 |
| Category Flexibility | The ability to generate or use different sets of rules for combining or grouping things in different ways. | Idea Generation and Reasoning Abilities | -2.306 | -2.306 |
| Control Precision | The ability to quickly and repeatedly adjust the controls of a machine or a vehicle to exact positions. | Control Movement Abilities | 3.07 | 3.07 |
| Deductive Reasoning | The ability to apply general rules to specific problems to produce answers that make sense. | Idea Generation and Reasoning Abilities | -6.724 | -6.724 |
| Depth Perception | The ability to judge which of several objects is closer or farther away from you, or to judge the distance between you and an object. | Visual Abilities | 0 | 0 |
| Dynamic Flexibility | The ability to quickly and repeatedly bend, stretch, twist, or reach out with your body, arms, and/or legs. | Flexibility, Balance and Coordination | 3.385 | 3.385 |
| Dynamic Strength | The ability to exert muscle force repeatedly or continuously over time. This involves muscular endurance and resistance to muscle fatigue. | Physical Strength | -2.021 | -2.021 |
| Explosive Strength | The ability to use short bursts of muscle force to propel oneself (as in jumping or sprinting), or to throw an object. | Physical Strength | 0.578 | 0.578 |
| Extent Flexibility | The ability to bend, stretch, twist, or reach with your body, arms, and/or legs. | Flexibility, balance and coordination | -1.365 | -1.365 |
| Far Vision | The ability to see details at a distance. | Visual Abilities | -3.07 | -3.07 |
| Finger Dexterity | The ability to make precisely coordinated movements of the fingers of one or both hands to grasp, manipulate, or assemble very small objects. | Fine manipulative abilities | 2.681 | 2.681 |
| Flexibility of Closure | The ability to identify or detect a known pattern (a figure, object, word, or sound) that is hidden in other distracting material. | Perecptual Abilities | 0.288 | 0.288 |
| Fluency of ideas | The ability to come up with a number of ideas about a topic (the number of ideas is important, not their quality, correctness, or creativity). | Ideas generation and reasoning abilities | -3.484 | -3.484 |
| Glare sensitivity | The ability to see objects in the presence of glare or bright lighting. | Visual abilities | -2.553 | -2.553 |
| Gross Body Co-ordination | The ability to coordinate the movement of your arms, legs, and torso together when the whole body is in motion. | Flexibility, Balance and Coordination | 0 | 0 |
| Gross Body Equilibrium | The ability to keep or regain your body balance or stay upright when in an unstable position. | Flexibility, Balance and Coordination | 0 | 0 |
| Hearing Sensitivity | The ability to detect or tell the differences between sounds that vary in pitch and loudness. | Auditory and Speech abilities | 0 | 0 |
| Inductive Reasoning | The ability to combine pieces of information to form general rules or conclusions (includes finding a relationship among seemingly unrelated events). | Idea Generation and Reasoning Abilities | 0 | 0 |
| Information Ordering | The ability to arrange things or actions in a certain order or pattern according to a specific rule or set of rules (e.g., patterns of numbers, letters, words, pictures, mathematical operations). | Idea Generation and Reasoning Abilities | 0 | 0 |
| Manual Dexterity | The ability to quickly move your hand, your hand together with your arm, or your two hands to grasp, manipulate, or assemble objects. | Fine Manipulative Abilities | -0.829 | -0.829 |
| Math Reasoning | The ability to choose the right mathematical methods or formulas to solve a problem. | Quantitative Abilities | 0.621 | 0.621 |
| Memorization | Abilities related to the recall of available information | Quantitative Abilities | 0 | 0 |
| Multi Limb Co-ordination | The ability to coordinate two or more limbs (for example, two arms, two legs, or one leg and one arm) while sitting, standing, or lying down. It does not involve performing the activities while the whole body is in motion. | Control movement abilities | 1.073 | 1.073 |
| Near Vision | The ability to see details at close range (within a few feet of the observer) | Near Vision | 0 | 0 |
| Night Vision | The ability to see under low light conditions | Near Vision | 0 | 0 |
| Number Facility | The ability to add, subtract, multiply, or divide quickly and correctly. | Quantitative Abilities | -0.155 | -0.155 |
| Oral Comprehension | The ability to listen to and understand information and ideas presented through spoken words and sentences. | Verbal Abilities | 0 | 0 |
| Oral Expression | The ability to communicate information and ideas in speaking so others will understand. | Verbal Abilities | 4.527 | 4.527 |
| Originality |  |  | 0 | 0 |
| Perceptual Speed | The ability to quickly and accurately compare similarities and differences among sets of letters, numbers, objects, pictures, or patterns. The things to be compared may be presented at the same time or one after the other. This ability also includes comparing a presented object with a remembered object. | Perceptual Abilities | -5.531 | -5.531 |
| Perceptual Vision | The ability to see objects or movement of objects to one's side when the eyes are looking ahead. | Visual Abilities | 3.391 | 3.391 |
| Problem Sensitivity | The ability to tell when something is wrong or is likely to go wrong. It does not involve solving the problem, only recognizing there is a problem. | Idea generation and Reasoning Abilities | 2.349 | 2.349 |
| Rate Control | The ability to time your movements or the movement of a piece of equipment in anticipation of changes in the speed and/or direction of a moving object or scene. | Control Movement Abilities | 2.422 | 2.422 |
| Reaction Time | The ability to quickly respond (with the hand, finger, or foot) to a signal (sound, light, picture) when it appears. | Reaction Time and Speed Abilities | 2.546 | 2.546 |
| Response Orientation | The ability to choose quickly between two or more movements in response to two or more different signals (lights, sounds, pictures). It includes the speed with which the correct response is started with the hand, foot, or other body part. | Control Movement Abilities | -4.612 | -4.612 |
| Selective Attention | The ability to concentrate on a task over a period of time without being distracted. | Attentiveness | 0 | 0 |
| Sound Localisation | The ability to tell the direction from which a sound originated. | Auditory and Speech Abilities | -3.091 | -3.091 |
| Spatial Orientation | The ability to know your location in relation to the environment or to know where other objects are in relation to you. | Spatial Abilities | 0.643 | 0.643 |
| Speech Clarity | The ability to speak clearly so others can understand you. | Auditory and Speech Abilities | 0.784 | 0.784 |
| Speech Recognition | The ability to identify and understand the speech of another person. | Auditory and Speech Abilities | -1.664 | -1.664 |
| Speed of Closure | The ability to quickly make sense of, combine, and organize information into meaningful patterns. | Perceptual Abilities | 5.277 | 5.277 |
| Speed of Limb Movement | The ability to quickly move the arms and legs. | Reaction Time and Speed Abilities | 0 | 0 |
| Stamina | The ability to exert yourself physically over long periods of time without getting winded or out of breath. | Endurance | 0.423 | 0.423 |
| Static Strength | The ability to exert maximum muscle force to lift, push, pull, or carry objects. | Physical Strength Abilities | 0.955 | 0.955 |
| Time Sharing | The ability to shift back and forth between two or more activities or sources of information (such as speech, sounds, touch, or other sources). | Attentiveness | 2.47 | 2.47 |
| Trunk Strength | The ability to use your abdominal and lower back muscles to support part of the body repeatedly or continuously over time without 'giving out' or fatiguing. | Physical Strength Abilities | 0.127 | 0.127 |
| Visual Color Discrimination | The ability to match or detect differences between colors, including shades of color and brightness. | Visual Abilities | 4.892 | 4.892 |
| Visualisation | The ability to imagine how something will look after it is moved around or when its parts are moved or rearranged. | Spatial Abilities | -1.066 | -1.066 |
| Wrist-Finger Speed | The ability to make fast, simple, repeated movements of the fingers, hands, and wrists. | Reaction Time and Speed Abilities | -1.641 | -1.641 |
| Written Comprehension | The ability to read and understand information and ideas presented in writing. | Verbal Abilities | 0.716 | 0.716 |
| Written Expression | The ability to communicate information and ideas in writing so others will understand. | Verbal Abilities | -0.624 | -0.624 |
| R Squared = 0.98 | |  | N= 22360 | |

Table I2 Finland Skills Lasso Analysis

| ONET Item | Description |  |  | Coefficient |
| --- | --- | --- | --- | --- |
| Active Learning | Understanding the implications of new information for both current and future problem-solving and decision-making. | Process | Basic Skills | 0.794 |
| Active Listening | Giving full attention to what other people are saying, taking time to understand the points being made, asking questions as appropriate, and not interrupting at inappropriate times. | Content | Basic Skills | -2.916 |
| Complex Problem Solving | Identifying complex problems and reviewing related information to develop and evaluate options and implement solutions. | Complex Problem Solving | Cross Functional Skills | -3.736 |
| Coordination | Adjusting actions in relation to others' actions. | Social Skills | Cross Functional Skills | -7.226 |
| Critical Thinking | Using logic and reasoning to identify the strengths and weaknesses of alternative solutions, conclusions or approaches to problems. | Process | Basic Skills | -2.846 |
| Equipment Maintenance | Performing routine maintenance on equipment and determining when and what kind of maintenance is needed. | Technical Skills | Cross Functional Skills | -3.306 |
| Equipment Selection | Determining the kind of tools and equipment needed to do a job. | Technical Skills | Cross Functional Skills | 2.111 |
| Installation | Installing equipment, machines, wiring, or programs to meet specifications. | Technical Skills | Cross Functional Skills | 0.78 |
| Instructing | Teaching others how to do something. | Social Skills | Cross Functional Skills | -3.504 |
| Judgement and Decision Making | Considering the relative costs and benefits of potential actions to choose the most appropriate one. | Systems Skills | Cross Functional Skills | 5.053 |
| Learning Strategies | Selecting and using training/instructional methods and procedures appropriate for the situation when learning or teaching new things. | Systems Skills | Cross Functional Skills | 2.083 |
| Management of Financial Resources | Determining how money will be spent to get the work done, and accounting for these expenditures. | Resource Management Skills | Cross functional Skills | 0 |
| Management of Material Resources | Obtaining and seeing to the appropriate use of equipment, facilities, and materials needed to do certain work. | Resource Management Skills | Cross functional Skills | -0.678 |
| Management of Personell Resources | Motivating, developing, and directing people as they work, identifying the best people for the job. | Resource Management Skills | Cross functional Skills | 1.651 |
| Mathematics | Using mathematics to solve problems. | Content | Basic Skills | 0 |
| Monitoring | Monitoring/Assessing performance of yourself, other individuals, or organizations to make improvements or take corrective action. | Process | Basic Skills | -8.509 |
| Negotiation | Bringing others together and trying to reconcile differences. | Social Skills | Cross functional Skills | 1.244 |
| Operation Monitoring | Watching gauges, dials, or other indicators to make sure a machine is working properly. | Technical Skills | Cross Functional Skills | 5.044 |
| Operation and Control | Controlling operations of equipment or systems. | Technical Skills | Cross Functional Skills | -0.765 |
| Operations Analysis | Analyzing needs and product requirements to create a design. | Technical Skills | Cross Functional Skills | 0 |
| Persuasion | Persuading others to change their minds or behavior. | Social Skills | Cross functional Skills | 0 |
| Programming | Writing computer programs for various purposes. | Technical Skills | Cross functional Skills | 0 |
| Quality Control Analysis | Conducting tests and inspections of products, services, or processes to evaluate quality or performance. | Technical Skills | Cross functional Skills | -1.557 |
| Reading Comprehension | Understanding written sentences and paragraphs in work related documents. | Content | Basic Skills | 0.897 |
| Repairing | Repairing machines or systems using the needed tools. | Technical Skills | Cross Functional Skills | -0.34 |
| Science | Using scientific rules and methods to solve problems. | Content | Basic Skills | 0 |
| Service Orientation | Actively looking for ways to help people. | Social Skills | Cross Functional Skills | 0 |
| Social Perceptiveness | Being aware of others' reactions and understanding why they react as they do. | Social Skills | Cross Functional Skills | 0 |
| Speaking | Talking to others to convey information effectively. | Content | Basic Skills | 4.638 |
| System Analysis | Determining how a system should work and how changes in conditions, operations, and the environment will affect outcomes. | Systems Skills | Cross Functional Skills | 0 |
| System Evaluation | Identifying measures or indicators of system performance and the actions needed to improve or correct performance, relative to the goals of the system. | Systems Skills | Cross Functional Skills | 3.24 |
| Technology Design | Generating or adapting equipment and technology to serve user needs. | Technical Skills | Cross Functional Skills | -4.807 |
| Time Management | Managing one's own time and the time of others. | Resource Management Skills | Cross Functional Skills | 4.213 |
| Troubleshooting | Determining causes of operating errors and deciding what to do about it. | Technical Skills | Cross Functional Skills | 0.716 |
| Writing | Communicating effectively in writing as appropriate for the needs of the audience. | Content | Basic Skills | 0 |
| R squared = 0.80 |  |  | N= | 22360 |

Table I3 Finland People, Brains, Brawn Analysis

|  | Finland – EU LFS |
| --- | --- |
| People | 0.006*** |
|  | (0.001) |
| Brains | -0.058*** |
|  | (0.001) |
| Brawn | 0.022*** |
|  | (0.001) |
| People * Brains | -0.001** |
|  | (0.001) |
| People*Brawn | -0.004*** |
|  | (0.000) |
| Brains*Brawn | -0.011*** |
|  | (0.001) |
| N | 22315 |
| R Squared | 16% |

***, **, * denotes significance at the 1%, 5% and 10% levels respectively

S12 Appendix. Individual country France

Table J1France Ability Lasso Analysis

| ONET Item | Description |  |  | Coefficient |
| --- | --- | --- | --- | --- |
| Arm-Hand Steadiness | The ability to keep your hand and arm steady while moving your arm or while holding your arm and hand in one position. | -3.075 | Psychomotor abilities | -3.075 |
| Auditory Attention | The ability to focus on a single source of sound in the presence of other distracting sounds. | 0 | Sensory Abilities | 0 |
| Category Flexibility | The ability to generate or use different sets of rules for combining or grouping things in different ways. | -3.187 | Cognitive Abilities | -3.187 |
| Control Precision | The ability to quickly and repeatedly adjust the controls of a machine or a vehicle to exact positions. | 1.775 | Psychomotor Abilities | 1.775 |
| Deductive Reasoning | The ability to apply general rules to specific problems to produce answers that make sense. | -4.528 | Cognitive Abilities | -4.528 |
| Depth Perception | The ability to judge which of several objects is closer or farther away from you, or to judge the distance between you and an object. | 0.61 | Sensory Abilities | 0.61 |
| Dynamic Flexibility | The ability to quickly and repeatedly bend, stretch, twist, or reach out with your body, arms, and/or legs. | 3.247 | Physical Abilities | 3.247 |
| Dynamic Strength | The ability to exert muscle force repeatedly or continuously over time. This involves muscular endurance and resistance to muscle fatigue. | 0 | Physical Abilities | 0 |
| Explosive Strength | The ability to use short bursts of muscle force to propel oneself (as in jumping or sprinting), or to throw an object. | 0.238 | Physical Abilities | 0.238 |
| Extent Flexibility | The ability to bend, stretch, twist, or reach with your body, arms, and/or legs. | -1.55 | Physical Abilities | -1.55 |
| Far Vision | The ability to see details at a distance. | 0 | Sensory Abilities | 0 |
| Finger Dexterity | The ability to make precisely coordinated movements of the fingers of one or both hands to grasp, manipulate, or assemble very small objects. | 1.799 | Psychomotor abilities | 1.799 |
| Flexibility of Closure | The ability to identify or detect a known pattern (a figure, object, word, or sound) that is hidden in other distracting material. | 0 | Cognitive abilities | 0 |
| Fluency of ideas | The ability to come up with a number of ideas about a topic (the number of ideas is important, not their quality, correctness, or creativity). | -1.445 | Cognitive abilities | -1.445 |
| Glare sensitivity | The ability to see objects in the presence of glare or bright lighting. | -4.373 | Sensory abilities | -4.373 |
| Gross Body Co-ordination | The ability to coordinate the movement of your arms, legs, and torso together when the whole body is in motion. | 0 | Physical Abilities | 0 |
| Gross Body Equilibrium | The ability to keep or regain your body balance or stay upright when in an unstable position. | 0 | Physical Abilities | 0 |
| Hearing Sensitivity | The ability to detect or tell the differences between sounds that vary in pitch and loudness. | -0.321 | Sensory Abilities | -0.321 |
| Inductive Reasoning | The ability to combine pieces of information to form general rules or conclusions (includes finding a relationship among seemingly unrelated events). | 0.57 | Cognitive Abilities | 0.57 |
| Information Ordering | The ability to arrange things or actions in a certain order or pattern according to a specific rule or set of rules (e.g., patterns of numbers, letters, words, pictures, mathematical operations). | 0 | Cognitive Abilities | 0 |
| Manual Dexterity | The ability to quickly move your hand, your hand together with your arm, or your two hands to grasp, manipulate, or assemble objects. | -0.164 | Psychomotor Abilities | -0.164 |
| Math Reasoning | The ability to choose the right mathematical methods or formulas to solve a problem. | 1.56 | Cognitive Abilities | 1.56 |
| Memorization | Abilities related to the recall of available information | 0 | Cognitive Abilities | 0 |
| Multi Limb Co-ordination | The ability to coordinate two or more limbs (for example, two arms, two legs, or one leg and one arm) while sitting, standing, or lying down. It does not involve performing the activities while the whole body is in motion. | 1.969 | Psychomotor Abilities | 1.969 |
| Near Vision | The ability to see details at close range (within a few feet of the observer) | 0 | Visual Abilities | 0 |
| Night Vision | The ability to see under low light conditions | 3.904 | Visual Abilities | 3.904 |
| Number Facility | The ability to add, subtract, multiply, or divide quickly and correctly. | -1.656 | Cognitive Abilities | -1.656 |
| Oral Comprehension | The ability to listen to and understand information and ideas presented through spoken words and sentences. | 2.097 | Cognitive Abilities | 2.097 |
| Oral Expression | The ability to communicate information and ideas in speaking so others will understand. | 1.942 | Cognitive Abilities | 1.942 |
| Originality |  | 0 |  | 0 |
| Perceptual Speed | The ability to quickly and accurately compare similarities and differences among sets of letters, numbers, objects, pictures, or patterns. The things to be compared may be presented at the same time or one after the other. This ability also includes comparing a presented object with a remembered object. | -5.757 | Cognitive Abilities | -5.757 |
| Perceptual Vision | The ability to see objects or movement of objects to one's side when the eyes are looking ahead. | 0 | Sensory Abilities | 0 |
| Problem Sensitivity | The ability to tell when something is wrong or is likely to go wrong. It does not involve solving the problem, only recognizing there is a problem. | 1.255 | Cognitive Abilities | 1.255 |
| Rate Control | The ability to time your movements or the movement of a piece of equipment in anticipation of changes in the speed and/or direction of a moving object or scene. | 3.481 | Psychomotor Abilities | 3.481 |
| Reaction Time | The ability to quickly respond (with the hand, finger, or foot) to a signal (sound, light, picture) when it appears. | 3.284 | Psychomotor Abilities | 3.284 |
| Response Orientation | The ability to choose quickly between two or more movements in response to two or more different signals (lights, sounds, pictures). It includes the speed with which the correct response is started with the hand, foot, or other body part. | -5.164 | Psychomotor Abilities | -5.164 |
| Selective Attention | The ability to concentrate on a task over a period of time without being distracted. | 0 | Cognitive Abilities | 0 |
| Sound Localisation | The ability to tell the direction from which a sound originated. | -1.245 | Sensory Abilities | -1.245 |
| Spatial Orientation | The ability to know your location in relation to the environment or to know where other objects are in relation to you. | 0.045 | Cognitive Abilities | 0.045 |
| Speech Clarity | The ability to speak clearly so others can understand you. | 0 | Sensory Abilities | 0 |
| Speech Recognition | The ability to identify and understand the speech of another person. | -3.058 | Sensory Abilities | -3.058 |
| Speed of Closure | The ability to quickly make sense of, combine, and organize information into meaningful patterns. | 5.115 | Cognitive Abilities | 5.115 |
| Speed of Limb Movement | The ability to quickly move the arms and legs. | -1.453 | Psychomotor Abilities | -1.453 |
| Stamina | The ability to exert yourself physically over long periods of time without getting winded or out of breath. | 0.441 | Physical Abilities | 0.441 |
| Static Strength | The ability to exert maximum muscle force to lift, push, pull, or carry objects. | 0.588 | Physical Abilities | 0.588 |
| Time Sharing | The ability to shift back and forth between two or more activities or sources of information (such as speech, sounds, touch, or other sources). | 3.113 | Cognitive Abilities | 3.113 |
| Trunk Strength | The ability to use your abdominal and lower back muscles to support part of the body repeatedly or continuously over time without 'giving out' or fatiguing. | -0.406 | Physical Abilities | -0.406 |
| Visual Color Discrimination | The ability to match or detect differences between colors, including shades of color and brightness. | 3.947 | Sensory Abilities | 3.947 |
| Visualisation | The ability to imagine how something will look after it is moved around or when its parts are moved or rearranged. | -1.684 | Cognitive Abilities | -1.684 |
| Wrist-Finger Speed | The ability to make fast, simple, repeated movements of the fingers, hands, and wrists. | -2.102 | Psychomotor Abilities | -2.102 |
| Written Comprehension | The ability to read and understand information and ideas presented in writing. | 0 | Cognitive Abilities | 0 |
| Written Expression | The ability to communicate information and ideas in writing so others will understand. | 0 | Cognitive Abilities | 0 |
| R Squared = 0.98 | |  | 367079 | |

Table J2 France Skills Lasso Analysis

| ONET Item | Description |  |  | Coefficient |
| --- | --- | --- | --- | --- |
| Active Learning | Understanding the implications of new information for both current and future problem-solving and decision-making. | Process | Basic Skills | 2.585 |
| Active Listening | Giving full attention to what other people are saying, taking time to understand the points being made, asking questions as appropriate, and not interrupting at inappropriate times. | Content | Basic Skills | 1.72 |
| Complex Problem Solving | Identifying complex problems and reviewing related information to develop and evaluate options and implement solutions. | Complex Problem Solving | Cross Functional Skills | -1.043 |
| Coordination | Adjusting actions in relation to others' actions. | Social Skills | Cross Functional Skills | -5.876 |
| Critical Thinking | Using logic and reasoning to identify the strengths and weaknesses of alternative solutions, conclusions or approaches to problems. | Process | Basic Skills | -3.085 |
| Equipment Maintenance | Performing routine maintenance on equipment and determining when and what kind of maintenance is needed. | Technical Skills | Cross Functional Skills | 0 |
| Equipment Selection | Determining the kind of tools and equipment needed to do a job. | Technical Skills | Cross Functional Skills | 0 |
| Installation | Installing equipment, machines, wiring, or programs to meet specifications. | Technical Skills | Cross Functional Skills | 0 |
| Instructing | Teaching others how to do something. | Social Skills | Cross Functional Skills | -3.466 |
| Judgement and Decision Making | Considering the relative costs and benefits of potential actions to choose the most appropriate one. | Systems Skills | Cross Functional Skills | 2.687 |
| Learning Strategies | Selecting and using training/instructional methods and procedures appropriate for the situation when learning or teaching new things. | Systems Skills | Cross Functional Skills | 0 |
| Management of Financial Resources | Determining how money will be spent to get the work done, and accounting for these expenditures. | Resource Management Skills | Cross functional Skills | 0 |
| Management of Material Resources | Obtaining and seeing to the appropriate use of equipment, facilities, and materials needed to do certain work. | Resource Management Skills | Cross functional Skills | -0.712 |
| Management of Personell Resources | Motivating, developing, and directing people as they work, identifying the best people for the job. | Resource Management Skills | Cross functional Skills | 2.68 |
| Mathematics | Using mathematics to solve problems. | Content | Basic Skills | 0 |
| Monitoring | Monitoring/Assessing performance of yourself, other individuals, or organizations to make improvements or take corrective action. | Process | Basic Skills | -3.585 |
| Negotiation | Bringing others together and trying to reconcile differences. | Social Skills | Cross functional Skills | 1.64 |
| Operation Monitoring | Watching gauges, dials, or other indicators to make sure a machine is working properly. | Technical Skills | Cross Functional Skills | 2.251 |
| Operation and Control | Controlling operations of equipment or systems. | Technical Skills | Cross Functional Skills | -0.402 |
| Operations Analysis | Analyzing needs and product requirements to create a design. | Technical Skills | Cross Functional Skills | -1.353 |
| Persuasion | Persuading others to change their minds or behavior. | Social Skills | Cross functional Skills | -0.682 |
| Programming | Writing computer programs for various purposes. | Technical Skills | Cross functional Skills | -0.608 |
| Quality Control Analysis | Conducting tests and inspections of products, services, or processes to evaluate quality or performance. | Technical Skills | Cross functional Skills | 0 |
| Reading Comprehension | Understanding written sentences and paragraphs in work related documents. | Content | Basic Skills | -3.358 |
| Repairing | Repairing machines or systems using the needed tools. | Technical Skills | Cross Functional Skills | 0 |
| Science | Using scientific rules and methods to solve problems. | Content | Basic Skills | 1.539 |
| Service Orientation | Actively looking for ways to help people. | Social Skills | Cross Functional Skills | 1.054 |
| Social Perceptiveness | Being aware of others' reactions and understanding why they react as they do. | Social Skills | Cross Functional Skills | -2.404 |
| Speaking | Talking to others to convey information effectively. | Content | Basic Skills | 0 |
| System Analysis | Determining how a system should work and how changes in conditions, operations, and the environment will affect outcomes. | Systems Skills | Cross Functional Skills | -1.958 |
| System Evaluation | Identifying measures or indicators of system performance and the actions needed to improve or correct performance, relative to the goals of the system. | Systems Skills | Cross Functional Skills | 3.461 |
| Technology Design | Generating or adapting equipment and technology to serve user needs. | Technical Skills | Cross Functional Skills | -1.35 |
| Time Management | Managing one's own time and the time of others. | Resource Management Skills | Cross Functional Skills | 4.13 |
| Troubleshooting | Determining causes of operating errors and deciding what to do about it. | Technical Skills | Cross Functional Skills | -1.584 |
| Writing | Communicating effectively in writing as appropriate for the needs of the audience. | Content | Basic Skills | 2.472 |
| R squared = 0.83 |  |  | N= | 367079 |

Table J3 France People, Brains, Brawn Analysis

| \|  \| France – EU LFS \| \| --- \| --- \| \| People \| -0.063*** \| \|  \| (0.001) \| \| Brains \| -0.105*** \| \|  \| (0.001) \| \| Brawn \| 0.059*** \| \|  \| (0.000) \| \| People * Brains \| -0.004*** \| \|  \| (0.000) \| \| People*Brawn \| -0.013*** \| \|  \| (0.000) \| \| Brains*Brawn \| -0.030*** \| \|  \| (0.000) \| \| N \| 366900 \| \| R Squared \| 21% \| |
| --- | --- | --- | --- | --- | --- | --- | --- | --- | --- | --- | --- | --- | --- | --- | --- | --- | --- | --- | --- | --- | --- | --- | --- | --- | --- | --- | --- | --- | --- | --- |

S13 Appendix. Individual country Greece

Table K1 Greece Ability Lasso Analysis

| ONET Item | Description |  |  | Coefficient |
| --- | --- | --- | --- | --- |
| Arm-Hand Steadiness | The ability to keep your hand and arm steady while moving your arm or while holding your arm and hand in one position. | Fine manipulative abilities | Psychomotor abilities | -0.294 |
| Auditory Attention | The ability to focus on a single source of sound in the presence of other distracting sounds. | Auditory and speech abilities | Sensory Abilities | -1.395 |
| Category Flexibility | The ability to generate or use different sets of rules for combining or grouping things in different ways. | Idea Generation and Reasoning Abilities | Cognitive Abilities | 0 |
| Control Precision | The ability to quickly and repeatedly adjust the controls of a machine or a vehicle to exact positions. | Control Movement Abilities | Psychomotor Abilities | 0.058 |
| Deductive Reasoning | The ability to apply general rules to specific problems to produce answers that make sense. | Idea Generation and Reasoning Abilities | Cognitive Abilities | 0 |
| Depth Perception | The ability to judge which of several objects is closer or farther away from you, or to judge the distance between you and an object. | Visual Abilities | Sensory Abilities | 2.633 |
| Dynamic Flexibility | The ability to quickly and repeatedly bend, stretch, twist, or reach out with your body, arms, and/or legs. | Flexibility, Balance and Coordination | Physical Abilities | 4.544 |
| Dynamic Strength | The ability to exert muscle force repeatedly or continuously over time. This involves muscular endurance and resistance to muscle fatigue. | Physical Strength | Physical Abilities | 0 |
| Explosive Strength | The ability to use short bursts of muscle force to propel oneself (as in jumping or sprinting), or to throw an object. | Physical Strength | Physical Abilities | 0 |
| Extent Flexibility | The ability to bend, stretch, twist, or reach with your body, arms, and/or legs. | Flexibility, balance and coordination | Physical Abilities | 0 |
| Far Vision | The ability to see details at a distance. | Visual Abilities | Sensory Abilities | 0 |
| Finger Dexterity | The ability to make precisely coordinated movements of the fingers of one or both hands to grasp, manipulate, or assemble very small objects. | Fine manipulative abilities | Psychomotor abilities | 0.563 |
| Flexibility of Closure | The ability to identify or detect a known pattern (a figure, object, word, or sound) that is hidden in other distracting material. | Perecptual Abilities | Cognitive abilities | 0 |
| Fluency of ideas | The ability to come up with a number of ideas about a topic (the number of ideas is important, not their quality, correctness, or creativity). | Ideas generation and reasoning abilities | Cognitive abilities | -1.846 |
| Glare sensitivity | The ability to see objects in the presence of glare or bright lighting. | Visual abilities | Sensory abilities | -2.368 |
| Gross Body Co-ordination | The ability to coordinate the movement of your arms, legs, and torso together when the whole body is in motion. | Flexibility, Balance and Coordination | Physical Abilities | -0.532 |
| Gross Body Equilibrium | The ability to keep or regain your body balance or stay upright when in an unstable position. | Flexibility, Balance and Coordination | Physical Abilities | -1.323 |
| Hearing Sensitivity | The ability to detect or tell the differences between sounds that vary in pitch and loudness. | Auditory and Speech abilities | Sensory Abilities | 0 |
| Inductive Reasoning | The ability to combine pieces of information to form general rules or conclusions (includes finding a relationship among seemingly unrelated events). | Idea Generation and Reasoning Abilities | Cognitive Abilities | -2.039 |
| Information Ordering | The ability to arrange things or actions in a certain order or pattern according to a specific rule or set of rules (e.g., patterns of numbers, letters, words, pictures, mathematical operations). | Idea Generation and Reasoning Abilities | Cognitive Abilities | 0 |
| Manual Dexterity | The ability to quickly move your hand, your hand together with your arm, or your two hands to grasp, manipulate, or assemble objects. | Fine Manipulative Abilities | Psychomotor Abilities | 0 |
| Math Reasoning | The ability to choose the right mathematical methods or formulas to solve a problem. | Quantitative Abilities | Cognitive Abilities | -0.704 |
| Memorization | Abilities related to the recall of available information | Quantitative Abilities | Cognitive Abilities | 1.572 |
| Multi Limb Co-ordination | The ability to coordinate two or more limbs (for example, two arms, two legs, or one leg and one arm) while sitting, standing, or lying down. It does not involve performing the activities while the whole body is in motion. | Control movement abilities | Psychomotor Abilities | 0 |
| Near Vision | The ability to see details at close range (within a few feet of the observer) | Near Vision | Visual Abilities | 0 |
| Night Vision | The ability to see under low light conditions | Near Vision | Visual Abilities | 1.132 |
| Number Facility | The ability to add, subtract, multiply, or divide quickly and correctly. | Quantitative Abilities | Cognitive Abilities | 0 |
| Oral Comprehension | The ability to listen to and understand information and ideas presented through spoken words and sentences. | Verbal Abilities | Cognitive Abilities | -2.091 |
| Oral Expression | The ability to communicate information and ideas in speaking so others will understand. | Verbal Abilities | Cognitive Abilities | 3.394 |
| Originality |  |  |  | 0 |
| Perceptual Speed | The ability to quickly and accurately compare similarities and differences among sets of letters, numbers, objects, pictures, or patterns. The things to be compared may be presented at the same time or one after the other. This ability also includes comparing a presented object with a remembered object. | Perceptual Abilities | Cognitive Abilities | -3.407 |
| Perceptual Vision | The ability to see objects or movement of objects to one's side when the eyes are looking ahead. | Visual Abilities | Sensory Abilities | 0 |
| Problem Sensitivity | The ability to tell when something is wrong or is likely to go wrong. It does not involve solving the problem, only recognizing there is a problem. | Idea generation and Reasoning Abilities | Cognitive Abilities | 0.92 |
| Rate Control | The ability to time your movements or the movement of a piece of equipment in anticipation of changes in the speed and/or direction of a moving object or scene. | Control Movement Abilities | Psychomotor Abilities | 2.834 |
| Reaction Time | The ability to quickly respond (with the hand, finger, or foot) to a signal (sound, light, picture) when it appears. | Reaction Time and Speed Abilities | Psychomotor Abilities | 1.035 |
| Response Orientation | The ability to choose quickly between two or more movements in response to two or more different signals (lights, sounds, pictures). It includes the speed with which the correct response is started with the hand, foot, or other body part. | Control Movement Abilities | Psychomotor Abilities | -1.42 |
| Selective Attention | The ability to concentrate on a task over a period of time without being distracted. | Attentiveness | Cognitive Abilities | 0 |
| Sound Localisation | The ability to tell the direction from which a sound originated. | Auditory and Speech Abilities | Sensory Abilities | 0 |
| Spatial Orientation | The ability to know your location in relation to the environment or to know where other objects are in relation to you. | Spatial Abilities | Cognitive Abilities | 1.103 |
| Speech Clarity | The ability to speak clearly so others can understand you. | Auditory and Speech Abilities | Sensory Abilities | 0 |
| Speech Recognition | The ability to identify and understand the speech of another person. | Auditory and Speech Abilities | Sensory Abilities | -0.649 |
| Speed of Closure | The ability to quickly make sense of, combine, and organize information into meaningful patterns. | Perceptual Abilities | Cognitive Abilities | 4.065 |
| Speed of Limb Movement | The ability to quickly move the arms and legs. | Reaction Time and Speed Abilities | Psychomotor Abilities | -4.254 |
| Stamina | The ability to exert yourself physically over long periods of time without getting winded or out of breath. | Endurance | Physical Abilities | 2.122 |
| Static Strength | The ability to exert maximum muscle force to lift, push, pull, or carry objects. | Physical Strength Abilities | Physical Abilities | 1.106 |
| Time Sharing | The ability to shift back and forth between two or more activities or sources of information (such as speech, sounds, touch, or other sources). | Attentiveness | Cognitive Abilities | 0 |
| Trunk Strength | The ability to use your abdominal and lower back muscles to support part of the body repeatedly or continuously over time without 'giving out' or fatiguing. | Physical Strength Abilities | Physical Abilities | -1.004 |
| Visual Color Discrimination | The ability to match or detect differences between colors, including shades of color and brightness. | Visual Abilities | Sensory Abilities | 0 |
| Visualisation | The ability to imagine how something will look after it is moved around or when its parts are moved or rearranged. | Spatial Abilities | Cognitive Abilities | -0.599 |
| Wrist-Finger Speed | The ability to make fast, simple, repeated movements of the fingers, hands, and wrists. | Reaction Time and Speed Abilities | Psychomotor Abilities | -0.197 |
| Written Comprehension | The ability to read and understand information and ideas presented in writing. | Verbal Abilities | Cognitive Abilities | 0 |
| Written Expression | The ability to communicate information and ideas in writing so others will understand. | Verbal Abilities | Cognitive Abilities | 0 |
| R Squared = 0.97 | |  | N= 152074 | |

Table K2 Greece Skills Lasso Analysis

| ONET Item | Description |  |  | Coefficient |
| --- | --- | --- | --- | --- |
| Active Learning | Understanding the implications of new information for both current and future problem-solving and decision-making. | Process | Basic Skills | 2.127 |
| Active Listening | Giving full attention to what other people are saying, taking time to understand the points being made, asking questions as appropriate, and not interrupting at inappropriate times. | Content | Basic Skills | 0 |
| Complex Problem Solving | Identifying complex problems and reviewing related information to develop and evaluate options and implement solutions. | Complex Problem Solving | Cross Functional Skills | 0 |
| Coordination | Adjusting actions in relation to others' actions. | Social Skills | Cross Functional Skills | -4.671 |
| Critical Thinking | Using logic and reasoning to identify the strengths and weaknesses of alternative solutions, conclusions or approaches to problems. | Process | Basic Skills | -1.523 |
| Equipment Maintenance | Performing routine maintenance on equipment and determining when and what kind of maintenance is needed. | Technical Skills | Cross Functional Skills | -0.898 |
| Equipment Selection | Determining the kind of tools and equipment needed to do a job. | Technical Skills | Cross Functional Skills | 0.154 |
| Installation | Installing equipment, machines, wiring, or programs to meet specifications. | Technical Skills | Cross Functional Skills | -0.228 |
| Instructing | Teaching others how to do something. | Social Skills | Cross Functional Skills | -3.095 |
| Judgement and Decision Making | Considering the relative costs and benefits of potential actions to choose the most appropriate one. | Systems Skills | Cross Functional Skills | 0 |
| Learning Strategies | Selecting and using training/instructional methods and procedures appropriate for the situation when learning or teaching new things. | Systems Skills | Cross Functional Skills | 0.452 |
| Management of Financial Resources | Determining how money will be spent to get the work done, and accounting for these expenditures. | Resource Management Skills | Cross functional Skills | 0 |
| Management of Material Resources | Obtaining and seeing to the appropriate use of equipment, facilities, and materials needed to do certain work. | Resource Management Skills | Cross functional Skills | -0.762 |
| Management of Personell Resources | Motivating, developing, and directing people as they work, identifying the best people for the job. | Resource Management Skills | Cross functional Skills | 1.493 |
| Mathematics | Using mathematics to solve problems. | Content | Basic Skills | 0 |
| Monitoring | Monitoring/Assessing performance of yourself, other individuals, or organizations to make improvements or take corrective action. | Process | Basic Skills | -4.5 |
| Negotiation | Bringing others together and trying to reconcile differences. | Social Skills | Cross functional Skills | 3.851 |
| Operation Monitoring | Watching gauges, dials, or other indicators to make sure a machine is working properly. | Technical Skills | Cross Functional Skills | 1.152 |
| Operation and Control | Controlling operations of equipment or systems. | Technical Skills | Cross Functional Skills | 0 |
| Operations Analysis | Analyzing needs and product requirements to create a design. | Technical Skills | Cross Functional Skills | -0.872 |
| Persuasion | Persuading others to change their minds or behavior. | Social Skills | Cross functional Skills | -2.221 |
| Programming | Writing computer programs for various purposes. | Technical Skills | Cross functional Skills | 0.456 |
| Quality Control Analysis | Conducting tests and inspections of products, services, or processes to evaluate quality or performance. | Technical Skills | Cross functional Skills | -1.015 |
| Reading Comprehension | Understanding written sentences and paragraphs in work related documents. | Content | Basic Skills | -1.137 |
| Repairing | Repairing machines or systems using the needed tools. | Technical Skills | Cross Functional Skills | -0.238 |
| Science | Using scientific rules and methods to solve problems. | Content | Basic Skills | 1.197 |
| Service Orientation | Actively looking for ways to help people. | Social Skills | Cross Functional Skills | 0 |
| Social Perceptiveness | Being aware of others' reactions and understanding why they react as they do. | Social Skills | Cross Functional Skills | 0 |
| Speaking | Talking to others to convey information effectively. | Content | Basic Skills | 0 |
| System Analysis | Determining how a system should work and how changes in conditions, operations, and the environment will affect outcomes. | Systems Skills | Cross Functional Skills | -3.122 |
| System Evaluation | Identifying measures or indicators of system performance and the actions needed to improve or correct performance, relative to the goals of the system. | Systems Skills | Cross Functional Skills | 4.379 |
| Technology Design | Generating or adapting equipment and technology to serve user needs. | Technical Skills | Cross Functional Skills | -2.618 |
| Time Management | Managing one's own time and the time of others. | Resource Management Skills | Cross Functional Skills | 3.711 |
| Troubleshooting | Determining causes of operating errors and deciding what to do about it. | Technical Skills | Cross Functional Skills | 1.485 |
| Writing | Communicating effectively in writing as appropriate for the needs of the audience. | Content | Basic Skills | 1.43 |
| R squared = 0.94 |  |  | N= | 152074 |

Table K3 Greece People, Brains, Brawn Analysis

|  |
| --- |
| \|  \| Greece – EU LFS \| \| --- \| --- \| \| People \| 0.021*** \| \|  \| (0.000) \| \| Brains \| -0.167*** \| \|  \| (0.001) \| \| Brawn \| 0.139*** \| \|  \| (0.001) \| \| People * Brains \| -0.002*** \| \|  \| (0.000) \| \| People*Brawn \| 0.001*** \| \|  \| (0.000) \| \| Brains*Brawn \| -0.021*** \| \|  \| (0.000) \| \| N \| 151948 \| \| R Squared \| 37% \| |

***, **, * denotes significance at the 1%, 5% and 10% levels respectively

S14 Appendix. Individual country Hungary

Table L1 Hungary Ability Lasso Analysis

| ONET Item | Description |  |  | Coefficient |
| --- | --- | --- | --- | --- |
| Arm-Hand Steadiness | The ability to keep your hand and arm steady while moving your arm or while holding your arm and hand in one position. | Fine manipulative abilities | Psychomotor abilities | -4.599 |
| Auditory Attention | The ability to focus on a single source of sound in the presence of other distracting sounds. | Auditory and speech abilities | Sensory Abilities | 0 |
| Category Flexibility | The ability to generate or use different sets of rules for combining or grouping things in different ways. | Idea Generation and Reasoning Abilities | Cognitive Abilities | -1.448 |
| Control Precision | The ability to quickly and repeatedly adjust the controls of a machine or a vehicle to exact positions. | Control Movement Abilities | Psychomotor Abilities | 2.007 |
| Deductive Reasoning | The ability to apply general rules to specific problems to produce answers that make sense. | Idea Generation and Reasoning Abilities | Cognitive Abilities | -6.082 |
| Depth Perception | The ability to judge which of several objects is closer or farther away from you, or to judge the distance between you and an object. | Visual Abilities | Sensory Abilities | 0.497 |
| Dynamic Flexibility | The ability to quickly and repeatedly bend, stretch, twist, or reach out with your body, arms, and/or legs. | Flexibility, Balance and Coordination | Physical Abilities | 3.006 |
| Dynamic Strength | The ability to exert muscle force repeatedly or continuously over time. This involves muscular endurance and resistance to muscle fatigue. | Physical Strength | Physical Abilities | -0.507 |
| Explosive Strength | The ability to use short bursts of muscle force to propel oneself (as in jumping or sprinting), or to throw an object. | Physical Strength | Physical Abilities | 1.048 |
| Extent Flexibility | The ability to bend, stretch, twist, or reach with your body, arms, and/or legs. | Flexibility, balance and coordination | Physical Abilities | -2.208 |
| Far Vision | The ability to see details at a distance. | Visual Abilities | Sensory Abilities | -2.784 |
| Finger Dexterity | The ability to make precisely coordinated movements of the fingers of one or both hands to grasp, manipulate, or assemble very small objects. | Fine manipulative abilities | Psychomotor abilities | 3.431 |
| Flexibility of Closure | The ability to identify or detect a known pattern (a figure, object, word, or sound) that is hidden in other distracting material. | Perecptual Abilities | Cognitive abilities | 0 |
| Fluency of ideas | The ability to come up with a number of ideas about a topic (the number of ideas is important, not their quality, correctness, or creativity). | Ideas generation and reasoning abilities | Cognitive abilities | -3.217 |
| Glare sensitivity | The ability to see objects in the presence of glare or bright lighting. | Visual abilities | Sensory abilities | -2.612 |
| Gross Body Co-ordination | The ability to coordinate the movement of your arms, legs, and torso together when the whole body is in motion. | Flexibility, Balance and Coordination | Physical Abilities | 0 |
| Gross Body Equilibrium | The ability to keep or regain your body balance or stay upright when in an unstable position. | Flexibility, Balance and Coordination | Physical Abilities | 0 |
| Hearing Sensitivity | The ability to detect or tell the differences between sounds that vary in pitch and loudness. | Auditory and Speech abilities | Sensory Abilities | -1.104 |
| Inductive Reasoning | The ability to combine pieces of information to form general rules or conclusions (includes finding a relationship among seemingly unrelated events). | Idea Generation and Reasoning Abilities | Cognitive Abilities | 0 |
| Information Ordering | The ability to arrange things or actions in a certain order or pattern according to a specific rule or set of rules (e.g., patterns of numbers, letters, words, pictures, mathematical operations). | Idea Generation and Reasoning Abilities | Cognitive Abilities | -2.258 |
| Manual Dexterity | The ability to quickly move your hand, your hand together with your arm, or your two hands to grasp, manipulate, or assemble objects. | Fine Manipulative Abilities | Psychomotor Abilities | 0 |
| Math Reasoning | The ability to choose the right mathematical methods or formulas to solve a problem. | Quantitative Abilities | Cognitive Abilities | 0.501 |
| Memorization | Abilities related to the recall of available information | Quantitative Abilities | Cognitive Abilities | 0 |
| Multi Limb Co-ordination | The ability to coordinate two or more limbs (for example, two arms, two legs, or one leg and one arm) while sitting, standing, or lying down. It does not involve performing the activities while the whole body is in motion. | Control movement abilities | Psychomotor Abilities | 1.04 |
| Near Vision | The ability to see details at close range (within a few feet of the observer) | Near Vision | Visual Abilities | -0.379 |
| Night Vision | The ability to see under low light conditions | Near Vision | Visual Abilities | 0 |
| Number Facility | The ability to add, subtract, multiply, or divide quickly and correctly. | Quantitative Abilities | Cognitive Abilities | -0.615 |
| Oral Comprehension | The ability to listen to and understand information and ideas presented through spoken words and sentences. | Verbal Abilities | Cognitive Abilities | 1.606 |
| Oral Expression | The ability to communicate information and ideas in speaking so others will understand. | Verbal Abilities | Cognitive Abilities | 1.957 |
| Originality |  |  |  | 0 |
| Perceptual Speed | The ability to quickly and accurately compare similarities and differences among sets of letters, numbers, objects, pictures, or patterns. The things to be compared may be presented at the same time or one after the other. This ability also includes comparing a presented object with a remembered object. | Perceptual Abilities | Cognitive Abilities | -5.517 |
| Perceptual Vision | The ability to see objects or movement of objects to one's side when the eyes are looking ahead. | Visual Abilities | Sensory Abilities | 2.063 |
| Problem Sensitivity | The ability to tell when something is wrong or is likely to go wrong. It does not involve solving the problem, only recognizing there is a problem. | Idea generation and Reasoning Abilities | Cognitive Abilities | 3.106 |
| Rate Control | The ability to time your movements or the movement of a piece of equipment in anticipation of changes in the speed and/or direction of a moving object or scene. | Control Movement Abilities | Psychomotor Abilities | 2.202 |
| Reaction Time | The ability to quickly respond (with the hand, finger, or foot) to a signal (sound, light, picture) when it appears. | Reaction Time and Speed Abilities | Psychomotor Abilities | 2.203 |
| Response Orientation | The ability to choose quickly between two or more movements in response to two or more different signals (lights, sounds, pictures). It includes the speed with which the correct response is started with the hand, foot, or other body part. | Control Movement Abilities | Psychomotor Abilities | -3.674 |
| Selective Attention | The ability to concentrate on a task over a period of time without being distracted. | Attentiveness | Cognitive Abilities | 0 |
| Sound Localisation | The ability to tell the direction from which a sound originated. | Auditory and Speech Abilities | Sensory Abilities | -1.772 |
| Spatial Orientation | The ability to know your location in relation to the environment or to know where other objects are in relation to you. | Spatial Abilities | Cognitive Abilities | 0.834 |
| Speech Clarity | The ability to speak clearly so others can understand you. | Auditory and Speech Abilities | Sensory Abilities | 1.21 |
| Speech Recognition | The ability to identify and understand the speech of another person. | Auditory and Speech Abilities | Sensory Abilities | -3.15 |
| Speed of Closure | The ability to quickly make sense of, combine, and organize information into meaningful patterns. | Perceptual Abilities | Cognitive Abilities | 5.905 |
| Speed of Limb Movement | The ability to quickly move the arms and legs. | Reaction Time and Speed Abilities | Psychomotor Abilities | 0 |
| Stamina | The ability to exert yourself physically over long periods of time without getting winded or out of breath. | Endurance | Physical Abilities | 0 |
| Static Strength | The ability to exert maximum muscle force to lift, push, pull, or carry objects. | Physical Strength Abilities | Physical Abilities | 0 |
| Time Sharing | The ability to shift back and forth between two or more activities or sources of information (such as speech, sounds, touch, or other sources). | Attentiveness | Cognitive Abilities | 3.609 |
| Trunk Strength | The ability to use your abdominal and lower back muscles to support part of the body repeatedly or continuously over time without 'giving out' or fatiguing. | Physical Strength Abilities | Physical Abilities | 0 |
| Visual Color Discrimination | The ability to match or detect differences between colors, including shades of color and brightness. | Visual Abilities | Sensory Abilities | 5.593 |
| Visualisation | The ability to imagine how something will look after it is moved around or when its parts are moved or rearranged. | Spatial Abilities | Cognitive Abilities | -1.139 |
| Wrist-Finger Speed | The ability to make fast, simple, repeated movements of the fingers, hands, and wrists. | Reaction Time and Speed Abilities | Psychomotor Abilities | -1.083 |
| Written Comprehension | The ability to read and understand information and ideas presented in writing. | Verbal Abilities | Cognitive Abilities | 0 |
| Written Expression | The ability to communicate information and ideas in writing so others will understand. | Verbal Abilities | Cognitive Abilities | 0 |
| R Squared = 0.99 | |  | N= 164373 | |

Table L2 Hungary Skills Lasso Analysis

| ONET Item | Description |  |  | Coefficient |
| --- | --- | --- | --- | --- |
| Active Learning | Understanding the implications of new information for both current and future problem-solving and decision-making. | Process | Basic Skills | 0 |
| Active Listening | Giving full attention to what other people are saying, taking time to understand the points being made, asking questions as appropriate, and not interrupting at inappropriate times. | Content | Basic Skills | -2.731 |
| Complex Problem Solving | Identifying complex problems and reviewing related information to develop and evaluate options and implement solutions. | Complex Problem Solving | Cross Functional Skills | -0.864 |
| Coordination | Adjusting actions in relation to others' actions. | Social Skills | Cross Functional Skills | -5.269 |
| Critical Thinking | Using logic and reasoning to identify the strengths and weaknesses of alternative solutions, conclusions or approaches to problems. | Process | Basic Skills | -1.674 |
| Equipment Maintenance | Performing routine maintenance on equipment and determining when and what kind of maintenance is needed. | Technical Skills | Cross Functional Skills | 0 |
| Equipment Selection | Determining the kind of tools and equipment needed to do a job. | Technical Skills | Cross Functional Skills | 2.235 |
| Installation | Installing equipment, machines, wiring, or programs to meet specifications. | Technical Skills | Cross Functional Skills | 0.393 |
| Instructing | Teaching others how to do something. | Social Skills | Cross Functional Skills | -3.135 |
| Judgement and Decision Making | Considering the relative costs and benefits of potential actions to choose the most appropriate one. | Systems Skills | Cross Functional Skills | 2.713 |
| Learning Strategies | Selecting and using training/instructional methods and procedures appropriate for the situation when learning or teaching new things. | Systems Skills | Cross Functional Skills | 1.179 |
| Management of Financial Resources | Determining how money will be spent to get the work done, and accounting for these expenditures. | Resource Management Skills | Cross functional Skills | 0 |
| Management of Material Resources | Obtaining and seeing to the appropriate use of equipment, facilities, and materials needed to do certain work. | Resource Management Skills | Cross functional Skills | -0.532 |
| Management of Personell Resources | Motivating, developing, and directing people as they work, identifying the best people for the job. | Resource Management Skills | Cross functional Skills | 0 |
| Mathematics | Using mathematics to solve problems. | Content | Basic Skills | 0 |
| Monitoring | Monitoring/Assessing performance of yourself, other individuals, or organizations to make improvements or take corrective action. | Process | Basic Skills | -7.035 |
| Negotiation | Bringing others together and trying to reconcile differences. | Social Skills | Cross functional Skills | 2.909 |
| Operation Monitoring | Watching gauges, dials, or other indicators to make sure a machine is working properly. | Technical Skills | Cross Functional Skills | 3.996 |
| Operation and Control | Controlling operations of equipment or systems. | Technical Skills | Cross Functional Skills | -1.365 |
| Operations Analysis | Analyzing needs and product requirements to create a design. | Technical Skills | Cross Functional Skills | -1.566 |
| Persuasion | Persuading others to change their minds or behavior. | Social Skills | Cross functional Skills | -0.827 |
| Programming | Writing computer programs for various purposes. | Technical Skills | Cross functional Skills | 0 |
| Quality Control Analysis | Conducting tests and inspections of products, services, or processes to evaluate quality or performance. | Technical Skills | Cross functional Skills | -0.62 |
| Reading Comprehension | Understanding written sentences and paragraphs in work related documents. | Content | Basic Skills | 0 |
| Repairing | Repairing machines or systems using the needed tools. | Technical Skills | Cross Functional Skills | -2.27 |
| Science | Using scientific rules and methods to solve problems. | Content | Basic Skills | 0.778 |
| Service Orientation | Actively looking for ways to help people. | Social Skills | Cross Functional Skills | 0 |
| Social Perceptiveness | Being aware of others' reactions and understanding why they react as they do. | Social Skills | Cross Functional Skills | -0.896 |
| Speaking | Talking to others to convey information effectively. | Content | Basic Skills | 5.296 |
| System Analysis | Determining how a system should work and how changes in conditions, operations, and the environment will affect outcomes. | Systems Skills | Cross Functional Skills | 0 |
| System Evaluation | Identifying measures or indicators of system performance and the actions needed to improve or correct performance, relative to the goals of the system. | Systems Skills | Cross Functional Skills | 3.779 |
| Technology Design | Generating or adapting equipment and technology to serve user needs. | Technical Skills | Cross Functional Skills | -4.34 |
| Time Management | Managing one's own time and the time of others. | Resource Management Skills | Cross Functional Skills | 4.124 |
| Troubleshooting | Determining causes of operating errors and deciding what to do about it. | Technical Skills | Cross Functional Skills | 0 |
| Writing | Communicating effectively in writing as appropriate for the needs of the audience. | Content | Basic Skills | 0 |
| R squared = 0.86 |  |  | N= | 164373 |

Table L4 Hungary People, Brains, Brawn Analysis

| \|  \| Hungary – EU LFS \| \| --- \| --- \| \| People \| 0.053*** \| \|  \| (0.000) \| \| Brains \| -0.171*** \| \|  \| (0.001) \| \| Brawn \| 0.064*** \| \|  \| (0.001) \| \| People * Brains \| -0.020*** \| \|  \| (0.000) \| \| People*Brawn \| -0.001*** \| \|  \| (0.000) \| \| Brains*Brawn \| -0.084*** \| \|  \| (0.001) \| \| N \| 164214 \| \| R Squared \| 30% \| |
| --- | --- | --- | --- | --- | --- | --- | --- | --- | --- | --- | --- | --- | --- | --- | --- | --- | --- | --- | --- | --- | --- | --- | --- | --- | --- | --- | --- | --- | --- | --- |

***, **, * denotes significance at the 1%, 5% and 10% levels respectively

S15 Appendix. Individual country Croatia

Table M1 Croatia Ability Lasso Analysis

| ONET Item | Description |  |  | Coefficient |
| --- | --- | --- | --- | --- |
| Arm-Hand Steadiness | The ability to keep your hand and arm steady while moving your arm or while holding your arm and hand in one position. | Fine manipulative abilities | Psychomotor abilities | -0.898 |
| Auditory Attention | The ability to focus on a single source of sound in the presence of other distracting sounds. | Auditory and speech abilities | Sensory Abilities | 0 |
| Category Flexibility | The ability to generate or use different sets of rules for combining or grouping things in different ways. | Idea Generation and Reasoning Abilities | Cognitive Abilities | 0 |
| Control Precision | The ability to quickly and repeatedly adjust the controls of a machine or a vehicle to exact positions. | Control Movement Abilities | Psychomotor Abilities | 0.138 |
| Deductive Reasoning | The ability to apply general rules to specific problems to produce answers that make sense. | Idea Generation and Reasoning Abilities | Cognitive Abilities | -4.641 |
| Depth Perception | The ability to judge which of several objects is closer or farther away from you, or to judge the distance between you and an object. | Visual Abilities | Sensory Abilities | 2.394 |
| Dynamic Flexibility | The ability to quickly and repeatedly bend, stretch, twist, or reach out with your body, arms, and/or legs. | Flexibility, Balance and Coordination | Physical Abilities | 5.402 |
| Dynamic Strength | The ability to exert muscle force repeatedly or continuously over time. This involves muscular endurance and resistance to muscle fatigue. | Physical Strength | Physical Abilities | -0.974 |
| Explosive Strength | The ability to use short bursts of muscle force to propel oneself (as in jumping or sprinting), or to throw an object. | Physical Strength | Physical Abilities | 0.415 |
| Extent Flexibility | The ability to bend, stretch, twist, or reach with your body, arms, and/or legs. | Flexibility, balance and coordination | Physical Abilities | -0.761 |
| Far Vision | The ability to see details at a distance. | Visual Abilities | Sensory Abilities | -0.47 |
| Finger Dexterity | The ability to make precisely coordinated movements of the fingers of one or both hands to grasp, manipulate, or assemble very small objects. | Fine manipulative abilities | Psychomotor abilities | 0.728 |
| Flexibility of Closure | The ability to identify or detect a known pattern (a figure, object, word, or sound) that is hidden in other distracting material. | Perecptual Abilities | Cognitive abilities | 0 |
| Fluency of ideas | The ability to come up with a number of ideas about a topic (the number of ideas is important, not their quality, correctness, or creativity). | Ideas generation and reasoning abilities | Cognitive abilities | -2.232 |
| Glare sensitivity | The ability to see objects in the presence of glare or bright lighting. | Visual abilities | Sensory abilities | -2.893 |
| Gross Body Co-ordination | The ability to coordinate the movement of your arms, legs, and torso together when the whole body is in motion. | Flexibility, Balance and Coordination | Physical Abilities | 0 |
| Gross Body Equilibrium | The ability to keep or regain your body balance or stay upright when in an unstable position. | Flexibility, Balance and Coordination | Physical Abilities | 0 |
| Hearing Sensitivity | The ability to detect or tell the differences between sounds that vary in pitch and loudness. | Auditory and Speech abilities | Sensory Abilities | 0 |
| Inductive Reasoning | The ability to combine pieces of information to form general rules or conclusions (includes finding a relationship among seemingly unrelated events). | Idea Generation and Reasoning Abilities | Cognitive Abilities | 0 |
| Information Ordering | The ability to arrange things or actions in a certain order or pattern according to a specific rule or set of rules (e.g., patterns of numbers, letters, words, pictures, mathematical operations). | Idea Generation and Reasoning Abilities | Cognitive Abilities | 0 |
| Manual Dexterity | The ability to quickly move your hand, your hand together with your arm, or your two hands to grasp, manipulate, or assemble objects. | Fine Manipulative Abilities | Psychomotor Abilities | 0.699 |
| Math Reasoning | The ability to choose the right mathematical methods or formulas to solve a problem. | Quantitative Abilities | Cognitive Abilities | 0 |
| Memorization | Abilities related to the recall of available information | Quantitative Abilities | Cognitive Abilities | 2.035 |
| Multi Limb Co-ordination | The ability to coordinate two or more limbs (for example, two arms, two legs, or one leg and one arm) while sitting, standing, or lying down. It does not involve performing the activities while the whole body is in motion. | Control movement abilities | Psychomotor Abilities | 0 |
| Near Vision | The ability to see details at close range (within a few feet of the observer) | Near Vision | Visual Abilities | 0 |
| Night Vision | The ability to see under low light conditions | Near Vision | Visual Abilities | 1.63 |
| Number Facility | The ability to add, subtract, multiply, or divide quickly and correctly. | Quantitative Abilities | Cognitive Abilities | 0 |
| Oral Comprehension | The ability to listen to and understand information and ideas presented through spoken words and sentences. | Verbal Abilities | Cognitive Abilities | -2.136 |
| Oral Expression | The ability to communicate information and ideas in speaking so others will understand. | Verbal Abilities | Cognitive Abilities | 3.462 |
| Originality |  |  |  | 0 |
| Perceptual Speed | The ability to quickly and accurately compare similarities and differences among sets of letters, numbers, objects, pictures, or patterns. The things to be compared may be presented at the same time or one after the other. This ability also includes comparing a presented object with a remembered object. | Perceptual Abilities | Cognitive Abilities | -3.308 |
| Perceptual Vision | The ability to see objects or movement of objects to one's side when the eyes are looking ahead. | Visual Abilities | Sensory Abilities | 0 |
| Problem Sensitivity | The ability to tell when something is wrong or is likely to go wrong. It does not involve solving the problem, only recognizing there is a problem. | Idea generation and Reasoning Abilities | Cognitive Abilities | 1.917 |
| Rate Control | The ability to time your movements or the movement of a piece of equipment in anticipation of changes in the speed and/or direction of a moving object or scene. | Control Movement Abilities | Psychomotor Abilities | 2.446 |
| Reaction Time | The ability to quickly respond (with the hand, finger, or foot) to a signal (sound, light, picture) when it appears. | Reaction Time and Speed Abilities | Psychomotor Abilities | 1.034 |
| Response Orientation | The ability to choose quickly between two or more movements in response to two or more different signals (lights, sounds, pictures). It includes the speed with which the correct response is started with the hand, foot, or other body part. | Control Movement Abilities | Psychomotor Abilities | -2.19 |
| Selective Attention | The ability to concentrate on a task over a period of time without being distracted. | Attentiveness | Cognitive Abilities | 0 |
| Sound Localisation | The ability to tell the direction from which a sound originated. | Auditory and Speech Abilities | Sensory Abilities | 0 |
| Spatial Orientation | The ability to know your location in relation to the environment or to know where other objects are in relation to you. | Spatial Abilities | Cognitive Abilities | 1.356 |
| Speech Clarity | The ability to speak clearly so others can understand you. | Auditory and Speech Abilities | Sensory Abilities | 0 |
| Speech Recognition | The ability to identify and understand the speech of another person. | Auditory and Speech Abilities | Sensory Abilities | -1.366 |
| Speed of Closure | The ability to quickly make sense of, combine, and organize information into meaningful patterns. | Perceptual Abilities | Cognitive Abilities | 3.805 |
| Speed of Limb Movement | The ability to quickly move the arms and legs. | Reaction Time and Speed Abilities | Psychomotor Abilities | -3.13 |
| Stamina | The ability to exert yourself physically over long periods of time without getting winded or out of breath. | Endurance | Physical Abilities | 1.371 |
| Static Strength | The ability to exert maximum muscle force to lift, push, pull, or carry objects. | Physical Strength Abilities | Physical Abilities | 0.601 |
| Time Sharing | The ability to shift back and forth between two or more activities or sources of information (such as speech, sounds, touch, or other sources). | Attentiveness | Cognitive Abilities | 0 |
| Trunk Strength | The ability to use your abdominal and lower back muscles to support part of the body repeatedly or continuously over time without 'giving out' or fatiguing. | Physical Strength Abilities | Physical Abilities | -1.535 |
| Visual Color Discrimination | The ability to match or detect differences between colors, including shades of color and brightness. | Visual Abilities | Sensory Abilities | 0 |
| Visualisation | The ability to imagine how something will look after it is moved around or when its parts are moved or rearranged. | Spatial Abilities | Cognitive Abilities | -1.446 |
| Wrist-Finger Speed | The ability to make fast, simple, repeated movements of the fingers, hands, and wrists. | Reaction Time and Speed Abilities | Psychomotor Abilities | 0 |
| Written Comprehension | The ability to read and understand information and ideas presented in writing. | Verbal Abilities | Cognitive Abilities | 0 |
| Written Expression | The ability to communicate information and ideas in writing so others will understand. | Verbal Abilities | Cognitive Abilities | 0 |
| R Squared = 0.97 | |  | N= 174713 | |

Table M2 Croatia Skills Lasso Analysis

| ONET Item | Description |  |  | Coefficient |
| --- | --- | --- | --- | --- |
| Active Learning | Understanding the implications of new information for both current and future problem-solving and decision-making. | Process | Basic Skills | 0 |
| Active Listening | Giving full attention to what other people are saying, taking time to understand the points being made, asking questions as appropriate, and not interrupting at inappropriate times. | Content | Basic Skills | -0.255 |
| Complex Problem Solving | Identifying complex problems and reviewing related information to develop and evaluate options and implement solutions. | Complex Problem Solving | Cross Functional Skills | -1.483 |
| Coordination | Adjusting actions in relation to others' actions. | Social Skills | Cross Functional Skills | -4.452 |
| Critical Thinking | Using logic and reasoning to identify the strengths and weaknesses of alternative solutions, conclusions or approaches to problems. | Process | Basic Skills | 0 |
| Equipment Maintenance | Performing routine maintenance on equipment and determining when and what kind of maintenance is needed. | Technical Skills | Cross Functional Skills | -0.573 |
| Equipment Selection | Determining the kind of tools and equipment needed to do a job. | Technical Skills | Cross Functional Skills | 0 |
| Installation | Installing equipment, machines, wiring, or programs to meet specifications. | Technical Skills | Cross Functional Skills | 0 |
| Instructing | Teaching others how to do something. | Social Skills | Cross Functional Skills | -3.273 |
| Judgement and Decision Making | Considering the relative costs and benefits of potential actions to choose the most appropriate one. | Systems Skills | Cross Functional Skills | 1.187 |
| Learning Strategies | Selecting and using training/instructional methods and procedures appropriate for the situation when learning or teaching new things. | Systems Skills | Cross Functional Skills | 1.744 |
| Management of Financial Resources | Determining how money will be spent to get the work done, and accounting for these expenditures. | Resource Management Skills | Cross functional Skills | 0 |
| Management of Material Resources | Obtaining and seeing to the appropriate use of equipment, facilities, and materials needed to do certain work. | Resource Management Skills | Cross functional Skills | -0.522 |
| Management of Personell Resources | Motivating, developing, and directing people as they work, identifying the best people for the job. | Resource Management Skills | Cross functional Skills | 2.008 |
| Mathematics | Using mathematics to solve problems. | Content | Basic Skills | 0 |
| Monitoring | Monitoring/Assessing performance of yourself, other individuals, or organizations to make improvements or take corrective action. | Process | Basic Skills | -5.153 |
| Negotiation | Bringing others together and trying to reconcile differences. | Social Skills | Cross functional Skills | 3.384 |
| Operation Monitoring | Watching gauges, dials, or other indicators to make sure a machine is working properly. | Technical Skills | Cross Functional Skills | 2.632 |
| Operation and Control | Controlling operations of equipment or systems. | Technical Skills | Cross Functional Skills | -0.346 |
| Operations Analysis | Analyzing needs and product requirements to create a design. | Technical Skills | Cross Functional Skills | -0.457 |
| Persuasion | Persuading others to change their minds or behavior. | Social Skills | Cross functional Skills | -1.581 |
| Programming | Writing computer programs for various purposes. | Technical Skills | Cross functional Skills | 0.32 |
| Quality Control Analysis | Conducting tests and inspections of products, services, or processes to evaluate quality or performance. | Technical Skills | Cross functional Skills | -1.04 |
| Reading Comprehension | Understanding written sentences and paragraphs in work related documents. | Content | Basic Skills | 0 |
| Repairing | Repairing machines or systems using the needed tools. | Technical Skills | Cross Functional Skills | 0 |
| Science | Using scientific rules and methods to solve problems. | Content | Basic Skills | 1.088 |
| Service Orientation | Actively looking for ways to help people. | Social Skills | Cross Functional Skills | 0.639 |
| Social Perceptiveness | Being aware of others' reactions and understanding why they react as they do. | Social Skills | Cross Functional Skills | -1.748 |
| Speaking | Talking to others to convey information effectively. | Content | Basic Skills | 0 |
| System Analysis | Determining how a system should work and how changes in conditions, operations, and the environment will affect outcomes. | Systems Skills | Cross Functional Skills | -2.376 |
| System Evaluation | Identifying measures or indicators of system performance and the actions needed to improve or correct performance, relative to the goals of the system. | Systems Skills | Cross Functional Skills | 3.346 |
| Technology Design | Generating or adapting equipment and technology to serve user needs. | Technical Skills | Cross Functional Skills | -2.675 |
| Time Management | Managing one's own time and the time of others. | Resource Management Skills | Cross Functional Skills | 2.694 |
| Troubleshooting | Determining causes of operating errors and deciding what to do about it. | Technical Skills | Cross Functional Skills | 0 |
| Writing | Communicating effectively in writing as appropriate for the needs of the audience. | Content | Basic Skills | 1.579 |
| R squared = 0.86 |  |  | N= | 174713 |

Table M3 Croatia People, Brains, Brawn Analysis

| \|  \| Croatia – EU LFS \| \| --- \| --- \| \| People \| -0.022*** \| \|  \| (0.000) \| \| Brains \| 0.113*** \| \|  \| (0.001) \| \| Brawn \| -0.149*** \| \|  \| (0.001) \| \| People * Brains \| -0.022*** \| \|  \| (0.000) \| \| People*Brawn \| 0.113*** \| \|  \| (0.001) \| \| Brains*Brawn \| -0.149*** \| \|  \| (0.001) \| \| N \| 174579 \| \| R Squared \| 29% \| |
| --- | --- | --- | --- | --- | --- | --- | --- | --- | --- | --- | --- | --- | --- | --- | --- | --- | --- | --- | --- | --- | --- | --- | --- | --- | --- | --- | --- | --- | --- | --- |

***, **, * denotes significance at the 1%, 5% and 10% levels respectively

S16 Appendix. Individual country Ireland

Table N1 Ireland Ability Lasso Analysis

| ONET Item | Description |  |  | Coefficient |
| --- | --- | --- | --- | --- |
| Arm-Hand Steadiness | The ability to keep your hand and arm steady while moving your arm or while holding your arm and hand in one position. | Fine manipulative abilities | Psychomotor abilities | -4.654 |
| Auditory Attention | The ability to focus on a single source of sound in the presence of other distracting sounds. | Auditory and speech abilities | Sensory Abilities | 0 |
| Category Flexibility | The ability to generate or use different sets of rules for combining or grouping things in different ways. | Idea Generation and Reasoning Abilities | Cognitive Abilities | -1.974 |
| Control Precision | The ability to quickly and repeatedly adjust the controls of a machine or a vehicle to exact positions. | Control Movement Abilities | Psychomotor Abilities | 1.741 |
| Deductive Reasoning | The ability to apply general rules to specific problems to produce answers that make sense. | Idea Generation and Reasoning Abilities | Cognitive Abilities | -4.082 |
| Depth Perception | The ability to judge which of several objects is closer or farther away from you, or to judge the distance between you and an object. | Visual Abilities | Sensory Abilities | 0 |
| Dynamic Flexibility | The ability to quickly and repeatedly bend, stretch, twist, or reach out with your body, arms, and/or legs. | Flexibility, Balance and Coordination | Physical Abilities | 3.508 |
| Dynamic Strength | The ability to exert muscle force repeatedly or continuously over time. This involves muscular endurance and resistance to muscle fatigue. | Physical Strength | Physical Abilities | -0.79 |
| Explosive Strength | The ability to use short bursts of muscle force to propel oneself (as in jumping or sprinting), or to throw an object. | Physical Strength | Physical Abilities | 0.334 |
| Extent Flexibility | The ability to bend, stretch, twist, or reach with your body, arms, and/or legs. | Flexibility, balance and coordination | Physical Abilities | -2.022 |
| Far Vision | The ability to see details at a distance. | Visual Abilities | Sensory Abilities | -1.285 |
| Finger Dexterity | The ability to make precisely coordinated movements of the fingers of one or both hands to grasp, manipulate, or assemble very small objects. | Fine manipulative abilities | Psychomotor abilities | 1.536 |
| Flexibility of Closure | The ability to identify or detect a known pattern (a figure, object, word, or sound) that is hidden in other distracting material. | Perecptual Abilities | Cognitive abilities | -0.375 |
| Fluency of ideas | The ability to come up with a number of ideas about a topic (the number of ideas is important, not their quality, correctness, or creativity). | Ideas generation and reasoning abilities | Cognitive abilities | -3.347 |
| Glare sensitivity | The ability to see objects in the presence of glare or bright lighting. | Visual abilities | Sensory abilities | -1.496 |
| Gross Body Co-ordination | The ability to coordinate the movement of your arms, legs, and torso together when the whole body is in motion. | Flexibility, Balance and Coordination | Physical Abilities | 1.37 |
| Gross Body Equilibrium | The ability to keep or regain your body balance or stay upright when in an unstable position. | Flexibility, Balance and Coordination | Physical Abilities | -1.476 |
| Hearing Sensitivity | The ability to detect or tell the differences between sounds that vary in pitch and loudness. | Auditory and Speech abilities | Sensory Abilities | 0 |
| Inductive Reasoning | The ability to combine pieces of information to form general rules or conclusions (includes finding a relationship among seemingly unrelated events). | Idea Generation and Reasoning Abilities | Cognitive Abilities | 0 |
| Information Ordering | The ability to arrange things or actions in a certain order or pattern according to a specific rule or set of rules (e.g., patterns of numbers, letters, words, pictures, mathematical operations). | Idea Generation and Reasoning Abilities | Cognitive Abilities | 0 |
| Manual Dexterity | The ability to quickly move your hand, your hand together with your arm, or your two hands to grasp, manipulate, or assemble objects. | Fine Manipulative Abilities | Psychomotor Abilities | 0 |
| Math Reasoning | The ability to choose the right mathematical methods or formulas to solve a problem. | Quantitative Abilities | Cognitive Abilities | 0 |
| Memorization | Abilities related to the recall of available information | Quantitative Abilities | Cognitive Abilities | 1.707 |
| Multi Limb Co-ordination | The ability to coordinate two or more limbs (for example, two arms, two legs, or one leg and one arm) while sitting, standing, or lying down. It does not involve performing the activities while the whole body is in motion. | Control movement abilities | Psychomotor Abilities | 1.907 |
| Near Vision | The ability to see details at close range (within a few feet of the observer) | Near Vision | Visual Abilities | 1.381 |
| Night Vision | The ability to see under low light conditions | Near Vision | Visual Abilities | 0 |
| Number Facility | The ability to add, subtract, multiply, or divide quickly and correctly. | Quantitative Abilities | Cognitive Abilities | 0 |
| Oral Comprehension | The ability to listen to and understand information and ideas presented through spoken words and sentences. | Verbal Abilities | Cognitive Abilities | 0 |
| Oral Expression | The ability to communicate information and ideas in speaking so others will understand. | Verbal Abilities | Cognitive Abilities | 4.061 |
| Originality |  |  |  | -1.99 |
| Perceptual Speed | The ability to quickly and accurately compare similarities and differences among sets of letters, numbers, objects, pictures, or patterns. The things to be compared may be presented at the same time or one after the other. This ability also includes comparing a presented object with a remembered object. | Perceptual Abilities | Cognitive Abilities | -6.289 |
| Perceptual Vision | The ability to see objects or movement of objects to one's side when the eyes are looking ahead. | Visual Abilities | Sensory Abilities | 3.52 |
| Problem Sensitivity | The ability to tell when something is wrong or is likely to go wrong. It does not involve solving the problem, only recognizing there is a problem. | Idea generation and Reasoning Abilities | Cognitive Abilities | 3.247 |
| Rate Control | The ability to time your movements or the movement of a piece of equipment in anticipation of changes in the speed and/or direction of a moving object or scene. | Control Movement Abilities | Psychomotor Abilities | 1.426 |
| Reaction Time | The ability to quickly respond (with the hand, finger, or foot) to a signal (sound, light, picture) when it appears. | Reaction Time and Speed Abilities | Psychomotor Abilities | 2.62 |
| Response Orientation | The ability to choose quickly between two or more movements in response to two or more different signals (lights, sounds, pictures). It includes the speed with which the correct response is started with the hand, foot, or other body part. | Control Movement Abilities | Psychomotor Abilities | -4.214 |
| Selective Attention | The ability to concentrate on a task over a period of time without being distracted. | Attentiveness | Cognitive Abilities | -2.272 |
| Sound Localisation | The ability to tell the direction from which a sound originated. | Auditory and Speech Abilities | Sensory Abilities | -2.465 |
| Spatial Orientation | The ability to know your location in relation to the environment or to know where other objects are in relation to you. | Spatial Abilities | Cognitive Abilities | 0.759 |
| Speech Clarity | The ability to speak clearly so others can understand you. | Auditory and Speech Abilities | Sensory Abilities | 0 |
| Speech Recognition | The ability to identify and understand the speech of another person. | Auditory and Speech Abilities | Sensory Abilities | -1.973 |
| Speed of Closure | The ability to quickly make sense of, combine, and organize information into meaningful patterns. | Perceptual Abilities | Cognitive Abilities | 4.336 |
| Speed of Limb Movement | The ability to quickly move the arms and legs. | Reaction Time and Speed Abilities | Psychomotor Abilities | -2.001 |
| Stamina | The ability to exert yourself physically over long periods of time without getting winded or out of breath. | Endurance | Physical Abilities | 1.651 |
| Static Strength | The ability to exert maximum muscle force to lift, push, pull, or carry objects. | Physical Strength Abilities | Physical Abilities | 0.446 |
| Time Sharing | The ability to shift back and forth between two or more activities or sources of information (such as speech, sounds, touch, or other sources). | Attentiveness | Cognitive Abilities | 3.184 |
| Trunk Strength | The ability to use your abdominal and lower back muscles to support part of the body repeatedly or continuously over time without 'giving out' or fatiguing. | Physical Strength Abilities | Physical Abilities | 0 |
| Visual Color Discrimination | The ability to match or detect differences between colors, including shades of color and brightness. | Visual Abilities | Sensory Abilities | 5.156 |
| Visualisation | The ability to imagine how something will look after it is moved around or when its parts are moved or rearranged. | Spatial Abilities | Cognitive Abilities | 0 |
| Wrist-Finger Speed | The ability to make fast, simple, repeated movements of the fingers, hands, and wrists. | Reaction Time and Speed Abilities | Psychomotor Abilities | 0 |
| Written Comprehension | The ability to read and understand information and ideas presented in writing. | Verbal Abilities | Cognitive Abilities | 0 |
| Written Expression | The ability to communicate information and ideas in writing so others will understand. | Verbal Abilities | Cognitive Abilities | 0 |
| R Squared = 0.98 | |  | N= 123183 | |

Table N2 Ireland Skills Lasso Analysis

| ONET Item | Description |  |  | Coefficient |
| --- | --- | --- | --- | --- |
| Active Learning | Understanding the implications of new information for both current and future problem-solving and decision-making. | Process | Basic Skills | 4.753 |
| Active Listening | Giving full attention to what other people are saying, taking time to understand the points being made, asking questions as appropriate, and not interrupting at inappropriate times. | Content | Basic Skills | 1.739 |
| Complex Problem Solving | Identifying complex problems and reviewing related information to develop and evaluate options and implement solutions. | Complex Problem Solving | Cross Functional Skills | -4.003 |
| Coordination | Adjusting actions in relation to others' actions. | Social Skills | Cross Functional Skills | -5.012 |
| Critical Thinking | Using logic and reasoning to identify the strengths and weaknesses of alternative solutions, conclusions or approaches to problems. | Process | Basic Skills | -3.262 |
| Equipment Maintenance | Performing routine maintenance on equipment and determining when and what kind of maintenance is needed. | Technical Skills | Cross Functional Skills | -0.898 |
| Equipment Selection | Determining the kind of tools and equipment needed to do a job. | Technical Skills | Cross Functional Skills | 0 |
| Installation | Installing equipment, machines, wiring, or programs to meet specifications. | Technical Skills | Cross Functional Skills | 0 |
| Instructing | Teaching others how to do something. | Social Skills | Cross Functional Skills | -3.167 |
| Judgement and Decision Making | Considering the relative costs and benefits of potential actions to choose the most appropriate one. | Systems Skills | Cross Functional Skills | 0 |
| Learning Strategies | Selecting and using training/instructional methods and procedures appropriate for the situation when learning or teaching new things. | Systems Skills | Cross Functional Skills | 0 |
| Management of Financial Resources | Determining how money will be spent to get the work done, and accounting for these expenditures. | Resource Management Skills | Cross functional Skills | 0 |
| Management of Material Resources | Obtaining and seeing to the appropriate use of equipment, facilities, and materials needed to do certain work. | Resource Management Skills | Cross functional Skills | 0 |
| Management of Personell Resources | Motivating, developing, and directing people as they work, identifying the best people for the job. | Resource Management Skills | Cross functional Skills | 2.191 |
| Mathematics | Using mathematics to solve problems. | Content | Basic Skills | 0 |
| Monitoring | Monitoring/Assessing performance of yourself, other individuals, or organizations to make improvements or take corrective action. | Process | Basic Skills | -4.422 |
| Negotiation | Bringing others together and trying to reconcile differences. | Social Skills | Cross functional Skills | 0 |
| Operation Monitoring | Watching gauges, dials, or other indicators to make sure a machine is working properly. | Technical Skills | Cross Functional Skills | 3.067 |
| Operation and Control | Controlling operations of equipment or systems. | Technical Skills | Cross Functional Skills | -0.944 |
| Operations Analysis | Analyzing needs and product requirements to create a design. | Technical Skills | Cross Functional Skills | -0.572 |
| Persuasion | Persuading others to change their minds or behavior. | Social Skills | Cross functional Skills | 0 |
| Programming | Writing computer programs for various purposes. | Technical Skills | Cross functional Skills | 0 |
| Quality Control Analysis | Conducting tests and inspections of products, services, or processes to evaluate quality or performance. | Technical Skills | Cross functional Skills | -1.942 |
| Reading Comprehension | Understanding written sentences and paragraphs in work related documents. | Content | Basic Skills | 0 |
| Repairing | Repairing machines or systems using the needed tools. | Technical Skills | Cross Functional Skills | 0 |
| Science | Using scientific rules and methods to solve problems. | Content | Basic Skills | 1.221 |
| Service Orientation | Actively looking for ways to help people. | Social Skills | Cross Functional Skills | 0.173 |
| Social Perceptiveness | Being aware of others' reactions and understanding why they react as they do. | Social Skills | Cross Functional Skills | 0 |
| Speaking | Talking to others to convey information effectively. | Content | Basic Skills | 1.111 |
| System Analysis | Determining how a system should work and how changes in conditions, operations, and the environment will affect outcomes. | Systems Skills | Cross Functional Skills | -3.218 |
| System Evaluation | Identifying measures or indicators of system performance and the actions needed to improve or correct performance, relative to the goals of the system. | Systems Skills | Cross Functional Skills | 4.759 |
| Technology Design | Generating or adapting equipment and technology to serve user needs. | Technical Skills | Cross Functional Skills | -2.178 |
| Time Management | Managing one's own time and the time of others. | Resource Management Skills | Cross Functional Skills | 2.551 |
| Troubleshooting | Determining causes of operating errors and deciding what to do about it. | Technical Skills | Cross Functional Skills | 0.956 |
| Writing | Communicating effectively in writing as appropriate for the needs of the audience. | Content | Basic Skills | -0.405 |
| R squared = 0.85 |  |  | N= | 122641 |

Table N3 Ireland People, Brains, Brawn Analysis

| \|  \| Ireland – EU LFS \| \| --- \| --- \| \| People \| -0.019*** \| \|  \| (0.001) \| \| Brains \| -0.120*** \| \|  \| (0.001) \| \| Brawn \| -0.002* \| \|  \| (0.001) \| \| People * Brains \| 0.006*** \| \|  \| (0.000) \| \| People*Brawn \| -0.008*** \| \|  \| (0.000) \| \| Brains*Brawn \| 0.010*** \| \|  \| (0.000) \| \| N \| 122849 \| \| R Squared \| 15% \| |
| --- | --- | --- | --- | --- | --- | --- | --- | --- | --- | --- | --- | --- | --- | --- | --- | --- | --- | --- | --- | --- | --- | --- | --- | --- | --- | --- | --- | --- | --- | --- |

***, **, * denotes significance at the 1%, 5% and 10% levels respectively

S17 Appendix. Individual country Iceland

Table O1 Iceland Ability Lasso Analysis

| ONET Item | Description |  |  | Coefficient |
| --- | --- | --- | --- | --- |
| Arm-Hand Steadiness | The ability to keep your hand and arm steady while moving your arm or while holding your arm and hand in one position. | Fine manipulative abilities | Psychomotor abilities | -3.512 |
| Auditory Attention | The ability to focus on a single source of sound in the presence of other distracting sounds. | Auditory and speech abilities | Sensory Abilities | 0 |
| Category Flexibility | The ability to generate or use different sets of rules for combining or grouping things in different ways. | Idea Generation and Reasoning Abilities | Cognitive Abilities | -4.137 |
| Control Precision | The ability to quickly and repeatedly adjust the controls of a machine or a vehicle to exact positions. | Control Movement Abilities | Psychomotor Abilities | 1.491 |
| Deductive Reasoning | The ability to apply general rules to specific problems to produce answers that make sense. | Idea Generation and Reasoning Abilities | Cognitive Abilities | -4.495 |
| Depth Perception | The ability to judge which of several objects is closer or farther away from you, or to judge the distance between you and an object. | Visual Abilities | Sensory Abilities | 0 |
| Dynamic Flexibility | The ability to quickly and repeatedly bend, stretch, twist, or reach out with your body, arms, and/or legs. | Flexibility, Balance and Coordination | Physical Abilities | 3.559 |
| Dynamic Strength | The ability to exert muscle force repeatedly or continuously over time. This involves muscular endurance and resistance to muscle fatigue. | Physical Strength | Physical Abilities | -0.745 |
| Explosive Strength | The ability to use short bursts of muscle force to propel oneself (as in jumping or sprinting), or to throw an object. | Physical Strength | Physical Abilities | 1.156 |
| Extent Flexibility | The ability to bend, stretch, twist, or reach with your body, arms, and/or legs. | Flexibility, balance and coordination | Physical Abilities | -3.368 |
| Far Vision | The ability to see details at a distance. | Visual Abilities | Sensory Abilities | -2.679 |
| Finger Dexterity | The ability to make precisely coordinated movements of the fingers of one or both hands to grasp, manipulate, or assemble very small objects. | Fine manipulative abilities | Psychomotor abilities | 2.018 |
| Flexibility of Closure | The ability to identify or detect a known pattern (a figure, object, word, or sound) that is hidden in other distracting material. | Perecptual Abilities | Cognitive abilities | -0.537 |
| Fluency of ideas | The ability to come up with a number of ideas about a topic (the number of ideas is important, not their quality, correctness, or creativity). | Ideas generation and reasoning abilities | Cognitive abilities | -3.992 |
| Glare sensitivity | The ability to see objects in the presence of glare or bright lighting. | Visual abilities | Sensory abilities | -2.928 |
| Gross Body Co-ordination | The ability to coordinate the movement of your arms, legs, and torso together when the whole body is in motion. | Flexibility, Balance and Coordination | Physical Abilities | 0 |
| Gross Body Equilibrium | The ability to keep or regain your body balance or stay upright when in an unstable position. | Flexibility, Balance and Coordination | Physical Abilities | 0 |
| Hearing Sensitivity | The ability to detect or tell the differences between sounds that vary in pitch and loudness. | Auditory and Speech abilities | Sensory Abilities | -1.421 |
| Inductive Reasoning | The ability to combine pieces of information to form general rules or conclusions (includes finding a relationship among seemingly unrelated events). | Idea Generation and Reasoning Abilities | Cognitive Abilities | 1.139 |
| Information Ordering | The ability to arrange things or actions in a certain order or pattern according to a specific rule or set of rules (e.g., patterns of numbers, letters, words, pictures, mathematical operations). | Idea Generation and Reasoning Abilities | Cognitive Abilities | 0 |
| Manual Dexterity | The ability to quickly move your hand, your hand together with your arm, or your two hands to grasp, manipulate, or assemble objects. | Fine Manipulative Abilities | Psychomotor Abilities | 0.27 |
| Math Reasoning | The ability to choose the right mathematical methods or formulas to solve a problem. | Quantitative Abilities | Cognitive Abilities | 0.515 |
| Memorization | Abilities related to the recall of available information | Quantitative Abilities | Cognitive Abilities | 0.698 |
| Multi Limb Co-ordination | The ability to coordinate two or more limbs (for example, two arms, two legs, or one leg and one arm) while sitting, standing, or lying down. It does not involve performing the activities while the whole body is in motion. | Control movement abilities | Psychomotor Abilities | 1.736 |
| Near Vision | The ability to see details at close range (within a few feet of the observer) | Near Vision | Visual Abilities | -1.318 |
| Night Vision | The ability to see under low light conditions | Near Vision | Visual Abilities | 1.642 |
| Number Facility | The ability to add, subtract, multiply, or divide quickly and correctly. | Quantitative Abilities | Cognitive Abilities | -0.657 |
| Oral Comprehension | The ability to listen to and understand information and ideas presented through spoken words and sentences. | Verbal Abilities | Cognitive Abilities | 1.054 |
| Oral Expression | The ability to communicate information and ideas in speaking so others will understand. | Verbal Abilities | Cognitive Abilities | 3.677 |
| Originality |  |  |  | 0.774 |
| Perceptual Speed | The ability to quickly and accurately compare similarities and differences among sets of letters, numbers, objects, pictures, or patterns. The things to be compared may be presented at the same time or one after the other. This ability also includes comparing a presented object with a remembered object. | Perceptual Abilities | Cognitive Abilities | -3.154 |
| Perceptual Vision | The ability to see objects or movement of objects to one's side when the eyes are looking ahead. | Visual Abilities | Sensory Abilities | 0 |
| Problem Sensitivity | The ability to tell when something is wrong or is likely to go wrong. It does not involve solving the problem, only recognizing there is a problem. | Idea generation and Reasoning Abilities | Cognitive Abilities | 1.901 |
| Rate Control | The ability to time your movements or the movement of a piece of equipment in anticipation of changes in the speed and/or direction of a moving object or scene. | Control Movement Abilities | Psychomotor Abilities | 3.282 |
| Reaction Time | The ability to quickly respond (with the hand, finger, or foot) to a signal (sound, light, picture) when it appears. | Reaction Time and Speed Abilities | Psychomotor Abilities | 0.848 |
| Response Orientation | The ability to choose quickly between two or more movements in response to two or more different signals (lights, sounds, pictures). It includes the speed with which the correct response is started with the hand, foot, or other body part. | Control Movement Abilities | Psychomotor Abilities | -3.731 |
| Selective Attention | The ability to concentrate on a task over a period of time without being distracted. | Attentiveness | Cognitive Abilities | 0 |
| Sound Localisation | The ability to tell the direction from which a sound originated. | Auditory and Speech Abilities | Sensory Abilities | 0 |
| Spatial Orientation | The ability to know your location in relation to the environment or to know where other objects are in relation to you. | Spatial Abilities | Cognitive Abilities | 1.15 |
| Speech Clarity | The ability to speak clearly so others can understand you. | Auditory and Speech Abilities | Sensory Abilities | 0 |
| Speech Recognition | The ability to identify and understand the speech of another person. | Auditory and Speech Abilities | Sensory Abilities | -0.834 |
| Speed of Closure | The ability to quickly make sense of, combine, and organize information into meaningful patterns. | Perceptual Abilities | Cognitive Abilities | 5.029 |
| Speed of Limb Movement | The ability to quickly move the arms and legs. | Reaction Time and Speed Abilities | Psychomotor Abilities | 0 |
| Stamina | The ability to exert yourself physically over long periods of time without getting winded or out of breath. | Endurance | Physical Abilities | 0.269 |
| Static Strength | The ability to exert maximum muscle force to lift, push, pull, or carry objects. | Physical Strength Abilities | Physical Abilities | 1.496 |
| Time Sharing | The ability to shift back and forth between two or more activities or sources of information (such as speech, sounds, touch, or other sources). | Attentiveness | Cognitive Abilities | 0 |
| Trunk Strength | The ability to use your abdominal and lower back muscles to support part of the body repeatedly or continuously over time without 'giving out' or fatiguing. | Physical Strength Abilities | Physical Abilities | 0 |
| Visual Color Discrimination | The ability to match or detect differences between colors, including shades of color and brightness. | Visual Abilities | Sensory Abilities | 4.736 |
| Visualisation | The ability to imagine how something will look after it is moved around or when its parts are moved or rearranged. | Spatial Abilities | Cognitive Abilities | 0 |
| Wrist-Finger Speed | The ability to make fast, simple, repeated movements of the fingers, hands, and wrists. | Reaction Time and Speed Abilities | Psychomotor Abilities | -1.076 |
| Written Comprehension | The ability to read and understand information and ideas presented in writing. | Verbal Abilities | Cognitive Abilities | -0.781 |
| Written Expression | The ability to communicate information and ideas in writing so others will understand. | Verbal Abilities | Cognitive Abilities | 0 |
| R Squared = 0.98 | |  | N= 16286 | |

Table O2 Iceland Skills Lasso Analysis

| ONET Item | Description |  |  | Coefficient |
| --- | --- | --- | --- | --- |
| Active Learning | Understanding the implications of new information for both current and future problem-solving and decision-making. | Process | Basic Skills | -0.856 |
| Active Listening | Giving full attention to what other people are saying, taking time to understand the points being made, asking questions as appropriate, and not interrupting at inappropriate times. | Content | Basic Skills | 0 |
| Complex Problem Solving | Identifying complex problems and reviewing related information to develop and evaluate options and implement solutions. | Complex Problem Solving | Cross Functional Skills | -2.046 |
| Coordination | Adjusting actions in relation to others' actions. | Social Skills | Cross Functional Skills | -3.562 |
| Critical Thinking | Using logic and reasoning to identify the strengths and weaknesses of alternative solutions, conclusions or approaches to problems. | Process | Basic Skills | -2.53 |
| Equipment Maintenance | Performing routine maintenance on equipment and determining when and what kind of maintenance is needed. | Technical Skills | Cross Functional Skills | -1.105 |
| Equipment Selection | Determining the kind of tools and equipment needed to do a job. | Technical Skills | Cross Functional Skills | 1.136 |
| Installation | Installing equipment, machines, wiring, or programs to meet specifications. | Technical Skills | Cross Functional Skills | 0 |
| Instructing | Teaching others how to do something. | Social Skills | Cross Functional Skills | -2.028 |
| Judgement and Decision Making | Considering the relative costs and benefits of potential actions to choose the most appropriate one. | Systems Skills | Cross Functional Skills | 3.687 |
| Learning Strategies | Selecting and using training/instructional methods and procedures appropriate for the situation when learning or teaching new things. | Systems Skills | Cross Functional Skills | 1.37 |
| Management of Financial Resources | Determining how money will be spent to get the work done, and accounting for these expenditures. | Resource Management Skills | Cross functional Skills | 0 |
| Management of Material Resources | Obtaining and seeing to the appropriate use of equipment, facilities, and materials needed to do certain work. | Resource Management Skills | Cross functional Skills | 0 |
| Management of Personell Resources | Motivating, developing, and directing people as they work, identifying the best people for the job. | Resource Management Skills | Cross functional Skills | 0 |
| Mathematics | Using mathematics to solve problems. | Content | Basic Skills | -0.713 |
| Monitoring | Monitoring/Assessing performance of yourself, other individuals, or organizations to make improvements or take corrective action. | Process | Basic Skills | -7.535 |
| Negotiation | Bringing others together and trying to reconcile differences. | Social Skills | Cross functional Skills | 0 |
| Operation Monitoring | Watching gauges, dials, or other indicators to make sure a machine is working properly. | Technical Skills | Cross Functional Skills | 3.431 |
| Operation and Control | Controlling operations of equipment or systems. | Technical Skills | Cross Functional Skills | -0.438 |
| Operations Analysis | Analyzing needs and product requirements to create a design. | Technical Skills | Cross Functional Skills | 0 |
| Persuasion | Persuading others to change their minds or behavior. | Social Skills | Cross functional Skills | -0.827 |
| Programming | Writing computer programs for various purposes. | Technical Skills | Cross functional Skills | -0.904 |
| Quality Control Analysis | Conducting tests and inspections of products, services, or processes to evaluate quality or performance. | Technical Skills | Cross functional Skills | 0.191 |
| Reading Comprehension | Understanding written sentences and paragraphs in work related documents. | Content | Basic Skills | -0.707 |
| Repairing | Repairing machines or systems using the needed tools. | Technical Skills | Cross Functional Skills | -1.118 |
| Science | Using scientific rules and methods to solve problems. | Content | Basic Skills | 0 |
| Service Orientation | Actively looking for ways to help people. | Social Skills | Cross Functional Skills | 1.152 |
| Social Perceptiveness | Being aware of others' reactions and understanding why they react as they do. | Social Skills | Cross Functional Skills | -1.313 |
| Speaking | Talking to others to convey information effectively. | Content | Basic Skills | 2.367 |
| System Analysis | Determining how a system should work and how changes in conditions, operations, and the environment will affect outcomes. | Systems Skills | Cross Functional Skills | -1.486 |
| System Evaluation | Identifying measures or indicators of system performance and the actions needed to improve or correct performance, relative to the goals of the system. | Systems Skills | Cross Functional Skills | 5.622 |
| Technology Design | Generating or adapting equipment and technology to serve user needs. | Technical Skills | Cross Functional Skills | -2.925 |
| Time Management | Managing one's own time and the time of others. | Resource Management Skills | Cross Functional Skills | 2.661 |
| Troubleshooting | Determining causes of operating errors and deciding what to do about it. | Technical Skills | Cross Functional Skills | 0 |
| Writing | Communicating effectively in writing as appropriate for the needs of the audience. | Content | Basic Skills | 2.767 |
| R squared = 0.80 |  |  | N= | 16286 |

Table O3 Iceland People, Brains, Brawn Analysis

| \|  \| Iceland – EU LFS \| \| --- \| --- \| \| People \| 0.017*** \| \|  \| (0.001) \| \| Brains \| -0.067*** \| \|  \| (0.002) \| \| Brawn \| 0.014*** \| \|  \| (0.002) \| \| People * Brains \| -0.006*** \| \|  \| (0.001) \| \| People*Brawn \| -0.003*** \| \|  \| (0.001) \| \| Brains*Brawn \| -0.001 \| \|  \| (0.001) \| \| N \| 16245 \| \| R Squared \| 15% \| |
| --- | --- | --- | --- | --- | --- | --- | --- | --- | --- | --- | --- | --- | --- | --- | --- | --- | --- | --- | --- | --- | --- | --- | --- | --- | --- | --- | --- | --- | --- | --- |

***, **, * denotes significance at the 1%, 5% and 10% levels respectively

S18 Appendix. Individual country Italy

Table P1 Italy Ability Lasso Analysis

| ONET Item | Description |  |  | Coefficient |
| --- | --- | --- | --- | --- |
| Arm-Hand Steadiness | The ability to keep your hand and arm steady while moving your arm or while holding your arm and hand in one position. | Fine manipulative abilities | Psychomotor abilities | -1.455 |
| Auditory Attention | The ability to focus on a single source of sound in the presence of other distracting sounds. | Auditory and speech abilities | Sensory Abilities | -0.805 |
| Category Flexibility | The ability to generate or use different sets of rules for combining or grouping things in different ways. | Idea Generation and Reasoning Abilities | Cognitive Abilities | 0 |
| Control Precision | The ability to quickly and repeatedly adjust the controls of a machine or a vehicle to exact positions. | Control Movement Abilities | Psychomotor Abilities | 0 |
| Deductive Reasoning | The ability to apply general rules to specific problems to produce answers that make sense. | Idea Generation and Reasoning Abilities | Cognitive Abilities | -7.702 |
| Depth Perception | The ability to judge which of several objects is closer or farther away from you, or to judge the distance between you and an object. | Visual Abilities | Sensory Abilities | 2.498 |
| Dynamic Flexibility | The ability to quickly and repeatedly bend, stretch, twist, or reach out with your body, arms, and/or legs. | Flexibility, Balance and Coordination | Physical Abilities | 4.932 |
| Dynamic Strength | The ability to exert muscle force repeatedly or continuously over time. This involves muscular endurance and resistance to muscle fatigue. | Physical Strength | Physical Abilities | -2.037 |
| Explosive Strength | The ability to use short bursts of muscle force to propel oneself (as in jumping or sprinting), or to throw an object. | Physical Strength | Physical Abilities | 0.787 |
| Extent Flexibility | The ability to bend, stretch, twist, or reach with your body, arms, and/or legs. | Flexibility, balance and coordination | Physical Abilities | -1.298 |
| Far Vision | The ability to see details at a distance. | Visual Abilities | Sensory Abilities | -1.613 |
| Finger Dexterity | The ability to make precisely coordinated movements of the fingers of one or both hands to grasp, manipulate, or assemble very small objects. | Fine manipulative abilities | Psychomotor abilities | 0.94 |
| Flexibility of Closure | The ability to identify or detect a known pattern (a figure, object, word, or sound) that is hidden in other distracting material. | Perecptual Abilities | Cognitive abilities | 0 |
| Fluency of ideas | The ability to come up with a number of ideas about a topic (the number of ideas is important, not their quality, correctness, or creativity). | Ideas generation and reasoning abilities | Cognitive abilities | -1.148 |
| Glare sensitivity | The ability to see objects in the presence of glare or bright lighting. | Visual abilities | Sensory abilities | -2.648 |
| Gross Body Co-ordination | The ability to coordinate the movement of your arms, legs, and torso together when the whole body is in motion. | Flexibility, Balance and Coordination | Physical Abilities | 0 |
| Gross Body Equilibrium | The ability to keep or regain your body balance or stay upright when in an unstable position. | Flexibility, Balance and Coordination | Physical Abilities | 0 |
| Hearing Sensitivity | The ability to detect or tell the differences between sounds that vary in pitch and loudness. | Auditory and Speech abilities | Sensory Abilities | 0 |
| Inductive Reasoning | The ability to combine pieces of information to form general rules or conclusions (includes finding a relationship among seemingly unrelated events). | Idea Generation and Reasoning Abilities | Cognitive Abilities | 0 |
| Information Ordering | The ability to arrange things or actions in a certain order or pattern according to a specific rule or set of rules (e.g., patterns of numbers, letters, words, pictures, mathematical operations). | Idea Generation and Reasoning Abilities | Cognitive Abilities | 0 |
| Manual Dexterity | The ability to quickly move your hand, your hand together with your arm, or your two hands to grasp, manipulate, or assemble objects. | Fine Manipulative Abilities | Psychomotor Abilities | 1.354 |
| Math Reasoning | The ability to choose the right mathematical methods or formulas to solve a problem. | Quantitative Abilities | Cognitive Abilities | 2.864 |
| Memorization | Abilities related to the recall of available information | Quantitative Abilities | Cognitive Abilities | 1.211 |
| Multi Limb Co-ordination | The ability to coordinate two or more limbs (for example, two arms, two legs, or one leg and one arm) while sitting, standing, or lying down. It does not involve performing the activities while the whole body is in motion. | Control movement abilities | Psychomotor Abilities | 0.494 |
| Near Vision | The ability to see details at close range (within a few feet of the observer) | Near Vision | Visual Abilities | -1.212 |
| Night Vision | The ability to see under low light conditions | Near Vision | Visual Abilities | 2.115 |
| Number Facility | The ability to add, subtract, multiply, or divide quickly and correctly. | Quantitative Abilities | Cognitive Abilities | -2.786 |
| Oral Comprehension | The ability to listen to and understand information and ideas presented through spoken words and sentences. | Verbal Abilities | Cognitive Abilities | -2.669 |
| Oral Expression | The ability to communicate information and ideas in speaking so others will understand. | Verbal Abilities | Cognitive Abilities | 6.286 |
| Originality |  |  |  | 0 |
| Perceptual Speed | The ability to quickly and accurately compare similarities and differences among sets of letters, numbers, objects, pictures, or patterns. The things to be compared may be presented at the same time or one after the other. This ability also includes comparing a presented object with a remembered object. | Perceptual Abilities | Cognitive Abilities | -3.147 |
| Perceptual Vision | The ability to see objects or movement of objects to one's side when the eyes are looking ahead. | Visual Abilities | Sensory Abilities | -1.523 |
| Problem Sensitivity | The ability to tell when something is wrong or is likely to go wrong. It does not involve solving the problem, only recognizing there is a problem. | Idea generation and Reasoning Abilities | Cognitive Abilities | 2.313 |
| Rate Control | The ability to time your movements or the movement of a piece of equipment in anticipation of changes in the speed and/or direction of a moving object or scene. | Control Movement Abilities | Psychomotor Abilities | 3.301 |
| Reaction Time | The ability to quickly respond (with the hand, finger, or foot) to a signal (sound, light, picture) when it appears. | Reaction Time and Speed Abilities | Psychomotor Abilities | 1.054 |
| Response Orientation | The ability to choose quickly between two or more movements in response to two or more different signals (lights, sounds, pictures). It includes the speed with which the correct response is started with the hand, foot, or other body part. | Control Movement Abilities | Psychomotor Abilities | -2.881 |
| Selective Attention | The ability to concentrate on a task over a period of time without being distracted. | Attentiveness | Cognitive Abilities | 0 |
| Sound Localisation | The ability to tell the direction from which a sound originated. | Auditory and Speech Abilities | Sensory Abilities | 0 |
| Spatial Orientation | The ability to know your location in relation to the environment or to know where other objects are in relation to you. | Spatial Abilities | Cognitive Abilities | 1.621 |
| Speech Clarity | The ability to speak clearly so others can understand you. | Auditory and Speech Abilities | Sensory Abilities | 0 |
| Speech Recognition | The ability to identify and understand the speech of another person. | Auditory and Speech Abilities | Sensory Abilities | -1.986 |
| Speed of Closure | The ability to quickly make sense of, combine, and organize information into meaningful patterns. | Perceptual Abilities | Cognitive Abilities | 4.392 |
| Speed of Limb Movement | The ability to quickly move the arms and legs. | Reaction Time and Speed Abilities | Psychomotor Abilities | -2.839 |
| Stamina | The ability to exert yourself physically over long periods of time without getting winded or out of breath. | Endurance | Physical Abilities | 2.058 |
| Static Strength | The ability to exert maximum muscle force to lift, push, pull, or carry objects. | Physical Strength Abilities | Physical Abilities | 0.329 |
| Time Sharing | The ability to shift back and forth between two or more activities or sources of information (such as speech, sounds, touch, or other sources). | Attentiveness | Cognitive Abilities | 1.801 |
| Trunk Strength | The ability to use your abdominal and lower back muscles to support part of the body repeatedly or continuously over time without 'giving out' or fatiguing. | Physical Strength Abilities | Physical Abilities | -1.148 |
| Visual Color Discrimination | The ability to match or detect differences between colors, including shades of color and brightness. | Visual Abilities | Sensory Abilities | 0 |
| Visualisation | The ability to imagine how something will look after it is moved around or when its parts are moved or rearranged. | Spatial Abilities | Cognitive Abilities | -0.979 |
| Wrist-Finger Speed | The ability to make fast, simple, repeated movements of the fingers, hands, and wrists. | Reaction Time and Speed Abilities | Psychomotor Abilities | 0 |
| Written Comprehension | The ability to read and understand information and ideas presented in writing. | Verbal Abilities | Cognitive Abilities | 0.707 |
| Written Expression | The ability to communicate information and ideas in writing so others will understand. | Verbal Abilities | Cognitive Abilities | -1.431 |
| R Squared = 0.96 | |  | N= 337066 | |

Table P2 Italy Skills Lasso Analysis

| ONET Item | Description |  |  | Coefficient |
| --- | --- | --- | --- | --- |
| Active Learning | Understanding the implications of new information for both current and future problem-solving and decision-making. | Process | Basic Skills | 1.666 |
| Active Listening | Giving full attention to what other people are saying, taking time to understand the points being made, asking questions as appropriate, and not interrupting at inappropriate times. | Content | Basic Skills | -0.776 |
| Complex Problem Solving | Identifying complex problems and reviewing related information to develop and evaluate options and implement solutions. | Complex Problem Solving | Cross Functional Skills | 0 |
| Coordination | Adjusting actions in relation to others' actions. | Social Skills | Cross Functional Skills | -7.674 |
| Critical Thinking | Using logic and reasoning to identify the strengths and weaknesses of alternative solutions, conclusions or approaches to problems. | Process | Basic Skills | -3.374 |
| Equipment Maintenance | Performing routine maintenance on equipment and determining when and what kind of maintenance is needed. | Technical Skills | Cross Functional Skills | 0 |
| Equipment Selection | Determining the kind of tools and equipment needed to do a job. | Technical Skills | Cross Functional Skills | 1.503 |
| Installation | Installing equipment, machines, wiring, or programs to meet specifications. | Technical Skills | Cross Functional Skills | 0.844 |
| Instructing | Teaching others how to do something. | Social Skills | Cross Functional Skills | -2.769 |
| Judgement and Decision Making | Considering the relative costs and benefits of potential actions to choose the most appropriate one. | Systems Skills | Cross Functional Skills | 1.905 |
| Learning Strategies | Selecting and using training/instructional methods and procedures appropriate for the situation when learning or teaching new things. | Systems Skills | Cross Functional Skills | 0 |
| Management of Financial Resources | Determining how money will be spent to get the work done, and accounting for these expenditures. | Resource Management Skills | Cross functional Skills | 0 |
| Management of Material Resources | Obtaining and seeing to the appropriate use of equipment, facilities, and materials needed to do certain work. | Resource Management Skills | Cross functional Skills | -1.098 |
| Management of Personell Resources | Motivating, developing, and directing people as they work, identifying the best people for the job. | Resource Management Skills | Cross functional Skills | 1.727 |
| Mathematics | Using mathematics to solve problems. | Content | Basic Skills | 0 |
| Monitoring | Monitoring/Assessing performance of yourself, other individuals, or organizations to make improvements or take corrective action. | Process | Basic Skills | -5.162 |
| Negotiation | Bringing others together and trying to reconcile differences. | Social Skills | Cross functional Skills | 1.831 |
| Operation Monitoring | Watching gauges, dials, or other indicators to make sure a machine is working properly. | Technical Skills | Cross Functional Skills | 2.984 |
| Operation and Control | Controlling operations of equipment or systems. | Technical Skills | Cross Functional Skills | -0.647 |
| Operations Analysis | Analyzing needs and product requirements to create a design. | Technical Skills | Cross Functional Skills | -1.165 |
| Persuasion | Persuading others to change their minds or behavior. | Social Skills | Cross functional Skills | -0.13 |
| Programming | Writing computer programs for various purposes. | Technical Skills | Cross functional Skills | 0 |
| Quality Control Analysis | Conducting tests and inspections of products, services, or processes to evaluate quality or performance. | Technical Skills | Cross functional Skills | -0.729 |
| Reading Comprehension | Understanding written sentences and paragraphs in work related documents. | Content | Basic Skills | 0 |
| Repairing | Repairing machines or systems using the needed tools. | Technical Skills | Cross Functional Skills | -2.24 |
| Science | Using scientific rules and methods to solve problems. | Content | Basic Skills | 0.436 |
| Service Orientation | Actively looking for ways to help people. | Social Skills | Cross Functional Skills | -0.988 |
| Social Perceptiveness | Being aware of others' reactions and understanding why they react as they do. | Social Skills | Cross Functional Skills | 0 |
| Speaking | Talking to others to convey information effectively. | Content | Basic Skills | 2.647 |
| System Analysis | Determining how a system should work and how changes in conditions, operations, and the environment will affect outcomes. | Systems Skills | Cross Functional Skills | -2.856 |
| System Evaluation | Identifying measures or indicators of system performance and the actions needed to improve or correct performance, relative to the goals of the system. | Systems Skills | Cross Functional Skills | 5.888 |
| Technology Design | Generating or adapting equipment and technology to serve user needs. | Technical Skills | Cross Functional Skills | -3.437 |
| Time Management | Managing one's own time and the time of others. | Resource Management Skills | Cross Functional Skills | 4.727 |
| Troubleshooting | Determining causes of operating errors and deciding what to do about it. | Technical Skills | Cross Functional Skills | 0 |
| Writing | Communicating effectively in writing as appropriate for the needs of the audience. | Content | Basic Skills | 0 |
| R squared = 0.80 |  |  | N= | 337066 |

Table P3 Italy People, Brains, Brawn Analysis

| \|  \| Italy – EU LFS \| \| --- \| --- \| \| People \| 0.027*** \| \|  \| (0.000) \| \| Brains \| -0.054*** \| \|  \| (0.000) \| \| Brawn \| 0.066*** \| \|  \| (0.001) \| \| People * Brains \| -0.004*** \| \|  \| (0.000) \| \| People*Brawn \| 0.005*** \| \|  \| (0.000) \| \| Brains*Brawn \| -0.015*** \| \|  \| (0.000) \| \| N \| 336971 \| \| R Squared \| 11% \| |
| --- | --- | --- | --- | --- | --- | --- | --- | --- | --- | --- | --- | --- | --- | --- | --- | --- | --- | --- | --- | --- | --- | --- | --- | --- | --- | --- | --- | --- | --- | --- |

***, **, * denotes significance at the 1%, 5% and 10% levels respectively

S19 Appendix. Individual country Luxemburg

Table Q1 Luxemburg Ability Lasso Analysis

| ONET Item | Description |  |  | Coefficient |
| --- | --- | --- | --- | --- |
| Arm-Hand Steadiness | The ability to keep your hand and arm steady while moving your arm or while holding your arm and hand in one position. | Fine manipulative abilities | Psychomotor abilities | -3.527 |
| Auditory Attention | The ability to focus on a single source of sound in the presence of other distracting sounds. | Auditory and speech abilities | Sensory Abilities | 0 |
| Category Flexibility | The ability to generate or use different sets of rules for combining or grouping things in different ways. | Idea Generation and Reasoning Abilities | Cognitive Abilities | -3.47 |
| Control Precision | The ability to quickly and repeatedly adjust the controls of a machine or a vehicle to exact positions. | Control Movement Abilities | Psychomotor Abilities | 1.959 |
| Deductive Reasoning | The ability to apply general rules to specific problems to produce answers that make sense. | Idea Generation and Reasoning Abilities | Cognitive Abilities | -4.73 |
| Depth Perception | The ability to judge which of several objects is closer or farther away from you, or to judge the distance between you and an object. | Visual Abilities | Sensory Abilities | 0 |
| Dynamic Flexibility | The ability to quickly and repeatedly bend, stretch, twist, or reach out with your body, arms, and/or legs. | Flexibility, Balance and Coordination | Physical Abilities | 5.074 |
| Dynamic Strength | The ability to exert muscle force repeatedly or continuously over time. This involves muscular endurance and resistance to muscle fatigue. | Physical Strength | Physical Abilities | -1.391 |
| Explosive Strength | The ability to use short bursts of muscle force to propel oneself (as in jumping or sprinting), or to throw an object. | Physical Strength | Physical Abilities | 0.89 |
| Extent Flexibility | The ability to bend, stretch, twist, or reach with your body, arms, and/or legs. | Flexibility, balance and coordination | Physical Abilities | -2.651 |
| Far Vision | The ability to see details at a distance. | Visual Abilities | Sensory Abilities | -2.614 |
| Finger Dexterity | The ability to make precisely coordinated movements of the fingers of one or both hands to grasp, manipulate, or assemble very small objects. | Fine manipulative abilities | Psychomotor abilities | 1.965 |
| Flexibility of Closure | The ability to identify or detect a known pattern (a figure, object, word, or sound) that is hidden in other distracting material. | Perecptual Abilities | Cognitive abilities | 0 |
| Fluency of ideas | The ability to come up with a number of ideas about a topic (the number of ideas is important, not their quality, correctness, or creativity). | Ideas generation and reasoning abilities | Cognitive abilities | -2.728 |
| Glare sensitivity | The ability to see objects in the presence of glare or bright lighting. | Visual abilities | Sensory abilities | -3.102 |
| Gross Body Co-ordination | The ability to coordinate the movement of your arms, legs, and torso together when the whole body is in motion. | Flexibility, Balance and Coordination | Physical Abilities | 0 |
| Gross Body Equilibrium | The ability to keep or regain your body balance or stay upright when in an unstable position. | Flexibility, Balance and Coordination | Physical Abilities | -0.016 |
| Hearing Sensitivity | The ability to detect or tell the differences between sounds that vary in pitch and loudness. | Auditory and Speech abilities | Sensory Abilities | 0 |
| Inductive Reasoning | The ability to combine pieces of information to form general rules or conclusions (includes finding a relationship among seemingly unrelated events). | Idea Generation and Reasoning Abilities | Cognitive Abilities | 0 |
| Information Ordering | The ability to arrange things or actions in a certain order or pattern according to a specific rule or set of rules (e.g., patterns of numbers, letters, words, pictures, mathematical operations). | Idea Generation and Reasoning Abilities | Cognitive Abilities | 0 |
| Manual Dexterity | The ability to quickly move your hand, your hand together with your arm, or your two hands to grasp, manipulate, or assemble objects. | Fine Manipulative Abilities | Psychomotor Abilities | 0 |
| Math Reasoning | The ability to choose the right mathematical methods or formulas to solve a problem. | Quantitative Abilities | Cognitive Abilities | 0 |
| Memorization | Abilities related to the recall of available information | Quantitative Abilities | Cognitive Abilities | 0.373 |
| Multi Limb Co-ordination | The ability to coordinate two or more limbs (for example, two arms, two legs, or one leg and one arm) while sitting, standing, or lying down. It does not involve performing the activities while the whole body is in motion. | Control movement abilities | Psychomotor Abilities | 1.1 |
| Near Vision | The ability to see details at close range (within a few feet of the observer) | Near Vision | Visual Abilities | 0.053 |
| Night Vision | The ability to see under low light conditions | Near Vision | Visual Abilities | 0 |
| Number Facility | The ability to add, subtract, multiply, or divide quickly and correctly. | Quantitative Abilities | Cognitive Abilities | 0 |
| Oral Comprehension | The ability to listen to and understand information and ideas presented through spoken words and sentences. | Verbal Abilities | Cognitive Abilities | 1.854 |
| Oral Expression | The ability to communicate information and ideas in speaking so others will understand. | Verbal Abilities | Cognitive Abilities | 3.828 |
| Originality |  |  |  | -0.48 |
| Perceptual Speed | The ability to quickly and accurately compare similarities and differences among sets of letters, numbers, objects, pictures, or patterns. The things to be compared may be presented at the same time or one after the other. This ability also includes comparing a presented object with a remembered object. | Perceptual Abilities | Cognitive Abilities | -4.04 |
| Perceptual Vision | The ability to see objects or movement of objects to one's side when the eyes are looking ahead. | Visual Abilities | Sensory Abilities | 0.948 |
| Problem Sensitivity | The ability to tell when something is wrong or is likely to go wrong. It does not involve solving the problem, only recognizing there is a problem. | Idea generation and Reasoning Abilities | Cognitive Abilities | 1.572 |
| Rate Control | The ability to time your movements or the movement of a piece of equipment in anticipation of changes in the speed and/or direction of a moving object or scene. | Control Movement Abilities | Psychomotor Abilities | 3.985 |
| Reaction Time | The ability to quickly respond (with the hand, finger, or foot) to a signal (sound, light, picture) when it appears. | Reaction Time and Speed Abilities | Psychomotor Abilities | 1.288 |
| Response Orientation | The ability to choose quickly between two or more movements in response to two or more different signals (lights, sounds, pictures). It includes the speed with which the correct response is started with the hand, foot, or other body part. | Control Movement Abilities | Psychomotor Abilities | -3.859 |
| Selective Attention | The ability to concentrate on a task over a period of time without being distracted. | Attentiveness | Cognitive Abilities | -0.58 |
| Sound Localisation | The ability to tell the direction from which a sound originated. | Auditory and Speech Abilities | Sensory Abilities | 0 |
| Spatial Orientation | The ability to know your location in relation to the environment or to know where other objects are in relation to you. | Spatial Abilities | Cognitive Abilities | 1.291 |
| Speech Clarity | The ability to speak clearly so others can understand you. | Auditory and Speech Abilities | Sensory Abilities | 0 |
| Speech Recognition | The ability to identify and understand the speech of another person. | Auditory and Speech Abilities | Sensory Abilities | 0 |
| Speed of Closure | The ability to quickly make sense of, combine, and organize information into meaningful patterns. | Perceptual Abilities | Cognitive Abilities | 4.811 |
| Speed of Limb Movement | The ability to quickly move the arms and legs. | Reaction Time and Speed Abilities | Psychomotor Abilities | 0 |
| Stamina | The ability to exert yourself physically over long periods of time without getting winded or out of breath. | Endurance | Physical Abilities | 0.473 |
| Static Strength | The ability to exert maximum muscle force to lift, push, pull, or carry objects. | Physical Strength Abilities | Physical Abilities | 1.374 |
| Time Sharing | The ability to shift back and forth between two or more activities or sources of information (such as speech, sounds, touch, or other sources). | Attentiveness | Cognitive Abilities | 0 |
| Trunk Strength | The ability to use your abdominal and lower back muscles to support part of the body repeatedly or continuously over time without 'giving out' or fatiguing. | Physical Strength Abilities | Physical Abilities | 0 |
| Visual Color Discrimination | The ability to match or detect differences between colors, including shades of color and brightness. | Visual Abilities | Sensory Abilities | 4.394 |
| Visualisation | The ability to imagine how something will look after it is moved around or when its parts are moved or rearranged. | Spatial Abilities | Cognitive Abilities | -0.671 |
| Wrist-Finger Speed | The ability to make fast, simple, repeated movements of the fingers, hands, and wrists. | Reaction Time and Speed Abilities | Psychomotor Abilities | -1.536 |
| Written Comprehension | The ability to read and understand information and ideas presented in writing. | Verbal Abilities | Cognitive Abilities | -0.359 |
| Written Expression | The ability to communicate information and ideas in writing so others will understand. | Verbal Abilities | Cognitive Abilities | 0 |
| R Squared = 0.97 | |  | N=17383 | |

Table Q2 Luxemburg Skills Lasso Analysis

| ONET Item | Description |  |  | Coefficient |
| --- | --- | --- | --- | --- |
| Active Learning | Understanding the implications of new information for both current and future problem-solving and decision-making. | Process | Basic Skills | 0 |
| Active Listening | Giving full attention to what other people are saying, taking time to understand the points being made, asking questions as appropriate, and not interrupting at inappropriate times. | Content | Basic Skills | -1.051 |
| Complex Problem Solving | Identifying complex problems and reviewing related information to develop and evaluate options and implement solutions. | Complex Problem Solving | Cross Functional Skills | -5.366 |
| Coordination | Adjusting actions in relation to others' actions. | Social Skills | Cross Functional Skills | -3.578 |
| Critical Thinking | Using logic and reasoning to identify the strengths and weaknesses of alternative solutions, conclusions or approaches to problems. | Process | Basic Skills | -3.552 |
| Equipment Maintenance | Performing routine maintenance on equipment and determining when and what kind of maintenance is needed. | Technical Skills | Cross Functional Skills | -0.889 |
| Equipment Selection | Determining the kind of tools and equipment needed to do a job. | Technical Skills | Cross Functional Skills | 0.33 |
| Installation | Installing equipment, machines, wiring, or programs to meet specifications. | Technical Skills | Cross Functional Skills | 0 |
| Instructing | Teaching others how to do something. | Social Skills | Cross Functional Skills | -1.957 |
| Judgement and Decision Making | Considering the relative costs and benefits of potential actions to choose the most appropriate one. | Systems Skills | Cross Functional Skills | 4.94 |
| Learning Strategies | Selecting and using training/instructional methods and procedures appropriate for the situation when learning or teaching new things. | Systems Skills | Cross Functional Skills | 1.248 |
| Management of Financial Resources | Determining how money will be spent to get the work done, and accounting for these expenditures. | Resource Management Skills | Cross functional Skills | 0.449 |
| Management of Material Resources | Obtaining and seeing to the appropriate use of equipment, facilities, and materials needed to do certain work. | Resource Management Skills | Cross functional Skills | 0 |
| Management of Personell Resources | Motivating, developing, and directing people as they work, identifying the best people for the job. | Resource Management Skills | Cross functional Skills | 0 |
| Mathematics | Using mathematics to solve problems. | Content | Basic Skills | -1.016 |
| Monitoring | Monitoring/Assessing performance of yourself, other individuals, or organizations to make improvements or take corrective action. | Process | Basic Skills | -5.584 |
| Negotiation | Bringing others together and trying to reconcile differences. | Social Skills | Cross functional Skills | 0.907 |
| Operation Monitoring | Watching gauges, dials, or other indicators to make sure a machine is working properly. | Technical Skills | Cross Functional Skills | 3.127 |
| Operation and Control | Controlling operations of equipment or systems. | Technical Skills | Cross Functional Skills | 0 |
| Operations Analysis | Analyzing needs and product requirements to create a design. | Technical Skills | Cross Functional Skills | 0 |
| Persuasion | Persuading others to change their minds or behavior. | Social Skills | Cross functional Skills | 0 |
| Programming | Writing computer programs for various purposes. | Technical Skills | Cross functional Skills | -0.442 |
| Quality Control Analysis | Conducting tests and inspections of products, services, or processes to evaluate quality or performance. | Technical Skills | Cross functional Skills | -1.321 |
| Reading Comprehension | Understanding written sentences and paragraphs in work related documents. | Content | Basic Skills | 0.992 |
| Repairing | Repairing machines or systems using the needed tools. | Technical Skills | Cross Functional Skills | -0.575 |
| Science | Using scientific rules and methods to solve problems. | Content | Basic Skills | 0.838 |
| Service Orientation | Actively looking for ways to help people. | Social Skills | Cross Functional Skills | -0.655 |
| Social Perceptiveness | Being aware of others' reactions and understanding why they react as they do. | Social Skills | Cross Functional Skills | -1.296 |
| Speaking | Talking to others to convey information effectively. | Content | Basic Skills | 1.859 |
| System Analysis | Determining how a system should work and how changes in conditions, operations, and the environment will affect outcomes. | Systems Skills | Cross Functional Skills | -1.634 |
| System Evaluation | Identifying measures or indicators of system performance and the actions needed to improve or correct performance, relative to the goals of the system. | Systems Skills | Cross Functional Skills | 4.382 |
| Technology Design | Generating or adapting equipment and technology to serve user needs. | Technical Skills | Cross Functional Skills | -2.029 |
| Time Management | Managing one's own time and the time of others. | Resource Management Skills | Cross Functional Skills | 3.264 |
| Troubleshooting | Determining causes of operating errors and deciding what to do about it. | Technical Skills | Cross Functional Skills | 0.448 |
| Writing | Communicating effectively in writing as appropriate for the needs of the audience. | Content | Basic Skills | 1.578 |
| R squared = 0.82 |  |  | N= | 17383 |

Table Q3 Luxemburg People, Brains, Brawn Analysis

|  | |
| --- | --- |
|  | Luxemburg – EU LFS |
| People | 0.012*** |
|  | (0.001) |
| Brains | -0.051*** |
|  | (0.001) |
| Brawn | 0.029*** |
|  | (0.001) |
| People * Brains | 0.000*** |
|  | (0.000) |
| People*Brawn | 0.000*** |
|  | (0.000) |
| Brains*Brawn | -0.000 |
|  | (0.000) |
| N | 18867 |
| R Squared | 8% |

***, **, * denotes significance at the 1%, 5% and 10% levels respectively

S20 Appendix. Individual country Latvia

Table R 1 Latvia Ability Lasso Analysis

| ONET Item | Description |  |  | Coefficient |
| --- | --- | --- | --- | --- |
| Arm-Hand Steadiness | The ability to keep your hand and arm steady while moving your arm or while holding your arm and hand in one position. | Fine manipulative abilities | Psychomotor abilities | -2.086 |
| Auditory Attention | The ability to focus on a single source of sound in the presence of other distracting sounds. | Auditory and speech abilities | Sensory Abilities | 0.581 |
| Category Flexibility | The ability to generate or use different sets of rules for combining or grouping things in different ways. | Idea Generation and Reasoning Abilities | Cognitive Abilities | -2.172 |
| Control Precision | The ability to quickly and repeatedly adjust the controls of a machine or a vehicle to exact positions. | Control Movement Abilities | Psychomotor Abilities | 1.305 |
| Deductive Reasoning | The ability to apply general rules to specific problems to produce answers that make sense. | Idea Generation and Reasoning Abilities | Cognitive Abilities | 0 |
| Depth Perception | The ability to judge which of several objects is closer or farther away from you, or to judge the distance between you and an object. | Visual Abilities | Sensory Abilities | 0.308 |
| Dynamic Flexibility | The ability to quickly and repeatedly bend, stretch, twist, or reach out with your body, arms, and/or legs. | Flexibility, Balance and Coordination | Physical Abilities | 2.384 |
| Dynamic Strength | The ability to exert muscle force repeatedly or continuously over time. This involves muscular endurance and resistance to muscle fatigue. | Physical Strength | Physical Abilities | 0 |
| Explosive Strength | The ability to use short bursts of muscle force to propel oneself (as in jumping or sprinting), or to throw an object. | Physical Strength | Physical Abilities | 0 |
| Extent Flexibility | The ability to bend, stretch, twist, or reach with your body, arms, and/or legs. | Flexibility, balance and coordination | Physical Abilities | -1.981 |
| Far Vision | The ability to see details at a distance. | Visual Abilities | Sensory Abilities | -3.949 |
| Finger Dexterity | The ability to make precisely coordinated movements of the fingers of one or both hands to grasp, manipulate, or assemble very small objects. | Fine manipulative abilities | Psychomotor abilities | 3.547 |
| Flexibility of Closure | The ability to identify or detect a known pattern (a figure, object, word, or sound) that is hidden in other distracting material. | Perecptual Abilities | Cognitive abilities | 0 |
| Fluency of ideas | The ability to come up with a number of ideas about a topic (the number of ideas is important, not their quality, correctness, or creativity). | Ideas generation and reasoning abilities | Cognitive abilities | -4.547 |
| Glare sensitivity | The ability to see objects in the presence of glare or bright lighting. | Visual abilities | Sensory abilities | -3.105 |
| Gross Body Co-ordination | The ability to coordinate the movement of your arms, legs, and torso together when the whole body is in motion. | Flexibility, Balance and Coordination | Physical Abilities | 0 |
| Gross Body Equilibrium | The ability to keep or regain your body balance or stay upright when in an unstable position. | Flexibility, Balance and Coordination | Physical Abilities | -1.106 |
| Hearing Sensitivity | The ability to detect or tell the differences between sounds that vary in pitch and loudness. | Auditory and Speech abilities | Sensory Abilities | -1.17 |
| Inductive Reasoning | The ability to combine pieces of information to form general rules or conclusions (includes finding a relationship among seemingly unrelated events). | Idea Generation and Reasoning Abilities | Cognitive Abilities | -0.957 |
| Information Ordering | The ability to arrange things or actions in a certain order or pattern according to a specific rule or set of rules (e.g., patterns of numbers, letters, words, pictures, mathematical operations). | Idea Generation and Reasoning Abilities | Cognitive Abilities | 0 |
| Manual Dexterity | The ability to quickly move your hand, your hand together with your arm, or your two hands to grasp, manipulate, or assemble objects. | Fine Manipulative Abilities | Psychomotor Abilities | -2.368 |
| Math Reasoning | The ability to choose the right mathematical methods or formulas to solve a problem. | Quantitative Abilities | Cognitive Abilities | 0 |
| Memorization | Abilities related to the recall of available information | Quantitative Abilities | Cognitive Abilities | 0 |
| Multi Limb Co-ordination | The ability to coordinate two or more limbs (for example, two arms, two legs, or one leg and one arm) while sitting, standing, or lying down. It does not involve performing the activities while the whole body is in motion. | Control movement abilities | Psychomotor Abilities | 1.119 |
| Near Vision | The ability to see details at close range (within a few feet of the observer) | Near Vision | Visual Abilities | 0.812 |
| Night Vision | The ability to see under low light conditions | Near Vision | Visual Abilities | 1.643 |
| Number Facility | The ability to add, subtract, multiply, or divide quickly and correctly. | Quantitative Abilities | Cognitive Abilities | -1.379 |
| Oral Comprehension | The ability to listen to and understand information and ideas presented through spoken words and sentences. | Verbal Abilities | Cognitive Abilities | 0 |
| Oral Expression | The ability to communicate information and ideas in speaking so others will understand. | Verbal Abilities | Cognitive Abilities | 2.443 |
| Originality |  |  |  | 0 |
| Perceptual Speed | The ability to quickly and accurately compare similarities and differences among sets of letters, numbers, objects, pictures, or patterns. The things to be compared may be presented at the same time or one after the other. This ability also includes comparing a presented object with a remembered object. | Perceptual Abilities | Cognitive Abilities | -4.162 |
| Perceptual Vision | The ability to see objects or movement of objects to one's side when the eyes are looking ahead. | Visual Abilities | Sensory Abilities | 3.876 |
| Problem Sensitivity | The ability to tell when something is wrong or is likely to go wrong. It does not involve solving the problem, only recognizing there is a problem. | Idea generation and Reasoning Abilities | Cognitive Abilities | 1.221 |
| Rate Control | The ability to time your movements or the movement of a piece of equipment in anticipation of changes in the speed and/or direction of a moving object or scene. | Control Movement Abilities | Psychomotor Abilities | 3.423 |
| Reaction Time | The ability to quickly respond (with the hand, finger, or foot) to a signal (sound, light, picture) when it appears. | Reaction Time and Speed Abilities | Psychomotor Abilities | 0 |
| Response Orientation | The ability to choose quickly between two or more movements in response to two or more different signals (lights, sounds, pictures). It includes the speed with which the correct response is started with the hand, foot, or other body part. | Control Movement Abilities | Psychomotor Abilities | -3.749 |
| Selective Attention | The ability to concentrate on a task over a period of time without being distracted. | Attentiveness | Cognitive Abilities | 0.774 |
| Sound Localisation | The ability to tell the direction from which a sound originated. | Auditory and Speech Abilities | Sensory Abilities | -2.89 |
| Spatial Orientation | The ability to know your location in relation to the environment or to know where other objects are in relation to you. | Spatial Abilities | Cognitive Abilities | 1.797 |
| Speech Clarity | The ability to speak clearly so others can understand you. | Auditory and Speech Abilities | Sensory Abilities | 0.837 |
| Speech Recognition | The ability to identify and understand the speech of another person. | Auditory and Speech Abilities | Sensory Abilities | 0 |
| Speed of Closure | The ability to quickly make sense of, combine, and organize information into meaningful patterns. | Perceptual Abilities | Cognitive Abilities | 5.165 |
| Speed of Limb Movement | The ability to quickly move the arms and legs. | Reaction Time and Speed Abilities | Psychomotor Abilities | -0.195 |
| Stamina | The ability to exert yourself physically over long periods of time without getting winded or out of breath. | Endurance | Physical Abilities | 1.254 |
| Static Strength | The ability to exert maximum muscle force to lift, push, pull, or carry objects. | Physical Strength Abilities | Physical Abilities | 1.329 |
| Time Sharing | The ability to shift back and forth between two or more activities or sources of information (such as speech, sounds, touch, or other sources). | Attentiveness | Cognitive Abilities | 0 |
| Trunk Strength | The ability to use your abdominal and lower back muscles to support part of the body repeatedly or continuously over time without 'giving out' or fatiguing. | Physical Strength Abilities | Physical Abilities | 0 |
| Visual Color Discrimination | The ability to match or detect differences between colors, including shades of color and brightness. | Visual Abilities | Sensory Abilities | 5.061 |
| Visualisation | The ability to imagine how something will look after it is moved around or when its parts are moved or rearranged. | Spatial Abilities | Cognitive Abilities | -1.039 |
| Wrist-Finger Speed | The ability to make fast, simple, repeated movements of the fingers, hands, and wrists. | Reaction Time and Speed Abilities | Psychomotor Abilities | 0 |
| Written Comprehension | The ability to read and understand information and ideas presented in writing. | Verbal Abilities | Cognitive Abilities | 0.535 |
| Written Expression | The ability to communicate information and ideas in writing so others will understand. | Verbal Abilities | Cognitive Abilities | 0 |
| R Squared = 0.98 | |  | N=32177 | |

Table R2 Latvia Skills Lasso Analysis

| ONET Item | Description |  |  | Coefficient |
| --- | --- | --- | --- | --- |
| Active Learning | Understanding the implications of new information for both current and future problem-solving and decision-making. | Process | Basic Skills | 2.028 |
| Active Listening | Giving full attention to what other people are saying, taking time to understand the points being made, asking questions as appropriate, and not interrupting at inappropriate times. | Content | Basic Skills | -1.141 |
| Complex Problem Solving | Identifying complex problems and reviewing related information to develop and evaluate options and implement solutions. | Complex Problem Solving | Cross Functional Skills | -0.491 |
| Coordination | Adjusting actions in relation to others' actions. | Social Skills | Cross Functional Skills | -3.967 |
| Critical Thinking | Using logic and reasoning to identify the strengths and weaknesses of alternative solutions, conclusions or approaches to problems. | Process | Basic Skills | -2.057 |
| Equipment Maintenance | Performing routine maintenance on equipment and determining when and what kind of maintenance is needed. | Technical Skills | Cross Functional Skills | -0.332 |
| Equipment Selection | Determining the kind of tools and equipment needed to do a job. | Technical Skills | Cross Functional Skills | 1.593 |
| Installation | Installing equipment, machines, wiring, or programs to meet specifications. | Technical Skills | Cross Functional Skills | 0.113 |
| Instructing | Teaching others how to do something. | Social Skills | Cross Functional Skills | -2.997 |
| Judgement and Decision Making | Considering the relative costs and benefits of potential actions to choose the most appropriate one. | Systems Skills | Cross Functional Skills | 2.683 |
| Learning Strategies | Selecting and using training/instructional methods and procedures appropriate for the situation when learning or teaching new things. | Systems Skills | Cross Functional Skills | 0 |
| Management of Financial Resources | Determining how money will be spent to get the work done, and accounting for these expenditures. | Resource Management Skills | Cross functional Skills | 0 |
| Management of Material Resources | Obtaining and seeing to the appropriate use of equipment, facilities, and materials needed to do certain work. | Resource Management Skills | Cross functional Skills | -0.336 |
| Management of Personell Resources | Motivating, developing, and directing people as they work, identifying the best people for the job. | Resource Management Skills | Cross functional Skills | 0 |
| Mathematics | Using mathematics to solve problems. | Content | Basic Skills | 0 |
| Monitoring | Monitoring/Assessing performance of yourself, other individuals, or organizations to make improvements or take corrective action. | Process | Basic Skills | -3.267 |
| Negotiation | Bringing others together and trying to reconcile differences. | Social Skills | Cross functional Skills | 3.268 |
| Operation Monitoring | Watching gauges, dials, or other indicators to make sure a machine is working properly. | Technical Skills | Cross Functional Skills | 2.202 |
| Operation and Control | Controlling operations of equipment or systems. | Technical Skills | Cross Functional Skills | 0 |
| Operations Analysis | Analyzing needs and product requirements to create a design. | Technical Skills | Cross Functional Skills | -1.318 |
| Persuasion | Persuading others to change their minds or behavior. | Social Skills | Cross functional Skills | -1.673 |
| Programming | Writing computer programs for various purposes. | Technical Skills | Cross functional Skills | 0.642 |
| Quality Control Analysis | Conducting tests and inspections of products, services, or processes to evaluate quality or performance. | Technical Skills | Cross functional Skills | -1.817 |
| Reading Comprehension | Understanding written sentences and paragraphs in work related documents. | Content | Basic Skills | -0.755 |
| Repairing | Repairing machines or systems using the needed tools. | Technical Skills | Cross Functional Skills | -1.102 |
| Science | Using scientific rules and methods to solve problems. | Content | Basic Skills | 0.767 |
| Service Orientation | Actively looking for ways to help people. | Social Skills | Cross Functional Skills | 0 |
| Social Perceptiveness | Being aware of others' reactions and understanding why they react as they do. | Social Skills | Cross Functional Skills | 0.349 |
| Speaking | Talking to others to convey information effectively. | Content | Basic Skills | 0 |
| System Analysis | Determining how a system should work and how changes in conditions, operations, and the environment will affect outcomes. | Systems Skills | Cross Functional Skills | 0 |
| System Evaluation | Identifying measures or indicators of system performance and the actions needed to improve or correct performance, relative to the goals of the system. | Systems Skills | Cross Functional Skills | 2.696 |
| Technology Design | Generating or adapting equipment and technology to serve user needs. | Technical Skills | Cross Functional Skills | -2.982 |
| Time Management | Managing one's own time and the time of others. | Resource Management Skills | Cross Functional Skills | 3.779 |
| Troubleshooting | Determining causes of operating errors and deciding what to do about it. | Technical Skills | Cross Functional Skills | 0 |
| Writing | Communicating effectively in writing as appropriate for the needs of the audience. | Content | Basic Skills | 0 |
| R squared = 0.85 |  |  | N= | 32177 |

Table R3 Latvia People, Brains, Brawn Analysis

|  | Latvia – EU LFS |
| --- | --- |
| People | 0.056*** |
|  | (0.001) |
| Brains | -0.185*** |
|  | (0.002) |
| Brawn | 0.041*** |
|  | (0.002) |
| People * Brains | -0.002*** |
|  | (0.001) |
| People*Brawn | -0.010*** |
|  | (0.001) |
| Brains*Brawn | -0.072*** |
|  | (0.001) |
| N | 32147 |
| R Squared | 36% |

***, **, * denotes significance at the 1%, 5% and 10% levels respectively

S21 Appendix. Individual country Netherlands

Table S1 Netherlands Ability Lasso Analysis

| ONET Item | Description |  |  | Coefficient |
| --- | --- | --- | --- | --- |
| Arm-Hand Steadiness | The ability to keep your hand and arm steady while moving your arm or while holding your arm and hand in one position. | Fine manipulative abilities | -3.538 | -3.538 |
| Auditory Attention | The ability to focus on a single source of sound in the presence of other distracting sounds. | Auditory and speech abilities | 0 | 0 |
| Category Flexibility | The ability to generate or use different sets of rules for combining or grouping things in different ways. | Idea Generation and Reasoning Abilities | -2.003 | -2.003 |
| Control Precision | The ability to quickly and repeatedly adjust the controls of a machine or a vehicle to exact positions. | Control Movement Abilities | 2.085 | 2.085 |
| Deductive Reasoning | The ability to apply general rules to specific problems to produce answers that make sense. | Idea Generation and Reasoning Abilities | -6.952 | -6.952 |
| Depth Perception | The ability to judge which of several objects is closer or farther away from you, or to judge the distance between you and an object. | Visual Abilities | 0 | 0 |
| Dynamic Flexibility | The ability to quickly and repeatedly bend, stretch, twist, or reach out with your body, arms, and/or legs. | Flexibility, Balance and Coordination | 3.815 | 3.815 |
| Dynamic Strength | The ability to exert muscle force repeatedly or continuously over time. This involves muscular endurance and resistance to muscle fatigue. | Physical Strength | 0 | 0 |
| Explosive Strength | The ability to use short bursts of muscle force to propel oneself (as in jumping or sprinting), or to throw an object. | Physical Strength | 0 | 0 |
| Extent Flexibility | The ability to bend, stretch, twist, or reach with your body, arms, and/or legs. | Flexibility, balance and coordination | -3.051 | -3.051 |
| Far Vision | The ability to see details at a distance. | Visual Abilities | -2.721 | -2.721 |
| Finger Dexterity | The ability to make precisely coordinated movements of the fingers of one or both hands to grasp, manipulate, or assemble very small objects. | Fine manipulative abilities | 2.348 | 2.348 |
| Flexibility of Closure | The ability to identify or detect a known pattern (a figure, object, word, or sound) that is hidden in other distracting material. | Perecptual Abilities | 0.394 | 0.394 |
| Fluency of ideas | The ability to come up with a number of ideas about a topic (the number of ideas is important, not their quality, correctness, or creativity). | Ideas generation and reasoning abilities | -2.165 | -2.165 |
| Glare sensitivity | The ability to see objects in the presence of glare or bright lighting. | Visual abilities | -2.364 | -2.364 |
| Gross Body Co-ordination | The ability to coordinate the movement of your arms, legs, and torso together when the whole body is in motion. | Flexibility, Balance and Coordination | 0 | 0 |
| Gross Body Equilibrium | The ability to keep or regain your body balance or stay upright when in an unstable position. | Flexibility, Balance and Coordination | 0 | 0 |
| Hearing Sensitivity | The ability to detect or tell the differences between sounds that vary in pitch and loudness. | Auditory and Speech abilities | -1.262 | -1.262 |
| Inductive Reasoning | The ability to combine pieces of information to form general rules or conclusions (includes finding a relationship among seemingly unrelated events). | Idea Generation and Reasoning Abilities | 0.799 | 0.799 |
| Information Ordering | The ability to arrange things or actions in a certain order or pattern according to a specific rule or set of rules (e.g., patterns of numbers, letters, words, pictures, mathematical operations). | Idea Generation and Reasoning Abilities | -2.949 | -2.949 |
| Manual Dexterity | The ability to quickly move your hand, your hand together with your arm, or your two hands to grasp, manipulate, or assemble objects. | Fine Manipulative Abilities | -0.847 | -0.847 |
| Math Reasoning | The ability to choose the right mathematical methods or formulas to solve a problem. | Quantitative Abilities | 0.312 | 0.312 |
| Memorization | Abilities related to the recall of available information | Quantitative Abilities | -0.613 | -0.613 |
| Multi Limb Co-ordination | The ability to coordinate two or more limbs (for example, two arms, two legs, or one leg and one arm) while sitting, standing, or lying down. It does not involve performing the activities while the whole body is in motion. | Control movement abilities | 1.775 | 1.775 |
| Near Vision | The ability to see details at close range (within a few feet of the observer) | Near Vision | 0 | 0 |
| Night Vision | The ability to see under low light conditions | Near Vision | 0 | 0 |
| Number Facility | The ability to add, subtract, multiply, or divide quickly and correctly. | Quantitative Abilities | 0 | 0 |
| Oral Comprehension | The ability to listen to and understand information and ideas presented through spoken words and sentences. | Verbal Abilities | 0.94 | 0.94 |
| Oral Expression | The ability to communicate information and ideas in speaking so others will understand. | Verbal Abilities | 5.475 | 5.475 |
| Originality |  |  | -1.725 | -1.725 |
| Perceptual Speed | The ability to quickly and accurately compare similarities and differences among sets of letters, numbers, objects, pictures, or patterns. The things to be compared may be presented at the same time or one after the other. This ability also includes comparing a presented object with a remembered object. | Perceptual Abilities | -5.52 | -5.52 |
| Perceptual Vision | The ability to see objects or movement of objects to one's side when the eyes are looking ahead. | Visual Abilities | 1.025 | 1.025 |
| Problem Sensitivity | The ability to tell when something is wrong or is likely to go wrong. It does not involve solving the problem, only recognizing there is a problem. | Idea generation and Reasoning Abilities | 1.452 | 1.452 |
| Rate Control | The ability to time your movements or the movement of a piece of equipment in anticipation of changes in the speed and/or direction of a moving object or scene. | Control Movement Abilities | 2.359 | 2.359 |
| Reaction Time | The ability to quickly respond (with the hand, finger, or foot) to a signal (sound, light, picture) when it appears. | Reaction Time and Speed Abilities | 3.241 | 3.241 |
| Response Orientation | The ability to choose quickly between two or more movements in response to two or more different signals (lights, sounds, pictures). It includes the speed with which the correct response is started with the hand, foot, or other body part. | Control Movement Abilities | -4.197 | -4.197 |
| Selective Attention | The ability to concentrate on a task over a period of time without being distracted. | Attentiveness | 0 | 0 |
| Sound Localisation | The ability to tell the direction from which a sound originated. | Auditory and Speech Abilities | -0.625 | -0.625 |
| Spatial Orientation | The ability to know your location in relation to the environment or to know where other objects are in relation to you. | Spatial Abilities | 0.417 | 0.417 |
| Speech Clarity | The ability to speak clearly so others can understand you. | Auditory and Speech Abilities | 0 | 0 |
| Speech Recognition | The ability to identify and understand the speech of another person. | Auditory and Speech Abilities | -1.336 | -1.336 |
| Speed of Closure | The ability to quickly make sense of, combine, and organize information into meaningful patterns. | Perceptual Abilities | 6.411 | 6.411 |
| Speed of Limb Movement | The ability to quickly move the arms and legs. | Reaction Time and Speed Abilities | -1.055 | -1.055 |
| Stamina | The ability to exert yourself physically over long periods of time without getting winded or out of breath. | Endurance | 0 | 0 |
| Static Strength | The ability to exert maximum muscle force to lift, push, pull, or carry objects. | Physical Strength Abilities | 0.692 | 0.692 |
| Time Sharing | The ability to shift back and forth between two or more activities or sources of information (such as speech, sounds, touch, or other sources). | Attentiveness | 3.631 | 3.631 |
| Trunk Strength | The ability to use your abdominal and lower back muscles to support part of the body repeatedly or continuously over time without 'giving out' or fatiguing. | Physical Strength Abilities | 0.813 | 0.813 |
| Visual Color Discrimination | The ability to match or detect differences between colors, including shades of color and brightness. | Visual Abilities | 4.987 | 4.987 |
| Visualisation | The ability to imagine how something will look after it is moved around or when its parts are moved or rearranged. | Spatial Abilities | 0 | 0 |
| Wrist-Finger Speed | The ability to make fast, simple, repeated movements of the fingers, hands, and wrists. | Reaction Time and Speed Abilities | -1.383 | -1.383 |
| Written Comprehension | The ability to read and understand information and ideas presented in writing. | Verbal Abilities | 1.368 | 1.368 |
| Written Expression | The ability to communicate information and ideas in writing so others will understand. | Verbal Abilities | -1.285 | -1.285 |
| R Squared = 0.98 | |  | N=74745 | |

Table S2 Netherlands Skills Lasso Analysis

| ONET Item | Description |  |  | Coefficient |
| --- | --- | --- | --- | --- |
| Active Learning | Understanding the implications of new information for both current and future problem-solving and decision-making. | Process | Basic Skills | 0 |
| Active Listening | Giving full attention to what other people are saying, taking time to understand the points being made, asking questions as appropriate, and not interrupting at inappropriate times. | Content | Basic Skills | -1.513 |
| Complex Problem Solving | Identifying complex problems and reviewing related information to develop and evaluate options and implement solutions. | Complex Problem Solving | Cross Functional Skills | -2.967 |
| Coordination | Adjusting actions in relation to others' actions. | Social Skills | Cross Functional Skills | -6.469 |
| Critical Thinking | Using logic and reasoning to identify the strengths and weaknesses of alternative solutions, conclusions or approaches to problems. | Process | Basic Skills | 0 |
| Equipment Maintenance | Performing routine maintenance on equipment and determining when and what kind of maintenance is needed. | Technical Skills | Cross Functional Skills | -2.464 |
| Equipment Selection | Determining the kind of tools and equipment needed to do a job. | Technical Skills | Cross Functional Skills | 1.287 |
| Installation | Installing equipment, machines, wiring, or programs to meet specifications. | Technical Skills | Cross Functional Skills | 0 |
| Instructing | Teaching others how to do something. | Social Skills | Cross Functional Skills | -3.833 |
| Judgement and Decision Making | Considering the relative costs and benefits of potential actions to choose the most appropriate one. | Systems Skills | Cross Functional Skills | 6.711 |
| Learning Strategies | Selecting and using training/instructional methods and procedures appropriate for the situation when learning or teaching new things. | Systems Skills | Cross Functional Skills | 1.403 |
| Management of Financial Resources | Determining how money will be spent to get the work done, and accounting for these expenditures. | Resource Management Skills | Cross functional Skills | -0.462 |
| Management of Material Resources | Obtaining and seeing to the appropriate use of equipment, facilities, and materials needed to do certain work. | Resource Management Skills | Cross functional Skills | 0 |
| Management of Personell Resources | Motivating, developing, and directing people as they work, identifying the best people for the job. | Resource Management Skills | Cross functional Skills | 2.952 |
| Mathematics | Using mathematics to solve problems. | Content | Basic Skills | -0.24 |
| Monitoring | Monitoring/Assessing performance of yourself, other individuals, or organizations to make improvements or take corrective action. | Process | Basic Skills | -8.961 |
| Negotiation | Bringing others together and trying to reconcile differences. | Social Skills | Cross functional Skills | 1.889 |
| Operation Monitoring | Watching gauges, dials, or other indicators to make sure a machine is working properly. | Technical Skills | Cross Functional Skills | 3.602 |
| Operation and Control | Controlling operations of equipment or systems. | Technical Skills | Cross Functional Skills | -0.467 |
| Operations Analysis | Analyzing needs and product requirements to create a design. | Technical Skills | Cross Functional Skills | -0.601 |
| Persuasion | Persuading others to change their minds or behavior. | Social Skills | Cross functional Skills | -2.004 |
| Programming | Writing computer programs for various purposes. | Technical Skills | Cross functional Skills | -1.354 |
| Quality Control Analysis | Conducting tests and inspections of products, services, or processes to evaluate quality or performance. | Technical Skills | Cross functional Skills | -0.514 |
| Reading Comprehension | Understanding written sentences and paragraphs in work related documents. | Content | Basic Skills | 0 |
| Repairing | Repairing machines or systems using the needed tools. | Technical Skills | Cross Functional Skills | 0.112 |
| Science | Using scientific rules and methods to solve problems. | Content | Basic Skills | 0 |
| Service Orientation | Actively looking for ways to help people. | Social Skills | Cross Functional Skills | 0.955 |
| Social Perceptiveness | Being aware of others' reactions and understanding why they react as they do. | Social Skills | Cross Functional Skills | -1.569 |
| Speaking | Talking to others to convey information effectively. | Content | Basic Skills | 3.656 |
| System Analysis | Determining how a system should work and how changes in conditions, operations, and the environment will affect outcomes. | Systems Skills | Cross Functional Skills | 0 |
| System Evaluation | Identifying measures or indicators of system performance and the actions needed to improve or correct performance, relative to the goals of the system. | Systems Skills | Cross Functional Skills | 2.682 |
| Technology Design | Generating or adapting equipment and technology to serve user needs. | Technical Skills | Cross Functional Skills | -2.594 |
| Time Management | Managing one's own time and the time of others. | Resource Management Skills | Cross Functional Skills | 2.315 |
| Troubleshooting | Determining causes of operating errors and deciding what to do about it. | Technical Skills | Cross Functional Skills | 0 |
| Writing | Communicating effectively in writing as appropriate for the needs of the audience. | Content | Basic Skills | 1.473 |
| R squared = 0.75 |  |  | N= | 78745 |

Table S3 Netherlands People, Brains, Brawn Analysis

|  | Netherlands – EU LFS |
| --- | --- |
| People | -0.014*** |
|  | (0.001) |
| Brains | -0.071*** |
|  | (0.001) |
| Brawn | 0.039*** |
|  | (0.001) |
| People * Brains | -0.001*** |
|  | (0.000) |
| People*Brawn | 0.001*** |
|  | (0.000) |
| Brains*Brawn | -0.009*** |
|  | (0.000) |
| N | 74842 |
| R Squared | 12% |

***, **, * denotes significance at the 1%, 5% and 10% levels respectively

S22 Appendix. Individual country Norway

Table T1 Norway Ability Lasso Analysis

| ONET Item | Description |  |  | Coefficient |
| --- | --- | --- | --- | --- |
| Arm-Hand Steadiness | The ability to keep your hand and arm steady while moving your arm or while holding your arm and hand in one position. | Fine manipulative abilities | Psychomotor abilities | -3.267 |
| Auditory Attention | The ability to focus on a single source of sound in the presence of other distracting sounds. | Auditory and speech abilities | Sensory Abilities | 0.191 |
| Category Flexibility | The ability to generate or use different sets of rules for combining or grouping things in different ways. | Idea Generation and Reasoning Abilities | Cognitive Abilities | -0.559 |
| Control Precision | The ability to quickly and repeatedly adjust the controls of a machine or a vehicle to exact positions. | Control Movement Abilities | Psychomotor Abilities | 2.538 |
| Deductive Reasoning | The ability to apply general rules to specific problems to produce answers that make sense. | Idea Generation and Reasoning Abilities | Cognitive Abilities | -9.808 |
| Depth Perception | The ability to judge which of several objects is closer or farther away from you, or to judge the distance between you and an object. | Visual Abilities | Sensory Abilities | -0.719 |
| Dynamic Flexibility | The ability to quickly and repeatedly bend, stretch, twist, or reach out with your body, arms, and/or legs. | Flexibility, Balance and Coordination | Physical Abilities | 7.725 |
| Dynamic Strength | The ability to exert muscle force repeatedly or continuously over time. This involves muscular endurance and resistance to muscle fatigue. | Physical Strength | Physical Abilities | -3.661 |
| Explosive Strength | The ability to use short bursts of muscle force to propel oneself (as in jumping or sprinting), or to throw an object. | Physical Strength | Physical Abilities | 1.096 |
| Extent Flexibility | The ability to bend, stretch, twist, or reach with your body, arms, and/or legs. | Flexibility, balance and coordination | Physical Abilities | -1.212 |
| Far Vision | The ability to see details at a distance. | Visual Abilities | Sensory Abilities | -2.393 |
| Finger Dexterity | The ability to make precisely coordinated movements of the fingers of one or both hands to grasp, manipulate, or assemble very small objects. | Fine manipulative abilities | Psychomotor abilities | 0 |
| Flexibility of Closure | The ability to identify or detect a known pattern (a figure, object, word, or sound) that is hidden in other distracting material. | Perecptual Abilities | Cognitive abilities | 1.019 |
| Fluency of ideas | The ability to come up with a number of ideas about a topic (the number of ideas is important, not their quality, correctness, or creativity). | Ideas generation and reasoning abilities | Cognitive abilities | -6.883 |
| Glare sensitivity | The ability to see objects in the presence of glare or bright lighting. | Visual abilities | Sensory abilities | -1.185 |
| Gross Body Co-ordination | The ability to coordinate the movement of your arms, legs, and torso together when the whole body is in motion. | Flexibility, Balance and Coordination | Physical Abilities | 0 |
| Gross Body Equilibrium | The ability to keep or regain your body balance or stay upright when in an unstable position. | Flexibility, Balance and Coordination | Physical Abilities | 0 |
| Hearing Sensitivity | The ability to detect or tell the differences between sounds that vary in pitch and loudness. | Auditory and Speech abilities | Sensory Abilities | 0 |
| Inductive Reasoning | The ability to combine pieces of information to form general rules or conclusions (includes finding a relationship among seemingly unrelated events). | Idea Generation and Reasoning Abilities | Cognitive Abilities | 0 |
| Information Ordering | The ability to arrange things or actions in a certain order or pattern according to a specific rule or set of rules (e.g., patterns of numbers, letters, words, pictures, mathematical operations). | Idea Generation and Reasoning Abilities | Cognitive Abilities | 0 |
| Manual Dexterity | The ability to quickly move your hand, your hand together with your arm, or your two hands to grasp, manipulate, or assemble objects. | Fine Manipulative Abilities | Psychomotor Abilities | 0 |
| Math Reasoning | The ability to choose the right mathematical methods or formulas to solve a problem. | Quantitative Abilities | Cognitive Abilities | 1.992 |
| Memorization | Abilities related to the recall of available information | Quantitative Abilities | Cognitive Abilities | 0.747 |
| Multi Limb Co-ordination | The ability to coordinate two or more limbs (for example, two arms, two legs, or one leg and one arm) while sitting, standing, or lying down. It does not involve performing the activities while the whole body is in motion. | Control movement abilities | Psychomotor Abilities | 0.559 |
| Near Vision | The ability to see details at close range (within a few feet of the observer) | Near Vision | Visual Abilities | 0.603 |
| Night Vision | The ability to see under low light conditions | Near Vision | Visual Abilities | -1.855 |
| Number Facility | The ability to add, subtract, multiply, or divide quickly and correctly. | Quantitative Abilities | Cognitive Abilities | 0 |
| Oral Comprehension | The ability to listen to and understand information and ideas presented through spoken words and sentences. | Verbal Abilities | Cognitive Abilities | -0.953 |
| Oral Expression | The ability to communicate information and ideas in speaking so others will understand. | Verbal Abilities | Cognitive Abilities | 2.907 |
| Originality |  |  |  | 3.551 |
| Perceptual Speed | The ability to quickly and accurately compare similarities and differences among sets of letters, numbers, objects, pictures, or patterns. The things to be compared may be presented at the same time or one after the other. This ability also includes comparing a presented object with a remembered object. | Perceptual Abilities | Cognitive Abilities | -7.266 |
| Perceptual Vision | The ability to see objects or movement of objects to one's side when the eyes are looking ahead. | Visual Abilities | Sensory Abilities | 1.934 |
| Problem Sensitivity | The ability to tell when something is wrong or is likely to go wrong. It does not involve solving the problem, only recognizing there is a problem. | Idea generation and Reasoning Abilities | Cognitive Abilities | 4.762 |
| Rate Control | The ability to time your movements or the movement of a piece of equipment in anticipation of changes in the speed and/or direction of a moving object or scene. | Control Movement Abilities | Psychomotor Abilities | 2.369 |
| Reaction Time | The ability to quickly respond (with the hand, finger, or foot) to a signal (sound, light, picture) when it appears. | Reaction Time and Speed Abilities | Psychomotor Abilities | 0 |
| Response Orientation | The ability to choose quickly between two or more movements in response to two or more different signals (lights, sounds, pictures). It includes the speed with which the correct response is started with the hand, foot, or other body part. | Control Movement Abilities | Psychomotor Abilities | 0 |
| Selective Attention | The ability to concentrate on a task over a period of time without being distracted. | Attentiveness | Cognitive Abilities | 0 |
| Sound Localisation | The ability to tell the direction from which a sound originated. | Auditory and Speech Abilities | Sensory Abilities | 0 |
| Spatial Orientation | The ability to know your location in relation to the environment or to know where other objects are in relation to you. | Spatial Abilities | Cognitive Abilities | 0 |
| Speech Clarity | The ability to speak clearly so others can understand you. | Auditory and Speech Abilities | Sensory Abilities | 0.516 |
| Speech Recognition | The ability to identify and understand the speech of another person. | Auditory and Speech Abilities | Sensory Abilities | -0.739 |
| Speed of Closure | The ability to quickly make sense of, combine, and organize information into meaningful patterns. | Perceptual Abilities | Cognitive Abilities | 5.534 |
| Speed of Limb Movement | The ability to quickly move the arms and legs. | Reaction Time and Speed Abilities | Psychomotor Abilities | 0 |
| Stamina | The ability to exert yourself physically over long periods of time without getting winded or out of breath. | Endurance | Physical Abilities | 1.761 |
| Static Strength | The ability to exert maximum muscle force to lift, push, pull, or carry objects. | Physical Strength Abilities | Physical Abilities | 0 |
| Time Sharing | The ability to shift back and forth between two or more activities or sources of information (such as speech, sounds, touch, or other sources). | Attentiveness | Cognitive Abilities | 0 |
| Trunk Strength | The ability to use your abdominal and lower back muscles to support part of the body repeatedly or continuously over time without 'giving out' or fatiguing. | Physical Strength Abilities | Physical Abilities | -0.547 |
| Visual Color Discrimination | The ability to match or detect differences between colors, including shades of color and brightness. | Visual Abilities | Sensory Abilities | 3.802 |
| Visualisation | The ability to imagine how something will look after it is moved around or when its parts are moved or rearranged. | Spatial Abilities | Cognitive Abilities | -0.719 |
| Wrist-Finger Speed | The ability to make fast, simple, repeated movements of the fingers, hands, and wrists. | Reaction Time and Speed Abilities | Psychomotor Abilities | 0 |
| Written Comprehension | The ability to read and understand information and ideas presented in writing. | Verbal Abilities | Cognitive Abilities | 0.723 |
| Written Expression | The ability to communicate information and ideas in writing so others will understand. | Verbal Abilities | Cognitive Abilities | 0 |
| R Squared = 0.93 | |  | N=25624 | |

Table T2 Norway Skills Lasso Analysis

| ONET Item | Description |  |  | Coefficient |
| --- | --- | --- | --- | --- |
| Active Learning | Understanding the implications of new information for both current and future problem-solving and decision-making. | Process | Basic Skills | Auto |
| Active Listening | Giving full attention to what other people are saying, taking time to understand the points being made, asking questions as appropriate, and not interrupting at inappropriate times. | Content | Basic Skills | 0 |
| Complex Problem Solving | Identifying complex problems and reviewing related information to develop and evaluate options and implement solutions. | Complex Problem Solving | Cross Functional Skills | 0 |
| Coordination | Adjusting actions in relation to others' actions. | Social Skills | Cross Functional Skills | -0.849 |
| Critical Thinking | Using logic and reasoning to identify the strengths and weaknesses of alternative solutions, conclusions or approaches to problems. | Process | Basic Skills | -3.075 |
| Equipment Maintenance | Performing routine maintenance on equipment and determining when and what kind of maintenance is needed. | Technical Skills | Cross Functional Skills | -2.241 |
| Equipment Selection | Determining the kind of tools and equipment needed to do a job. | Technical Skills | Cross Functional Skills | -1.883 |
| Installation | Installing equipment, machines, wiring, or programs to meet specifications. | Technical Skills | Cross Functional Skills | 1.612 |
| Instructing | Teaching others how to do something. | Social Skills | Cross Functional Skills | 0.073 |
| Judgement and Decision Making | Considering the relative costs and benefits of potential actions to choose the most appropriate one. | Systems Skills | Cross Functional Skills | -2.641 |
| Learning Strategies | Selecting and using training/instructional methods and procedures appropriate for the situation when learning or teaching new things. | Systems Skills | Cross Functional Skills | 3.924 |
| Management of Financial Resources | Determining how money will be spent to get the work done, and accounting for these expenditures. | Resource Management Skills | Cross functional Skills | 1.635 |
| Management of Material Resources | Obtaining and seeing to the appropriate use of equipment, facilities, and materials needed to do certain work. | Resource Management Skills | Cross functional Skills | 0.524 |
| Management of Personell Resources | Motivating, developing, and directing people as they work, identifying the best people for the job. | Resource Management Skills | Cross functional Skills | -0.79 |
| Mathematics | Using mathematics to solve problems. | Content | Basic Skills | 0 |
| Monitoring | Monitoring/Assessing performance of yourself, other individuals, or organizations to make improvements or take corrective action. | Process | Basic Skills | 0 |
| Negotiation | Bringing others together and trying to reconcile differences. | Social Skills | Cross functional Skills | -8.077 |
| Operation Monitoring | Watching gauges, dials, or other indicators to make sure a machine is working properly. | Technical Skills | Cross Functional Skills | 2.389 |
| Operation and Control | Controlling operations of equipment or systems. | Technical Skills | Cross Functional Skills | 2.356 |
| Operations Analysis | Analyzing needs and product requirements to create a design. | Technical Skills | Cross Functional Skills | 0 |
| Persuasion | Persuading others to change their minds or behavior. | Social Skills | Cross functional Skills | -0.905 |
| Programming | Writing computer programs for various purposes. | Technical Skills | Cross functional Skills | -1.294 |
| Quality Control Analysis | Conducting tests and inspections of products, services, or processes to evaluate quality or performance. | Technical Skills | Cross functional Skills | -0.751 |
| Reading Comprehension | Understanding written sentences and paragraphs in work related documents. | Content | Basic Skills | -0.871 |
| Repairing | Repairing machines or systems using the needed tools. | Technical Skills | Cross Functional Skills | -1.907 |
| Science | Using scientific rules and methods to solve problems. | Content | Basic Skills | -1.063 |
| Service Orientation | Actively looking for ways to help people. | Social Skills | Cross Functional Skills | 0.588 |
| Social Perceptiveness | Being aware of others' reactions and understanding why they react as they do. | Social Skills | Cross Functional Skills | 0 |
| Speaking | Talking to others to convey information effectively. | Content | Basic Skills | 0 |
| System Analysis | Determining how a system should work and how changes in conditions, operations, and the environment will affect outcomes. | Systems Skills | Cross Functional Skills | 0 |
| System Evaluation | Identifying measures or indicators of system performance and the actions needed to improve or correct performance, relative to the goals of the system. | Systems Skills | Cross Functional Skills | 0.966 |
| Technology Design | Generating or adapting equipment and technology to serve user needs. | Technical Skills | Cross Functional Skills | 2.025 |
| Time Management | Managing one's own time and the time of others. | Resource Management Skills | Cross Functional Skills | -3.365 |
| Troubleshooting | Determining causes of operating errors and deciding what to do about it. | Technical Skills | Cross Functional Skills | 2.172 |
| Writing | Communicating effectively in writing as appropriate for the needs of the audience. | Content | Basic Skills | 1.649 |
| R squared = 0.78 |  |  | N= | 25624 |

Table T3 Norway People, Brains, Brawn Analysis

|  | Norway – EU LFS |
| --- | --- |
| People | -0.010*** |
|  | (0.001) |
| Brains | -0.086*** |
|  | (0.002) |
| Brawn | -0.006*** |
|  | (0.001) |
| People * Brains | 0.004*** |
|  | (0.001) |
| People*Brawn | -0.006*** |
|  | (0.001) |
| Brains*Brawn | -0.003*** |
|  | (0.001) |
| N | 25247 |
| R Squared | 12% |

***, **, * denotes significance at the 1%, 5% and 10% levels respectively

S23 Appendix. Individual country Portugal

Table U1 Portugal Ability Lasso Analysis

| ONET Item | Description |  |  | Coefficient |
| --- | --- | --- | --- | --- |
| Arm-Hand Steadiness | The ability to keep your hand and arm steady while moving your arm or while holding your arm and hand in one position. | Fine manipulative abilities | Psychomotor abilities | -4.663 |
| Auditory Attention | The ability to focus on a single source of sound in the presence of other distracting sounds. | Auditory and speech abilities | Sensory Abilities | 0 |
| Category Flexibility | The ability to generate or use different sets of rules for combining or grouping things in different ways. | Idea Generation and Reasoning Abilities | Cognitive Abilities | -1.474 |
| Control Precision | The ability to quickly and repeatedly adjust the controls of a machine or a vehicle to exact positions. | Control Movement Abilities | Psychomotor Abilities | 3.058 |
| Deductive Reasoning | The ability to apply general rules to specific problems to produce answers that make sense. | Idea Generation and Reasoning Abilities | Cognitive Abilities | -6.413 |
| Depth Perception | The ability to judge which of several objects is closer or farther away from you, or to judge the distance between you and an object. | Visual Abilities | Sensory Abilities | 0 |
| Dynamic Flexibility | The ability to quickly and repeatedly bend, stretch, twist, or reach out with your body, arms, and/or legs. | Flexibility, Balance and Coordination | Physical Abilities | 3.108 |
| Dynamic Strength | The ability to exert muscle force repeatedly or continuously over time. This involves muscular endurance and resistance to muscle fatigue. | Physical Strength | Physical Abilities | -0.954 |
| Explosive Strength | The ability to use short bursts of muscle force to propel oneself (as in jumping or sprinting), or to throw an object. | Physical Strength | Physical Abilities | 0.383 |
| Extent Flexibility | The ability to bend, stretch, twist, or reach with your body, arms, and/or legs. | Flexibility, balance and coordination | Physical Abilities | -1.87 |
| Far Vision | The ability to see details at a distance. | Visual Abilities | Sensory Abilities | -2.329 |
| Finger Dexterity | The ability to make precisely coordinated movements of the fingers of one or both hands to grasp, manipulate, or assemble very small objects. | Fine manipulative abilities | Psychomotor abilities | 1.85 |
| Flexibility of Closure | The ability to identify or detect a known pattern (a figure, object, word, or sound) that is hidden in other distracting material. | Perecptual Abilities | Cognitive abilities | 0 |
| Fluency of ideas | The ability to come up with a number of ideas about a topic (the number of ideas is important, not their quality, correctness, or creativity). | Ideas generation and reasoning abilities | Cognitive abilities | -3.597 |
| Glare sensitivity | The ability to see objects in the presence of glare or bright lighting. | Visual abilities | Sensory abilities | -2.289 |
| Gross Body Co-ordination | The ability to coordinate the movement of your arms, legs, and torso together when the whole body is in motion. | Flexibility, Balance and Coordination | Physical Abilities | 0 |
| Gross Body Equilibrium | The ability to keep or regain your body balance or stay upright when in an unstable position. | Flexibility, Balance and Coordination | Physical Abilities | 0 |
| Hearing Sensitivity | The ability to detect or tell the differences between sounds that vary in pitch and loudness. | Auditory and Speech abilities | Sensory Abilities | 0 |
| Inductive Reasoning | The ability to combine pieces of information to form general rules or conclusions (includes finding a relationship among seemingly unrelated events). | Idea Generation and Reasoning Abilities | Cognitive Abilities | 0 |
| Information Ordering | The ability to arrange things or actions in a certain order or pattern according to a specific rule or set of rules (e.g., patterns of numbers, letters, words, pictures, mathematical operations). | Idea Generation and Reasoning Abilities | Cognitive Abilities | 0 |
| Manual Dexterity | The ability to quickly move your hand, your hand together with your arm, or your two hands to grasp, manipulate, or assemble objects. | Fine Manipulative Abilities | Psychomotor Abilities | 0 |
| Math Reasoning | The ability to choose the right mathematical methods or formulas to solve a problem. | Quantitative Abilities | Cognitive Abilities | 0 |
| Memorization | Abilities related to the recall of available information | Quantitative Abilities | Cognitive Abilities | 0.65 |
| Multi Limb Co-ordination | The ability to coordinate two or more limbs (for example, two arms, two legs, or one leg and one arm) while sitting, standing, or lying down. It does not involve performing the activities while the whole body is in motion. | Control movement abilities | Psychomotor Abilities | 1.052 |
| Near Vision | The ability to see details at close range (within a few feet of the observer) | Near Vision | Visual Abilities | 0 |
| Night Vision | The ability to see under low light conditions | Near Vision | Visual Abilities | 0 |
| Number Facility | The ability to add, subtract, multiply, or divide quickly and correctly. | Quantitative Abilities | Cognitive Abilities | 0 |
| Oral Comprehension | The ability to listen to and understand information and ideas presented through spoken words and sentences. | Verbal Abilities | Cognitive Abilities | 0 |
| Oral Expression | The ability to communicate information and ideas in speaking so others will understand. | Verbal Abilities | Cognitive Abilities | 3.962 |
| Originality |  |  |  | 0 |
| Perceptual Speed | The ability to quickly and accurately compare similarities and differences among sets of letters, numbers, objects, pictures, or patterns. The things to be compared may be presented at the same time or one after the other. This ability also includes comparing a presented object with a remembered object. | Perceptual Abilities | Cognitive Abilities | -5.847 |
| Perceptual Vision | The ability to see objects or movement of objects to one's side when the eyes are looking ahead. | Visual Abilities | Sensory Abilities | 3.416 |
| Problem Sensitivity | The ability to tell when something is wrong or is likely to go wrong. It does not involve solving the problem, only recognizing there is a problem. | Idea generation and Reasoning Abilities | Cognitive Abilities | 2.731 |
| Rate Control | The ability to time your movements or the movement of a piece of equipment in anticipation of changes in the speed and/or direction of a moving object or scene. | Control Movement Abilities | Psychomotor Abilities | 1.406 |
| Reaction Time | The ability to quickly respond (with the hand, finger, or foot) to a signal (sound, light, picture) when it appears. | Reaction Time and Speed Abilities | Psychomotor Abilities | 3.2 |
| Response Orientation | The ability to choose quickly between two or more movements in response to two or more different signals (lights, sounds, pictures). It includes the speed with which the correct response is started with the hand, foot, or other body part. | Control Movement Abilities | Psychomotor Abilities | -4.826 |
| Selective Attention | The ability to concentrate on a task over a period of time without being distracted. | Attentiveness | Cognitive Abilities | -0.28 |
| Sound Localisation | The ability to tell the direction from which a sound originated. | Auditory and Speech Abilities | Sensory Abilities | -2.585 |
| Spatial Orientation | The ability to know your location in relation to the environment or to know where other objects are in relation to you. | Spatial Abilities | Cognitive Abilities | 0.881 |
| Speech Clarity | The ability to speak clearly so others can understand you. | Auditory and Speech Abilities | Sensory Abilities | 0 |
| Speech Recognition | The ability to identify and understand the speech of another person. | Auditory and Speech Abilities | Sensory Abilities | -2.608 |
| Speed of Closure | The ability to quickly make sense of, combine, and organize information into meaningful patterns. | Perceptual Abilities | Cognitive Abilities | 5.069 |
| Speed of Limb Movement | The ability to quickly move the arms and legs. | Reaction Time and Speed Abilities | Psychomotor Abilities | -1.231 |
| Stamina | The ability to exert yourself physically over long periods of time without getting winded or out of breath. | Endurance | Physical Abilities | 1.536 |
| Static Strength | The ability to exert maximum muscle force to lift, push, pull, or carry objects. | Physical Strength Abilities | Physical Abilities | 0 |
| Time Sharing | The ability to shift back and forth between two or more activities or sources of information (such as speech, sounds, touch, or other sources). | Attentiveness | Cognitive Abilities | 3.328 |
| Trunk Strength | The ability to use your abdominal and lower back muscles to support part of the body repeatedly or continuously over time without 'giving out' or fatiguing. | Physical Strength Abilities | Physical Abilities | 0 |
| Visual Color Discrimination | The ability to match or detect differences between colors, including shades of color and brightness. | Visual Abilities | Sensory Abilities | 5.319 |
| Visualisation | The ability to imagine how something will look after it is moved around or when its parts are moved or rearranged. | Spatial Abilities | Cognitive Abilities | -1.384 |
| Wrist-Finger Speed | The ability to make fast, simple, repeated movements of the fingers, hands, and wrists. | Reaction Time and Speed Abilities | Psychomotor Abilities | -0.773 |
| Written Comprehension | The ability to read and understand information and ideas presented in writing. | Verbal Abilities | Cognitive Abilities | 0.762 |
| Written Expression | The ability to communicate information and ideas in writing so others will understand. | Verbal Abilities | Cognitive Abilities | -0.237 |
| R Squared = 0.99 | |  | N=108643 | |

Table U2 Portugal Skills Lasso Analysis

| ONET Item | Description |  |  | Coefficient |
| --- | --- | --- | --- | --- |
| Active Learning | Understanding the implications of new information for both current and future problem-solving and decision-making. | Process | Basic Skills | 2.492 |
| Active Listening | Giving full attention to what other people are saying, taking time to understand the points being made, asking questions as appropriate, and not interrupting at inappropriate times. | Content | Basic Skills | 0 |
| Complex Problem Solving | Identifying complex problems and reviewing related information to develop and evaluate options and implement solutions. | Complex Problem Solving | Cross Functional Skills | -1.798 |
| Coordination | Adjusting actions in relation to others' actions. | Social Skills | Cross Functional Skills | -6.175 |
| Critical Thinking | Using logic and reasoning to identify the strengths and weaknesses of alternative solutions, conclusions or approaches to problems. | Process | Basic Skills | -4.707 |
| Equipment Maintenance | Performing routine maintenance on equipment and determining when and what kind of maintenance is needed. | Technical Skills | Cross Functional Skills | 0 |
| Equipment Selection | Determining the kind of tools and equipment needed to do a job. | Technical Skills | Cross Functional Skills | 1.659 |
| Installation | Installing equipment, machines, wiring, or programs to meet specifications. | Technical Skills | Cross Functional Skills | 0 |
| Instructing | Teaching others how to do something. | Social Skills | Cross Functional Skills | -3.77 |
| Judgement and Decision Making | Considering the relative costs and benefits of potential actions to choose the most appropriate one. | Systems Skills | Cross Functional Skills | 2.079 |
| Learning Strategies | Selecting and using training/instructional methods and procedures appropriate for the situation when learning or teaching new things. | Systems Skills | Cross Functional Skills | 0.619 |
| Management of Financial Resources | Determining how money will be spent to get the work done, and accounting for these expenditures. | Resource Management Skills | Cross functional Skills | 0 |
| Management of Material Resources | Obtaining and seeing to the appropriate use of equipment, facilities, and materials needed to do certain work. | Resource Management Skills | Cross functional Skills | -0.524 |
| Management of Personell Resources | Motivating, developing, and directing people as they work, identifying the best people for the job. | Resource Management Skills | Cross functional Skills | 0.943 |
| Mathematics | Using mathematics to solve problems. | Content | Basic Skills | -0.928 |
| Monitoring | Monitoring/Assessing performance of yourself, other individuals, or organizations to make improvements or take corrective action. | Process | Basic Skills | -6.467 |
| Negotiation | Bringing others together and trying to reconcile differences. | Social Skills | Cross functional Skills | 1.511 |
| Operation Monitoring | Watching gauges, dials, or other indicators to make sure a machine is working properly. | Technical Skills | Cross Functional Skills | 3.836 |
| Operation and Control | Controlling operations of equipment or systems. | Technical Skills | Cross Functional Skills | -1.301 |
| Operations Analysis | Analyzing needs and product requirements to create a design. | Technical Skills | Cross Functional Skills | -0.902 |
| Persuasion | Persuading others to change their minds or behavior. | Social Skills | Cross functional Skills | -1.078 |
| Programming | Writing computer programs for various purposes. | Technical Skills | Cross functional Skills | 0 |
| Quality Control Analysis | Conducting tests and inspections of products, services, or processes to evaluate quality or performance. | Technical Skills | Cross functional Skills | 0 |
| Reading Comprehension | Understanding written sentences and paragraphs in work related documents. | Content | Basic Skills | 0 |
| Repairing | Repairing machines or systems using the needed tools. | Technical Skills | Cross Functional Skills | -2.023 |
| Science | Using scientific rules and methods to solve problems. | Content | Basic Skills | 0.94 |
| Service Orientation | Actively looking for ways to help people. | Social Skills | Cross Functional Skills | 0 |
| Social Perceptiveness | Being aware of others' reactions and understanding why they react as they do. | Social Skills | Cross Functional Skills | 0 |
| Speaking | Talking to others to convey information effectively. | Content | Basic Skills | 5.152 |
| System Analysis | Determining how a system should work and how changes in conditions, operations, and the environment will affect outcomes. | Systems Skills | Cross Functional Skills | -2.078 |
| System Evaluation | Identifying measures or indicators of system performance and the actions needed to improve or correct performance, relative to the goals of the system. | Systems Skills | Cross Functional Skills | 6.524 |
| Technology Design | Generating or adapting equipment and technology to serve user needs. | Technical Skills | Cross Functional Skills | -3.691 |
| Time Management | Managing one's own time and the time of others. | Resource Management Skills | Cross Functional Skills | 3.527 |
| Troubleshooting | Determining causes of operating errors and deciding what to do about it. | Technical Skills | Cross Functional Skills | 0 |
| Writing | Communicating effectively in writing as appropriate for the needs of the audience. | Content | Basic Skills | -1.226 |
| R squared = 0.88 |  |  | N= | 108643 |

Table U3 Portugal People, Brains, Brawn Analysis

|  | Portugal – EU LFS |
| --- | --- |
| People | 0.006*** |
|  | (0.001) |
| Brains | -0.107*** |
|  | (0.001) |
| Brawn | 0.042*** |
|  | (0.001) |
| People * Brains | -0.028*** |
|  | (0.000) |
| People*Brawn | 0.001*** |
|  | (0.000) |
| Brains*Brawn | -0.030*** |
|  | (0.000) |
| N | 108567 |
| R Squared | 16% |

***, **, * denotes significance at the 1%, 5% and 10% levels respectively

S24 Appendix. Individual country Sweden

Table V1 Sweden Ability Lasso Analysis

| ONET Item | Description |  |  | Coefficient |
| --- | --- | --- | --- | --- |
| Arm-Hand Steadiness | The ability to keep your hand and arm steady while moving your arm or while holding your arm and hand in one position. | Fine manipulative abilities | -4.574 | -4.574 |
| Auditory Attention | The ability to focus on a single source of sound in the presence of other distracting sounds. | Auditory and speech abilities | 0.832 | 0.832 |
| Category Flexibility | The ability to generate or use different sets of rules for combining or grouping things in different ways. | Idea Generation and Reasoning Abilities | -2.515 | -2.515 |
| Control Precision | The ability to quickly and repeatedly adjust the controls of a machine or a vehicle to exact positions. | Control Movement Abilities | 2.133 | 2.133 |
| Deductive Reasoning | The ability to apply general rules to specific problems to produce answers that make sense. | Idea Generation and Reasoning Abilities | -6.99 | -6.99 |
| Depth Perception | The ability to judge which of several objects is closer or farther away from you, or to judge the distance between you and an object. | Visual Abilities | 0 | 0 |
| Dynamic Flexibility | The ability to quickly and repeatedly bend, stretch, twist, or reach out with your body, arms, and/or legs. | Flexibility, Balance and Coordination | 4 | 4 |
| Dynamic Strength | The ability to exert muscle force repeatedly or continuously over time. This involves muscular endurance and resistance to muscle fatigue. | Physical Strength | -2.253 | -2.253 |
| Explosive Strength | The ability to use short bursts of muscle force to propel oneself (as in jumping or sprinting), or to throw an object. | Physical Strength | 0.654 | 0.654 |
| Extent Flexibility | The ability to bend, stretch, twist, or reach with your body, arms, and/or legs. | Flexibility, balance and coordination | -2.58 | -2.58 |
| Far Vision | The ability to see details at a distance. | Visual Abilities | -3.289 | -3.289 |
| Finger Dexterity | The ability to make precisely coordinated movements of the fingers of one or both hands to grasp, manipulate, or assemble very small objects. | Fine manipulative abilities | 3.215 | 3.215 |
| Flexibility of Closure | The ability to identify or detect a known pattern (a figure, object, word, or sound) that is hidden in other distracting material. | Perecptual Abilities | 0 | 0 |
| Fluency of ideas | The ability to come up with a number of ideas about a topic (the number of ideas is important, not their quality, correctness, or creativity). | Ideas generation and reasoning abilities | -3.908 | -3.908 |
| Glare sensitivity | The ability to see objects in the presence of glare or bright lighting. | Visual abilities | -3.234 | -3.234 |
| Gross Body Co-ordination | The ability to coordinate the movement of your arms, legs, and torso together when the whole body is in motion. | Flexibility, Balance and Coordination | 0 | 0 |
| Gross Body Equilibrium | The ability to keep or regain your body balance or stay upright when in an unstable position. | Flexibility, Balance and Coordination | 0 | 0 |
| Hearing Sensitivity | The ability to detect or tell the differences between sounds that vary in pitch and loudness. | Auditory and Speech abilities | -1.882 | -1.882 |
| Inductive Reasoning | The ability to combine pieces of information to form general rules or conclusions (includes finding a relationship among seemingly unrelated events). | Idea Generation and Reasoning Abilities | 0 | 0 |
| Information Ordering | The ability to arrange things or actions in a certain order or pattern according to a specific rule or set of rules (e.g., patterns of numbers, letters, words, pictures, mathematical operations). | Idea Generation and Reasoning Abilities | 0 | 0 |
| Manual Dexterity | The ability to quickly move your hand, your hand together with your arm, or your two hands to grasp, manipulate, or assemble objects. | Fine Manipulative Abilities | 0 | 0 |
| Math Reasoning | The ability to choose the right mathematical methods or formulas to solve a problem. | Quantitative Abilities | 0.318 | 0.318 |
| Memorization | Abilities related to the recall of available information | Quantitative Abilities | 0 | 0 |
| Multi Limb Co-ordination | The ability to coordinate two or more limbs (for example, two arms, two legs, or one leg and one arm) while sitting, standing, or lying down. It does not involve performing the activities while the whole body is in motion. | Control movement abilities | 1.897 | 1.897 |
| Near Vision | The ability to see details at close range (within a few feet of the observer) | Near Vision | 0 | 0 |
| Night Vision | The ability to see under low light conditions | Near Vision | 0 | 0 |
| Number Facility | The ability to add, subtract, multiply, or divide quickly and correctly. | Quantitative Abilities | 0 | 0 |
| Oral Comprehension | The ability to listen to and understand information and ideas presented through spoken words and sentences. | Verbal Abilities | 0 | 0 |
| Oral Expression | The ability to communicate information and ideas in speaking so others will understand. | Verbal Abilities | 3.548 | 3.548 |
| Originality |  |  | 0 | 0 |
| Perceptual Speed | The ability to quickly and accurately compare similarities and differences among sets of letters, numbers, objects, pictures, or patterns. The things to be compared may be presented at the same time or one after the other. This ability also includes comparing a presented object with a remembered object. | Perceptual Abilities | -5.404 | -5.404 |
| Perceptual Vision | The ability to see objects or movement of objects to one's side when the eyes are looking ahead. | Visual Abilities | 2.771 | 2.771 |
| Problem Sensitivity | The ability to tell when something is wrong or is likely to go wrong. It does not involve solving the problem, only recognizing there is a problem. | Idea generation and Reasoning Abilities | 3.361 | 3.361 |
| Rate Control | The ability to time your movements or the movement of a piece of equipment in anticipation of changes in the speed and/or direction of a moving object or scene. | Control Movement Abilities | 2.833 | 2.833 |
| Reaction Time | The ability to quickly respond (with the hand, finger, or foot) to a signal (sound, light, picture) when it appears. | Reaction Time and Speed Abilities | 2.024 | 2.024 |
| Response Orientation | The ability to choose quickly between two or more movements in response to two or more different signals (lights, sounds, pictures). It includes the speed with which the correct response is started with the hand, foot, or other body part. | Control Movement Abilities | -4.621 | -4.621 |
| Selective Attention | The ability to concentrate on a task over a period of time without being distracted. | Attentiveness | -1.029 | -1.029 |
| Sound Localisation | The ability to tell the direction from which a sound originated. | Auditory and Speech Abilities | -1.048 | -1.048 |
| Spatial Orientation | The ability to know your location in relation to the environment or to know where other objects are in relation to you. | Spatial Abilities | 1.188 | 1.188 |
| Speech Clarity | The ability to speak clearly so others can understand you. | Auditory and Speech Abilities | 0.865 | 0.865 |
| Speech Recognition | The ability to identify and understand the speech of another person. | Auditory and Speech Abilities | -1.968 | -1.968 |
| Speed of Closure | The ability to quickly make sense of, combine, and organize information into meaningful patterns. | Perceptual Abilities | 5.997 | 5.997 |
| Speed of Limb Movement | The ability to quickly move the arms and legs. | Reaction Time and Speed Abilities | 0 | 0 |
| Stamina | The ability to exert yourself physically over long periods of time without getting winded or out of breath. | Endurance | 1.279 | 1.279 |
| Static Strength | The ability to exert maximum muscle force to lift, push, pull, or carry objects. | Physical Strength Abilities | 0.649 | 0.649 |
| Time Sharing | The ability to shift back and forth between two or more activities or sources of information (such as speech, sounds, touch, or other sources). | Attentiveness | 2.238 | 2.238 |
| Trunk Strength | The ability to use your abdominal and lower back muscles to support part of the body repeatedly or continuously over time without 'giving out' or fatiguing. | Physical Strength Abilities | 0.367 | 0.367 |
| Visual Color Discrimination | The ability to match or detect differences between colors, including shades of color and brightness. | Visual Abilities | 5.053 | 5.053 |
| Visualisation | The ability to imagine how something will look after it is moved around or when its parts are moved or rearranged. | Spatial Abilities | -0.515 | -0.515 |
| Wrist-Finger Speed | The ability to make fast, simple, repeated movements of the fingers, hands, and wrists. | Reaction Time and Speed Abilities | -1.032 | -1.032 |
| Written Comprehension | The ability to read and understand information and ideas presented in writing. | Verbal Abilities | 1.139 | 1.139 |
| Written Expression | The ability to communicate information and ideas in writing so others will understand. | Verbal Abilities | 0 | 0 |
| R Squared = 0.98 | |  | N=282220 | |

Table V2 Sweden Skills Lasso Analysis

| ONET Item | Description |  |  | Coefficient |
| --- | --- | --- | --- | --- |
| Active Learning | Understanding the implications of new information for both current and future problem-solving and decision-making. | Process | Basic Skills | 0 |
| Active Listening | Giving full attention to what other people are saying, taking time to understand the points being made, asking questions as appropriate, and not interrupting at inappropriate times. | Content | Basic Skills | -1.817 |
| Complex Problem Solving | Identifying complex problems and reviewing related information to develop and evaluate options and implement solutions. | Complex Problem Solving | Cross Functional Skills | -1.359 |
| Coordination | Adjusting actions in relation to others' actions. | Social Skills | Cross Functional Skills | -5.734 |
| Critical Thinking | Using logic and reasoning to identify the strengths and weaknesses of alternative solutions, conclusions or approaches to problems. | Process | Basic Skills | -2.063 |
| Equipment Maintenance | Performing routine maintenance on equipment and determining when and what kind of maintenance is needed. | Technical Skills | Cross Functional Skills | 0 |
| Equipment Selection | Determining the kind of tools and equipment needed to do a job. | Technical Skills | Cross Functional Skills | 2.507 |
| Installation | Installing equipment, machines, wiring, or programs to meet specifications. | Technical Skills | Cross Functional Skills | 0.253 |
| Instructing | Teaching others how to do something. | Social Skills | Cross Functional Skills | -3.45 |
| Judgement and Decision Making | Considering the relative costs and benefits of potential actions to choose the most appropriate one. | Systems Skills | Cross Functional Skills | 3.773 |
| Learning Strategies | Selecting and using training/instructional methods and procedures appropriate for the situation when learning or teaching new things. | Systems Skills | Cross Functional Skills | 1.669 |
| Management of Financial Resources | Determining how money will be spent to get the work done, and accounting for these expenditures. | Resource Management Skills | Cross functional Skills | -0.469 |
| Management of Material Resources | Obtaining and seeing to the appropriate use of equipment, facilities, and materials needed to do certain work. | Resource Management Skills | Cross functional Skills | 0 |
| Management of Personell Resources | Motivating, developing, and directing people as they work, identifying the best people for the job. | Resource Management Skills | Cross functional Skills | 0.299 |
| Mathematics | Using mathematics to solve problems. | Content | Basic Skills | -0.766 |
| Monitoring | Monitoring/Assessing performance of yourself, other individuals, or organizations to make improvements or take corrective action. | Process | Basic Skills | -10.242 |
| Negotiation | Bringing others together and trying to reconcile differences. | Social Skills | Cross functional Skills | 3.187 |
| Operation Monitoring | Watching gauges, dials, or other indicators to make sure a machine is working properly. | Technical Skills | Cross Functional Skills | 4.413 |
| Operation and Control | Controlling operations of equipment or systems. | Technical Skills | Cross Functional Skills | -1.546 |
| Operations Analysis | Analyzing needs and product requirements to create a design. | Technical Skills | Cross Functional Skills | -0.225 |
| Persuasion | Persuading others to change their minds or behavior. | Social Skills | Cross functional Skills | -2.033 |
| Programming | Writing computer programs for various purposes. | Technical Skills | Cross functional Skills | -1.131 |
| Quality Control Analysis | Conducting tests and inspections of products, services, or processes to evaluate quality or performance. | Technical Skills | Cross functional Skills | 0 |
| Reading Comprehension | Understanding written sentences and paragraphs in work related documents. | Content | Basic Skills | 0 |
| Repairing | Repairing machines or systems using the needed tools. | Technical Skills | Cross Functional Skills | -3.345 |
| Science | Using scientific rules and methods to solve problems. | Content | Basic Skills | 0.253 |
| Service Orientation | Actively looking for ways to help people. | Social Skills | Cross Functional Skills | 0.725 |
| Social Perceptiveness | Being aware of others' reactions and understanding why they react as they do. | Social Skills | Cross Functional Skills | 0 |
| Speaking | Talking to others to convey information effectively. | Content | Basic Skills | 4.215 |
| System Analysis | Determining how a system should work and how changes in conditions, operations, and the environment will affect outcomes. | Systems Skills | Cross Functional Skills | 0 |
| System Evaluation | Identifying measures or indicators of system performance and the actions needed to improve or correct performance, relative to the goals of the system. | Systems Skills | Cross Functional Skills | 4.553 |
| Technology Design | Generating or adapting equipment and technology to serve user needs. | Technical Skills | Cross Functional Skills | -4.3 |
| Time Management | Managing one's own time and the time of others. | Resource Management Skills | Cross Functional Skills | 2.211 |
| Troubleshooting | Determining causes of operating errors and deciding what to do about it. | Technical Skills | Cross Functional Skills | 0.918 |
| Writing | Communicating effectively in writing as appropriate for the needs of the audience. | Content | Basic Skills | 2.032 |
| R squared = 0.82 |  |  | N= | 282220 |

Table V3 Sweden People, Brains, Brawn Analysis

|  | Sweden – EU LFS |
| --- | --- |
| People | -0.017*** |
|  | (0.000) |
| Brains | -0.113*** |
|  | (0.001) |
| Brawn | 0.014*** |
|  | (0.000) |
| People * Brains | 0.018*** |
|  | (0.000) |
| People*Brawn | -0.001*** |
|  | (0.000) |
| Brains*Brawn | -0.019*** |
|  | (0.000) |
| N | 281782 |
| R Squared | 17% |

***, **, * denotes significance at the 1%, 5% and 10% levels respectively

S25 Appendix. Individual country Slovakia

Table W1 Slovakia Ability Lasso Analysis

| ONET Item | Description |  |  | Coefficient |
| --- | --- | --- | --- | --- |
| Arm-Hand Steadiness | The ability to keep your hand and arm steady while moving your arm or while holding your arm and hand in one position. | Fine manipulative abilities | Psychomotor abilities | -3.847 |
| Auditory Attention | The ability to focus on a single source of sound in the presence of other distracting sounds. | Auditory and speech abilities | Sensory Abilities | 0 |
| Category Flexibility | The ability to generate or use different sets of rules for combining or grouping things in different ways. | Idea Generation and Reasoning Abilities | Cognitive Abilities | -0.904 |
| Control Precision | The ability to quickly and repeatedly adjust the controls of a machine or a vehicle to exact positions. | Control Movement Abilities | Psychomotor Abilities | 1.338 |
| Deductive Reasoning | The ability to apply general rules to specific problems to produce answers that make sense. | Idea Generation and Reasoning Abilities | Cognitive Abilities | -7.165 |
| Depth Perception | The ability to judge which of several objects is closer or farther away from you, or to judge the distance between you and an object. | Visual Abilities | Sensory Abilities | 0 |
| Dynamic Flexibility | The ability to quickly and repeatedly bend, stretch, twist, or reach out with your body, arms, and/or legs. | Flexibility, Balance and Coordination | Physical Abilities | 3.091 |
| Dynamic Strength | The ability to exert muscle force repeatedly or continuously over time. This involves muscular endurance and resistance to muscle fatigue. | Physical Strength | Physical Abilities | -1.343 |
| Explosive Strength | The ability to use short bursts of muscle force to propel oneself (as in jumping or sprinting), or to throw an object. | Physical Strength | Physical Abilities | 1.284 |
| Extent Flexibility | The ability to bend, stretch, twist, or reach with your body, arms, and/or legs. | Flexibility, balance and coordination | Physical Abilities | -2.69 |
| Far Vision | The ability to see details at a distance. | Visual Abilities | Sensory Abilities | -2.691 |
| Finger Dexterity | The ability to make precisely coordinated movements of the fingers of one or both hands to grasp, manipulate, or assemble very small objects. | Fine manipulative abilities | Psychomotor abilities | 3.525 |
| Flexibility of Closure | The ability to identify or detect a known pattern (a figure, object, word, or sound) that is hidden in other distracting material. | Perecptual Abilities | Cognitive abilities | -0.052 |
| Fluency of ideas | The ability to come up with a number of ideas about a topic (the number of ideas is important, not their quality, correctness, or creativity). | Ideas generation and reasoning abilities | Cognitive abilities | -2.92 |
| Glare sensitivity | The ability to see objects in the presence of glare or bright lighting. | Visual abilities | Sensory abilities | -2.412 |
| Gross Body Co-ordination | The ability to coordinate the movement of your arms, legs, and torso together when the whole body is in motion. | Flexibility, Balance and Coordination | Physical Abilities | 0 |
| Gross Body Equilibrium | The ability to keep or regain your body balance or stay upright when in an unstable position. | Flexibility, Balance and Coordination | Physical Abilities | 0 |
| Hearing Sensitivity | The ability to detect or tell the differences between sounds that vary in pitch and loudness. | Auditory and Speech abilities | Sensory Abilities | -1.299 |
| Inductive Reasoning | The ability to combine pieces of information to form general rules or conclusions (includes finding a relationship among seemingly unrelated events). | Idea Generation and Reasoning Abilities | Cognitive Abilities | 0 |
| Information Ordering | The ability to arrange things or actions in a certain order or pattern according to a specific rule or set of rules (e.g., patterns of numbers, letters, words, pictures, mathematical operations). | Idea Generation and Reasoning Abilities | Cognitive Abilities | -1.637 |
| Manual Dexterity | The ability to quickly move your hand, your hand together with your arm, or your two hands to grasp, manipulate, or assemble objects. | Fine Manipulative Abilities | Psychomotor Abilities | 0 |
| Math Reasoning | The ability to choose the right mathematical methods or formulas to solve a problem. | Quantitative Abilities | Cognitive Abilities | 2.084 |
| Memorization | Abilities related to the recall of available information | Quantitative Abilities | Cognitive Abilities | 0 |
| Multi Limb Co-ordination | The ability to coordinate two or more limbs (for example, two arms, two legs, or one leg and one arm) while sitting, standing, or lying down. It does not involve performing the activities while the whole body is in motion. | Control movement abilities | Psychomotor Abilities | 1.475 |
| Near Vision | The ability to see details at close range (within a few feet of the observer) | Near Vision | Visual Abilities | -0.562 |
| Night Vision | The ability to see under low light conditions | Near Vision | Visual Abilities | 0 |
| Number Facility | The ability to add, subtract, multiply, or divide quickly and correctly. | Quantitative Abilities | Cognitive Abilities | -1.982 |
| Oral Comprehension | The ability to listen to and understand information and ideas presented through spoken words and sentences. | Verbal Abilities | Cognitive Abilities | 0 |
| Oral Expression | The ability to communicate information and ideas in speaking so others will understand. | Verbal Abilities | Cognitive Abilities | 3.214 |
| Originality |  |  |  | 0 |
| Perceptual Speed | The ability to quickly and accurately compare similarities and differences among sets of letters, numbers, objects, pictures, or patterns. The things to be compared may be presented at the same time or one after the other. This ability also includes comparing a presented object with a remembered object. | Perceptual Abilities | Cognitive Abilities | -4.478 |
| Perceptual Vision | The ability to see objects or movement of objects to one's side when the eyes are looking ahead. | Visual Abilities | Sensory Abilities | 0.74 |
| Problem Sensitivity | The ability to tell when something is wrong or is likely to go wrong. It does not involve solving the problem, only recognizing there is a problem. | Idea generation and Reasoning Abilities | Cognitive Abilities | 3.432 |
| Rate Control | The ability to time your movements or the movement of a piece of equipment in anticipation of changes in the speed and/or direction of a moving object or scene. | Control Movement Abilities | Psychomotor Abilities | 2.903 |
| Reaction Time | The ability to quickly respond (with the hand, finger, or foot) to a signal (sound, light, picture) when it appears. | Reaction Time and Speed Abilities | Psychomotor Abilities | 0 |
| Response Orientation | The ability to choose quickly between two or more movements in response to two or more different signals (lights, sounds, pictures). It includes the speed with which the correct response is started with the hand, foot, or other body part. | Control Movement Abilities | Psychomotor Abilities | -2.404 |
| Selective Attention | The ability to concentrate on a task over a period of time without being distracted. | Attentiveness | Cognitive Abilities | 0 |
| Sound Localisation | The ability to tell the direction from which a sound originated. | Auditory and Speech Abilities | Sensory Abilities | -0.307 |
| Spatial Orientation | The ability to know your location in relation to the environment or to know where other objects are in relation to you. | Spatial Abilities | Cognitive Abilities | 1.682 |
| Speech Clarity | The ability to speak clearly so others can understand you. | Auditory and Speech Abilities | Sensory Abilities | 1.371 |
| Speech Recognition | The ability to identify and understand the speech of another person. | Auditory and Speech Abilities | Sensory Abilities | -2.602 |
| Speed of Closure | The ability to quickly make sense of, combine, and organize information into meaningful patterns. | Perceptual Abilities | Cognitive Abilities | 5.444 |
| Speed of Limb Movement | The ability to quickly move the arms and legs. | Reaction Time and Speed Abilities | Psychomotor Abilities | -0.419 |
| Stamina | The ability to exert yourself physically over long periods of time without getting winded or out of breath. | Endurance | Physical Abilities | 1.249 |
| Static Strength | The ability to exert maximum muscle force to lift, push, pull, or carry objects. | Physical Strength Abilities | Physical Abilities | 0 |
| Time Sharing | The ability to shift back and forth between two or more activities or sources of information (such as speech, sounds, touch, or other sources). | Attentiveness | Cognitive Abilities | 2.685 |
| Trunk Strength | The ability to use your abdominal and lower back muscles to support part of the body repeatedly or continuously over time without 'giving out' or fatiguing. | Physical Strength Abilities | Physical Abilities | 0 |
| Visual Color Discrimination | The ability to match or detect differences between colors, including shades of color and brightness. | Visual Abilities | Sensory Abilities | 4.027 |
| Visualisation | The ability to imagine how something will look after it is moved around or when its parts are moved or rearranged. | Spatial Abilities | Cognitive Abilities | -0.526 |
| Wrist-Finger Speed | The ability to make fast, simple, repeated movements of the fingers, hands, and wrists. | Reaction Time and Speed Abilities | Psychomotor Abilities | 0 |
| Written Comprehension | The ability to read and understand information and ideas presented in writing. | Verbal Abilities | Cognitive Abilities | 0.543 |
| Written Expression | The ability to communicate information and ideas in writing so others will understand. | Verbal Abilities | Cognitive Abilities | -0.852 |
| R Squared = 0.98 | |  | N=66551 | |

Table W2 Slovakia Skills Lasso Analysis

| ONET Item | Description |  |  | Coefficient |
| --- | --- | --- | --- | --- |
| Active Learning | Understanding the implications of new information for both current and future problem-solving and decision-making. | Process | Basic Skills | 0 |
| Active Listening | Giving full attention to what other people are saying, taking time to understand the points being made, asking questions as appropriate, and not interrupting at inappropriate times. | Content | Basic Skills | -1.927 |
| Complex Problem Solving | Identifying complex problems and reviewing related information to develop and evaluate options and implement solutions. | Complex Problem Solving | Cross Functional Skills | -3.572 |
| Coordination | Adjusting actions in relation to others' actions. | Social Skills | Cross Functional Skills | -4.154 |
| Critical Thinking | Using logic and reasoning to identify the strengths and weaknesses of alternative solutions, conclusions or approaches to problems. | Process | Basic Skills | 0 |
| Equipment Maintenance | Performing routine maintenance on equipment and determining when and what kind of maintenance is needed. | Technical Skills | Cross Functional Skills | 0 |
| Equipment Selection | Determining the kind of tools and equipment needed to do a job. | Technical Skills | Cross Functional Skills | 2.262 |
| Installation | Installing equipment, machines, wiring, or programs to meet specifications. | Technical Skills | Cross Functional Skills | 0.171 |
| Instructing | Teaching others how to do something. | Social Skills | Cross Functional Skills | -2.917 |
| Judgement and Decision Making | Considering the relative costs and benefits of potential actions to choose the most appropriate one. | Systems Skills | Cross Functional Skills | 4.844 |
| Learning Strategies | Selecting and using training/instructional methods and procedures appropriate for the situation when learning or teaching new things. | Systems Skills | Cross Functional Skills | 1.611 |
| Management of Financial Resources | Determining how money will be spent to get the work done, and accounting for these expenditures. | Resource Management Skills | Cross functional Skills | 0 |
| Management of Material Resources | Obtaining and seeing to the appropriate use of equipment, facilities, and materials needed to do certain work. | Resource Management Skills | Cross functional Skills | -0.463 |
| Management of Personell Resources | Motivating, developing, and directing people as they work, identifying the best people for the job. | Resource Management Skills | Cross functional Skills | 0 |
| Mathematics | Using mathematics to solve problems. | Content | Basic Skills | -0.219 |
| Monitoring | Monitoring/Assessing performance of yourself, other individuals, or organizations to make improvements or take corrective action. | Process | Basic Skills | -8.341 |
| Negotiation | Bringing others together and trying to reconcile differences. | Social Skills | Cross functional Skills | 1.003 |
| Operation Monitoring | Watching gauges, dials, or other indicators to make sure a machine is working properly. | Technical Skills | Cross Functional Skills | 4.521 |
| Operation and Control | Controlling operations of equipment or systems. | Technical Skills | Cross Functional Skills | -1.103 |
| Operations Analysis | Analyzing needs and product requirements to create a design. | Technical Skills | Cross Functional Skills | -0.812 |
| Persuasion | Persuading others to change their minds or behavior. | Social Skills | Cross functional Skills | -0.888 |
| Programming | Writing computer programs for various purposes. | Technical Skills | Cross functional Skills | 0 |
| Quality Control Analysis | Conducting tests and inspections of products, services, or processes to evaluate quality or performance. | Technical Skills | Cross functional Skills | 0 |
| Reading Comprehension | Understanding written sentences and paragraphs in work related documents. | Content | Basic Skills | 0 |
| Repairing | Repairing machines or systems using the needed tools. | Technical Skills | Cross Functional Skills | -2.096 |
| Science | Using scientific rules and methods to solve problems. | Content | Basic Skills | -0.057 |
| Service Orientation | Actively looking for ways to help people. | Social Skills | Cross Functional Skills | 2.44 |
| Social Perceptiveness | Being aware of others' reactions and understanding why they react as they do. | Social Skills | Cross Functional Skills | -2.539 |
| Speaking | Talking to others to convey information effectively. | Content | Basic Skills | 5.083 |
| System Analysis | Determining how a system should work and how changes in conditions, operations, and the environment will affect outcomes. | Systems Skills | Cross Functional Skills | 0 |
| System Evaluation | Identifying measures or indicators of system performance and the actions needed to improve or correct performance, relative to the goals of the system. | Systems Skills | Cross Functional Skills | 3.692 |
| Technology Design | Generating or adapting equipment and technology to serve user needs. | Technical Skills | Cross Functional Skills | -4.003 |
| Time Management | Managing one's own time and the time of others. | Resource Management Skills | Cross Functional Skills | 3.849 |
| Troubleshooting | Determining causes of operating errors and deciding what to do about it. | Technical Skills | Cross Functional Skills | -1.045 |
| Writing | Communicating effectively in writing as appropriate for the needs of the audience. | Content | Basic Skills | 0 |
| R squared = 0.88 |  |  | N= | 66551 |

Table W3 Slovakia People, Brains, Brawn Analysis

|  | Slovakia – EU LFS |
| --- | --- |
| People | 0.092*** |
|  | (0.001) |
| Brains | -0.134*** |
|  | (0.001) |
| Brawn | 0.049*** |
|  | (0.001) |
| People * Brains | -0.026*** |
|  | (0.000) |
| People*Brawn | -0.013*** |
|  | (0.000) |
| Brains*Brawn | -0.029*** |
|  | (0.001) |
| N | 66509 |
| R Squared | 37% |

***, **, * denotes significance at the 1%, 5% and 10% levels respectively

S26 Appendix. Individual country UK

Table X.1 UK Ability Lasso Analysis

| ONET Item | Description |  |  | Coefficient |
| --- | --- | --- | --- | --- |
| Arm-Hand Steadiness | The ability to keep your hand and arm steady while moving your arm or while holding your arm and hand in one position. | Fine manipulative abilities | -3.019 | -3.019 |
| Auditory Attention | The ability to focus on a single source of sound in the presence of other distracting sounds. | Auditory and speech abilities | 0 | 0 |
| Category Flexibility | The ability to generate or use different sets of rules for combining or grouping things in different ways. | Idea Generation and Reasoning Abilities | -3.02 | -3.02 |
| Control Precision | The ability to quickly and repeatedly adjust the controls of a machine or a vehicle to exact positions. | Control Movement Abilities | 0.503 | 0.503 |
| Deductive Reasoning | The ability to apply general rules to specific problems to produce answers that make sense. | Idea Generation and Reasoning Abilities | -3.313 | -3.313 |
| Depth Perception | The ability to judge which of several objects is closer or farther away from you, or to judge the distance between you and an object. | Visual Abilities | 0 | 0 |
| Dynamic Flexibility | The ability to quickly and repeatedly bend, stretch, twist, or reach out with your body, arms, and/or legs. | Flexibility, Balance and Coordination | 3.213 | 3.213 |
| Dynamic Strength | The ability to exert muscle force repeatedly or continuously over time. This involves muscular endurance and resistance to muscle fatigue. | Physical Strength | 0 | 0 |
| Explosive Strength | The ability to use short bursts of muscle force to propel oneself (as in jumping or sprinting), or to throw an object. | Physical Strength | 0.9 | 0.9 |
| Extent Flexibility | The ability to bend, stretch, twist, or reach with your body, arms, and/or legs. | Flexibility, balance and coordination | -2.451 | -2.451 |
| Far Vision | The ability to see details at a distance. | Visual Abilities | 0 | 0 |
| Finger Dexterity | The ability to make precisely coordinated movements of the fingers of one or both hands to grasp, manipulate, or assemble very small objects. | Fine manipulative abilities | 1.78 | 1.78 |
| Flexibility of Closure | The ability to identify or detect a known pattern (a figure, object, word, or sound) that is hidden in other distracting material. | Perecptual Abilities | -0.888 | -0.888 |
| Fluency of ideas | The ability to come up with a number of ideas about a topic (the number of ideas is important, not their quality, correctness, or creativity). | Ideas generation and reasoning abilities | -2.243 | -2.243 |
| Glare sensitivity | The ability to see objects in the presence of glare or bright lighting. | Visual abilities | -2.756 | -2.756 |
| Gross Body Co-ordination | The ability to coordinate the movement of your arms, legs, and torso together when the whole body is in motion. | Flexibility, Balance and Coordination | 0 | 0 |
| Gross Body Equilibrium | The ability to keep or regain your body balance or stay upright when in an unstable position. | Flexibility, Balance and Coordination | -0.688 | -0.688 |
| Hearing Sensitivity | The ability to detect or tell the differences between sounds that vary in pitch and loudness. | Auditory and Speech abilities | -1.301 | -1.301 |
| Inductive Reasoning | The ability to combine pieces of information to form general rules or conclusions (includes finding a relationship among seemingly unrelated events). | Idea Generation and Reasoning Abilities | 0 | 0 |
| Information Ordering | The ability to arrange things or actions in a certain order or pattern according to a specific rule or set of rules (e.g., patterns of numbers, letters, words, pictures, mathematical operations). | Idea Generation and Reasoning Abilities | 0 | 0 |
| Manual Dexterity | The ability to quickly move your hand, your hand together with your arm, or your two hands to grasp, manipulate, or assemble objects. | Fine Manipulative Abilities | 0 | 0 |
| Math Reasoning | The ability to choose the right mathematical methods or formulas to solve a problem. | Quantitative Abilities | 1.891 | 1.891 |
| Memorization | Abilities related to the recall of available information | Quantitative Abilities | 0.897 | 0.897 |
| Multi Limb Co-ordination | The ability to coordinate two or more limbs (for example, two arms, two legs, or one leg and one arm) while sitting, standing, or lying down. It does not involve performing the activities while the whole body is in motion. | Control movement abilities | 2.378 | 2.378 |
| Near Vision | The ability to see details at close range (within a few feet of the observer) | Near Vision | 1.043 | 1.043 |
| Night Vision | The ability to see under low light conditions | Near Vision | 2.809 | 2.809 |
| Number Facility | The ability to add, subtract, multiply, or divide quickly and correctly. | Quantitative Abilities | -2.138 | -2.138 |
| Oral Comprehension | The ability to listen to and understand information and ideas presented through spoken words and sentences. | Verbal Abilities | -0.798 | -0.798 |
| Oral Expression | The ability to communicate information and ideas in speaking so others will understand. | Verbal Abilities | 3.675 | 3.675 |
| Originality |  |  | 0 | 0 |
| Perceptual Speed | The ability to quickly and accurately compare similarities and differences among sets of letters, numbers, objects, pictures, or patterns. The things to be compared may be presented at the same time or one after the other. This ability also includes comparing a presented object with a remembered object. | Perceptual Abilities | -4.412 | -4.412 |
| Perceptual Vision | The ability to see objects or movement of objects to one's side when the eyes are looking ahead. | Visual Abilities | 0 | 0 |
| Problem Sensitivity | The ability to tell when something is wrong or is likely to go wrong. It does not involve solving the problem, only recognizing there is a problem. | Idea generation and Reasoning Abilities | 3.375 | 3.375 |
| Rate Control | The ability to time your movements or the movement of a piece of equipment in anticipation of changes in the speed and/or direction of a moving object or scene. | Control Movement Abilities | 4.072 | 4.072 |
| Reaction Time | The ability to quickly respond (with the hand, finger, or foot) to a signal (sound, light, picture) when it appears. | Reaction Time and Speed Abilities | 0 | 0 |
| Response Orientation | The ability to choose quickly between two or more movements in response to two or more different signals (lights, sounds, pictures). It includes the speed with which the correct response is started with the hand, foot, or other body part. | Control Movement Abilities | -2.821 | -2.821 |
| Selective Attention | The ability to concentrate on a task over a period of time without being distracted. | Attentiveness | 0 | 0 |
| Sound Localisation | The ability to tell the direction from which a sound originated. | Auditory and Speech Abilities | 0 | 0 |
| Spatial Orientation | The ability to know your location in relation to the environment or to know where other objects are in relation to you. | Spatial Abilities | 0 | 0 |
| Speech Clarity | The ability to speak clearly so others can understand you. | Auditory and Speech Abilities | -0.25 | -0.25 |
| Speech Recognition | The ability to identify and understand the speech of another person. | Auditory and Speech Abilities | -2.622 | -2.622 |
| Speed of Closure | The ability to quickly make sense of, combine, and organize information into meaningful patterns. | Perceptual Abilities | 4.591 | 4.591 |
| Speed of Limb Movement | The ability to quickly move the arms and legs. | Reaction Time and Speed Abilities | -1.457 | -1.457 |
| Stamina | The ability to exert yourself physically over long periods of time without getting winded or out of breath. | Endurance | 0.948 | 0.948 |
| Static Strength | The ability to exert maximum muscle force to lift, push, pull, or carry objects. | Physical Strength Abilities | 1.268 | 1.268 |
| Time Sharing | The ability to shift back and forth between two or more activities or sources of information (such as speech, sounds, touch, or other sources). | Attentiveness | 0 | 0 |
| Trunk Strength | The ability to use your abdominal and lower back muscles to support part of the body repeatedly or continuously over time without 'giving out' or fatiguing. | Physical Strength Abilities | 0 | 0 |
| Visual Color Discrimination | The ability to match or detect differences between colors, including shades of color and brightness. | Visual Abilities | 3.629 | 3.629 |
| Visualisation | The ability to imagine how something will look after it is moved around or when its parts are moved or rearranged. | Spatial Abilities | 0 | 0 |
| Wrist-Finger Speed | The ability to make fast, simple, repeated movements of the fingers, hands, and wrists. | Reaction Time and Speed Abilities | -0.738 | -0.738 |
| Written Comprehension | The ability to read and understand information and ideas presented in writing. | Verbal Abilities | -0.845 | -0.845 |
| Written Expression | The ability to communicate information and ideas in writing so others will understand. | Verbal Abilities | 0 | 0 |
| R Squared = 0.96 | |  | N=59723 | |

Table X2 UK Skills Lasso Analysis

| ONET Item | Description |  |  | Coefficient |
| --- | --- | --- | --- | --- |
| Active Learning | Understanding the implications of new information for both current and future problem-solving and decision-making. | Process | Basic Skills | 3.207 |
| Active Listening | Giving full attention to what other people are saying, taking time to understand the points being made, asking questions as appropriate, and not interrupting at inappropriate times. | Content | Basic Skills | 0 |
| Complex Problem Solving | Identifying complex problems and reviewing related information to develop and evaluate options and implement solutions. | Complex Problem Solving | Cross Functional Skills | -1.933 |
| Coordination | Adjusting actions in relation to others' actions. | Social Skills | Cross Functional Skills | -4.467 |
| Critical Thinking | Using logic and reasoning to identify the strengths and weaknesses of alternative solutions, conclusions or approaches to problems. | Process | Basic Skills | -1.821 |
| Equipment Maintenance | Performing routine maintenance on equipment and determining when and what kind of maintenance is needed. | Technical Skills | Cross Functional Skills | -1.532 |
| Equipment Selection | Determining the kind of tools and equipment needed to do a job. | Technical Skills | Cross Functional Skills | 0.927 |
| Installation | Installing equipment, machines, wiring, or programs to meet specifications. | Technical Skills | Cross Functional Skills | -0.609 |
| Instructing | Teaching others how to do something. | Social Skills | Cross Functional Skills | -1.512 |
| Judgement and Decision Making | Considering the relative costs and benefits of potential actions to choose the most appropriate one. | Systems Skills | Cross Functional Skills | 0 |
| Learning Strategies | Selecting and using training/instructional methods and procedures appropriate for the situation when learning or teaching new things. | Systems Skills | Cross Functional Skills | -0.324 |
| Management of Financial Resources | Determining how money will be spent to get the work done, and accounting for these expenditures. | Resource Management Skills | Cross functional Skills | 0 |
| Management of Material Resources | Obtaining and seeing to the appropriate use of equipment, facilities, and materials needed to do certain work. | Resource Management Skills | Cross functional Skills | 0 |
| Management of Personell Resources | Motivating, developing, and directing people as they work, identifying the best people for the job. | Resource Management Skills | Cross functional Skills | 0 |
| Mathematics | Using mathematics to solve problems. | Content | Basic Skills | 0 |
| Monitoring | Monitoring/Assessing performance of yourself, other individuals, or organizations to make improvements or take corrective action. | Process | Basic Skills | -5.989 |
| Negotiation | Bringing others together and trying to reconcile differences. | Social Skills | Cross functional Skills | 1.972 |
| Operation Monitoring | Watching gauges, dials, or other indicators to make sure a machine is working properly. | Technical Skills | Cross Functional Skills | 2.377 |
| Operation and Control | Controlling operations of equipment or systems. | Technical Skills | Cross Functional Skills | -0.834 |
| Operations Analysis | Analyzing needs and product requirements to create a design. | Technical Skills | Cross Functional Skills | -0.534 |
| Persuasion | Persuading others to change their minds or behavior. | Social Skills | Cross functional Skills | -1.766 |
| Programming | Writing computer programs for various purposes. | Technical Skills | Cross functional Skills | -0.558 |
| Quality Control Analysis | Conducting tests and inspections of products, services, or processes to evaluate quality or performance. | Technical Skills | Cross functional Skills | -1.525 |
| Reading Comprehension | Understanding written sentences and paragraphs in work related documents. | Content | Basic Skills | 0 |
| Repairing | Repairing machines or systems using the needed tools. | Technical Skills | Cross Functional Skills | 0 |
| Science | Using scientific rules and methods to solve problems. | Content | Basic Skills | 0.721 |
| Service Orientation | Actively looking for ways to help people. | Social Skills | Cross Functional Skills | 0.356 |
| Social Perceptiveness | Being aware of others' reactions and understanding why they react as they do. | Social Skills | Cross Functional Skills | 0 |
| Speaking | Talking to others to convey information effectively. | Content | Basic Skills | 2.306 |
| System Analysis | Determining how a system should work and how changes in conditions, operations, and the environment will affect outcomes. | Systems Skills | Cross Functional Skills | -2.76 |
| System Evaluation | Identifying measures or indicators of system performance and the actions needed to improve or correct performance, relative to the goals of the system. | Systems Skills | Cross Functional Skills | 5.182 |
| Technology Design | Generating or adapting equipment and technology to serve user needs. | Technical Skills | Cross Functional Skills | -2.603 |
| Time Management | Managing one's own time and the time of others. | Resource Management Skills | Cross Functional Skills | 3.48 |
| Troubleshooting | Determining causes of operating errors and deciding what to do about it. | Technical Skills | Cross Functional Skills | 1.72 |
| Writing | Communicating effectively in writing as appropriate for the needs of the audience. | Content | Basic Skills | 0 |
| R squared = 0.80 |  |  | N= | 59723 |

Table X3 UK People, Brains, Brawn Analysis

|  | |
| --- | --- |
|  | UK – EU LFS |
| People | 0.006*** |
|  | (0.001) |
| Brains | -0.100*** |
|  | (0.001) |
| Brawn | 0.007*** |
|  | (0.001) |
| People * Brains | 0.005*** |
|  | (0.001) |
| People*Brawn | -0.001*** |
|  | (0.000) |
| Brains*Brawn | -0.003*** |
|  | (0.001) |
| N | 59575 |
| R Squared | 13% |

***, **, * denotes significance at the 1%, 5% and 10% levels respectively
